# Platinum-Catalyzed Regio- and Stereoselective Diboration of Allenes by 1,8-Diaminonaphthalene-Protected Diboronic Acid (B_2_(dan)_2_)

**DOI:** 10.1021/acs.joc.5c02170

**Published:** 2025-11-08

**Authors:** Yuki Ito, Tairin Kawasaki, Yusuke Yoshigoe, Shinichi Saito

**Affiliations:** Department of Chemistry, Faculty of Science, 26413 Tokyo University of Science, Kagurazaka, Shinjuku, Tokyo 162-8601, Japan

## Abstract

We report a regio- and stereoselective diboration of the terminal C=C bond of allenes by B_2_(dan)_2_ (dan = naphthalene-1,8-diaminato) in the presence of a Pt catalyst. This reaction is applicable to a broad range of substrates, including 1-arylallenes and 1,1-disubstituted allenes. The two boryl groups in the product exhibit distinct reactivities, enabling chemoselective transformations.

## Graphical abstract

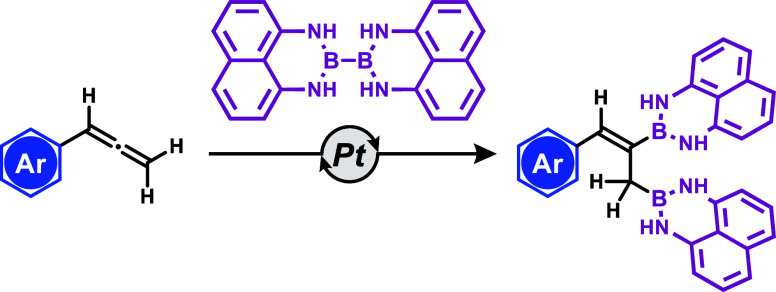

## Introduction

The transition-metal-catalyzed addition of diboronic acid derivatives to unsaturated hydrocarbons has attracted attention in recent years due to its ability to provide useful organodiboron compounds.^1^ Among such transformations, the diboration of allenes is a practical approach for the efficient synthesis of vinyl- and allylboron derivatives. Some challenges, however, remain to be addressed: (1) the control of regioselectivity arising from the presence of two different C=C bonds in allenes and (2) the control of *E*/*Z* stereoselectivity of the resulting alkenes. Miyaura and co-workers reported the first example related to the platinum-catalyzed diboration of allenes with bis­(pinacolato)­diboron (B_2_(pin)_2_), demonstrating the selective addition to the internal double bond (Scheme 1a).^2^ Subsequently, Morken^3^ and Tang^4^ independently achieved the enantioselective diboration at the internal double bond by employing a platinum or palladium catalyst in combination with chiral phosphine ligands. In contrast, reports on the terminal diboration of allenes remain limited. For instance, Yang and Cheng developed the (*Z*)-selective diboration of the terminal C=C bond by employing a palladium catalyst in combination with an alkenyl iodide (Scheme 1b).^5^ Stratakis reported that the use of gold nanoparticles with B_2_(pin)_2_ enabled terminal diboration of mono- and disubstituted allenes, affording mainly the (*Z*)-isomer.^6^ Platinum-catalyzed terminal diboration has also been achieved with only a limited range of substrates. For instance, Chen and co-workers developed the (*Z*)-selective terminal diboration of aminoallenes or alkoxyallenes in the presence of a platinum catalyst.^7^ Furthermore, Santos and co-workers achieved the regioselective terminal diboration of 1,1-disubstituted allenes using an unsymmetrical diboron compound, B­(pin)­B­(dan) (dan = naphthalene-1,8-diaminato), in the presence of a platinum catalyst (Scheme 1c).^8^ In reactions involving 1-arylallenes, however, precise control over both the regio- and stereoselectivity turned out to be difficult. Therefore, the development of a selective diboration reaction that can be applied to a broader range of substrates is highly demanding.

**Scheme 1:**
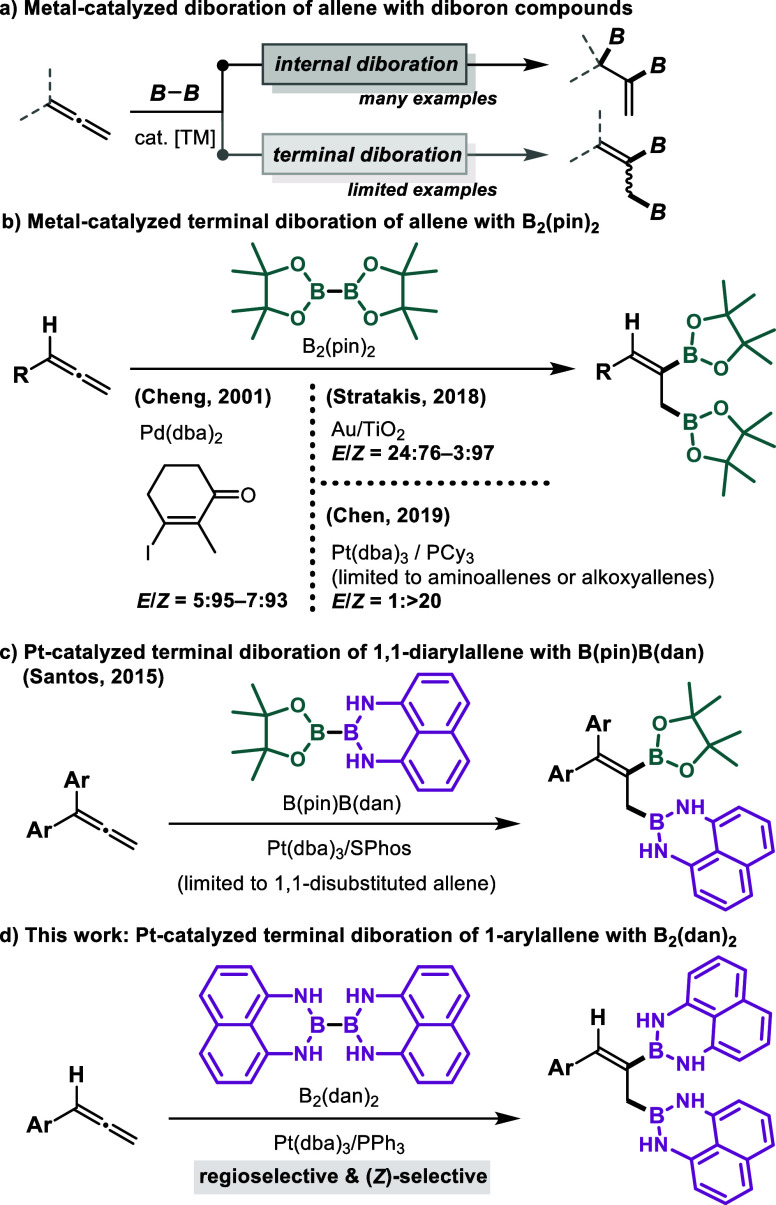
Diboration of Allenes with Diboron Compounds

We have been interested in the chemistry of B­(dan) derivatives.^9^ The B­(dan) group, developed by Suginome and co-workers as a protected boronic acid,^10^ has recently been shown to be directly applicable to cross-coupling reactions, as demonstrated independently by our group^9a,9b^ and Yoshida–Tsuchimoto group.^11^ Furthermore, we recently reported that B_2_(dan)_2_ (**2**) was readily available from B_2_(OH)_4_,^9c^ which could be used as an efficient borylation reagent of styrenes^9c^ or diborylating reagent of alkynes^9d^ in the presence of transition-metal catalysts. Based on these findings, we envisioned that the diboration of allenes using B_2_(dan)_2_ would proceed in the presence of a platinum catalyst, providing stable yet reactive diborated compounds. In this article, we report the platinum-catalyzed terminal diboration of allenes using B_2_(dan)_2_ (Scheme 1d).

## Results and Discussion

We examined the diboration of 4-methoxyphenylallene (**1a**) with B_2_(dan)_2_ (**2**) in the presence of a platinum catalyst. Reaction conditions previously optimized for the diboration of alkynes using B_2_(dan)_2_ ^9d^ were applicable to the reaction of **1a**. Stirring a mixture of **1a** (0.10 mmol), **2** (1.1 equiv), Pt­(dba)_3_ (3.0 mol %), and PPh_3_ (3.0 mol %) in toluene at 110 °C for 30 min led to (*Z*)-selective diboration of the terminal C=C bond, affording **3a** in 93% isolated yield (Scheme 2). Although a trace amount of an isomer was detected in the crude mixture by ^1^H NMR spectroscopy, the pure product could be isolated by silica gel column chromatography. In contrast, when B_2_(pin)_2_ was employed under the same conditions, the combined yield of the products decreased (61%) and the internal diborated product (**3r′**) was formed as the major isomer in 40% yield. According to the report by Santos and co-workers, the diboration of phenylallene (**1b**) with B­(pin)­B­(dan) under similar conditions (Pt­(dba)_3_ (4.0 mol %), PPh_3_ (6.0 mol %), toluene at 80 °C for 24 h) afforded **3s** in 10% yield and **3s′** in 42% yield.^8^ These results demonstrated that B_2_(dan)_2_ enables the terminal diboration of 1-arylallenes in the presence of a Pt catalyst with high regio- and stereoselectivity, which has not been achieved using other diboron compounds.

**Scheme 2:**
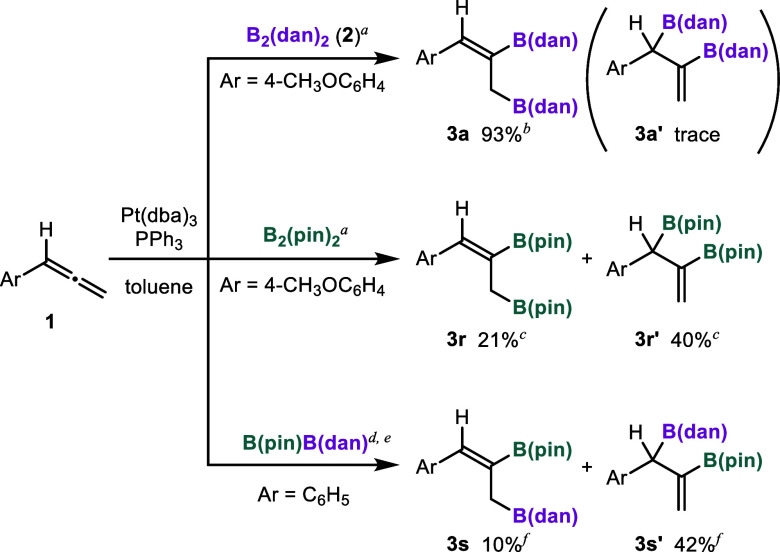
Comparison of the Reactivity of B_2_(dan)_2_ with Other Diboron Compounds

a
**1** (0.10 mmol), diboron reagent (1.1 equiv), Pt­(dba)_3_ (3.0 mol %), PPh_3_ (3.0 mol %), toluene (1 mL) at 110 °C for 30 min.
b Isolated yield.
c Determined by ^1^H NMR analysis using CH_3_NO_2_ as an internal standard.
d Reported by Santos et al.^8^

e
**1** (0.163 mmol), B­(pin)­B­(dan) (0.136 mmol), Pt­(dba)_3_ (4.0 mol %), PPh_3_ (6.0 mol %), toluene (1 mL) at 80 °C for 24 h.
f Determined by GC analysis.

Next, we investigated the substrate scope of this reaction (Scheme 3). The scalability of the reaction was demonstrated by using a larger amount of **1a** (10 mmol), affording **3a** in 88% yield after stirring for 1 h in the presence of 1 mol % of Pt­(dba)_3_/PPh_3_. The reaction proceeded smoothly with phenylallene (**1b**) and 4-methylphenylallene (**1c**), providing the corresponding products **3b** and **3c** in 70% and 81% yields, respectively. When an electron-withdrawing group such as bromine (**1d**) or trifluoromethyl group (**1e**) was introduced at the para position of the phenyl group, the diborated product **3d** or **3e** was isolated in excellent yields of 91% or 93%, respectively. In contrast, substrates bearing an ethoxycarbonyl group (**1f**) or a cyano group (**1g**) showed slightly reduced reactivity. Under the standard reaction conditions, **3f** was isolated in 74% yield after 1 h, while **3g** was isolated in 60% yield after 30 min. These results imply that the coordination of the ester or cyano group to the platinum catalyst would result in the decreased rate of the reaction, which is consistent with our previous report.^9d^ 2-Methoxyphenylallene (**1h**), 3-methoxyphenylallene (**1i**), and mesitylallene (**1j**) also reacted smoothly, affording products in 82–86% yields. The results indicate that the steric effect of the substituents bound to the aryl group has a minimal impact on the reactivity. It is noteworthy that the diborated compounds synthesized in this study were bench stable and readily purified by silica gel column chromatography.

**Scheme 3:**
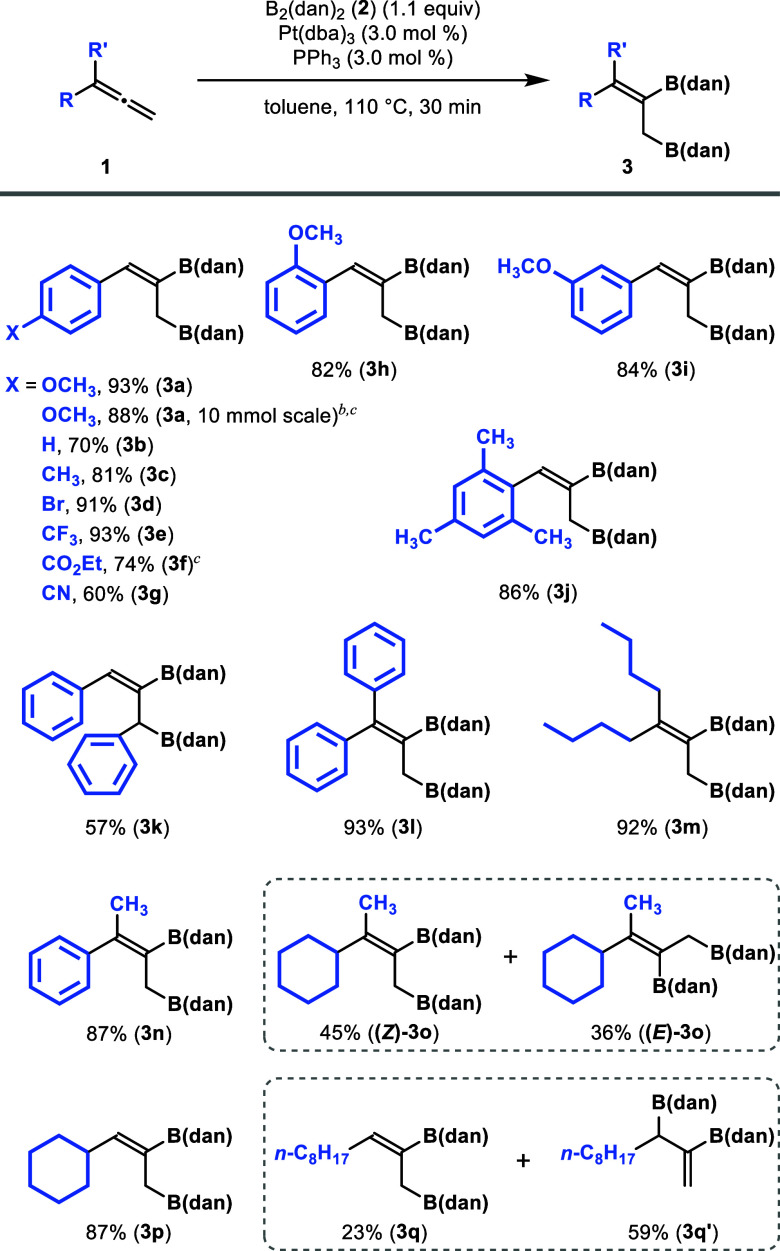
Pt-Catalyzed Diboration of Allenes with B_2_(dan)_2_ ^a^

a General reaction conditions: a mixture of **1** (0.10 mmol), **2** (1.1 equiv), Pt­(dba)_3_ (3.0 mol %), and PPh_3_ (3.0 mol %) in toluene (1 mL) was stirred for 30 min at 110 °C.
b Pt­(dba)_3_ (1.0 mol %)/PPh_3_ (1.0 mol %) was used.
c The reaction time was 1 h.

The scope of this reaction was further examined by using other allenes. The reaction of 1,3-diphenylallene (**1k**) afforded the corresponding product (**3k**) in 57% yield. The reactions of symmetrical 1,1-disubstituted allenes **1l** and **1m** proceeded smoothly to afford the corresponding diborated products in high yields (93% and 92%) with excellent regioselectivity. Although the reaction of unsymmetrical allene **1n** was expected to give a mixture of *E*/*Z* isomers, only the terminal diborated (*Z*)-isomer was obtained in 87% yield. Unexpectedly, the reaction of 1-cyclohexyl-1-methylallene (**1o**) gave a mixture of *E*/*Z* isomers: **(*Z*)-3o** and **(*E*)-3o** were isolated in 45% and 36% yields, respectively. Interestingly, the reaction of cyclohexylallene **1p** led to (*Z*)-selective terminal diboration, providing **3p** in 87% yield. These results indicate that this reaction can be applied to a wide range of allenes, including 1-arylallenes, 1,3-disubstituted allenes, 1,1-disubstituted allenes, and monosubstituted allenes with a secondary alkyl group. When octylallene **1q** was used, the internal diborated product **3q′** was obtained in 59% yield as the major isomer, while the terminal diborated product **3q** was isolated in 23% yield under the standard conditions. This result implies that steric hindrance plays an important role for the observed regioselectivity.

The diborated compounds synthesized in this study are valuable synthetic intermediates (Scheme 4). When Chan–Lam–Evans-type coupling^12^ of **3a** was attempted with Cu­(OAc)_2_ (5.0 mol %), (*t*-BuO)_2_ (2.0 equiv), and *N*-methylaniline (4.0 equiv) at 100 °C, the coupling product **4a** was obtained in 58% yield. Notably, the allyl-B­(dan) moiety was selectively transformed into a C–N bond. The remaining alkenyl-B­(dan) group in **4a** was directly converted to styryl group via Suzuki–Miyaura coupling,^11c^ demonstrating the orthogonal reactivity of the two B­(dan) groups. Both B­(dan) groups in **3a** could also be directly converted into the corresponding B­(pin) derivative **3r**.^13^ Treatment of **3a** with KO*t*-Bu resulted in selective protodeboration of the allyl-B­(dan) to afford **6a**. This compound was subsequently transformed into trisubstituted alkene **7a** in 92% yield by direct Suzuki–Miyaura coupling.^9b^

**Scheme 4:**
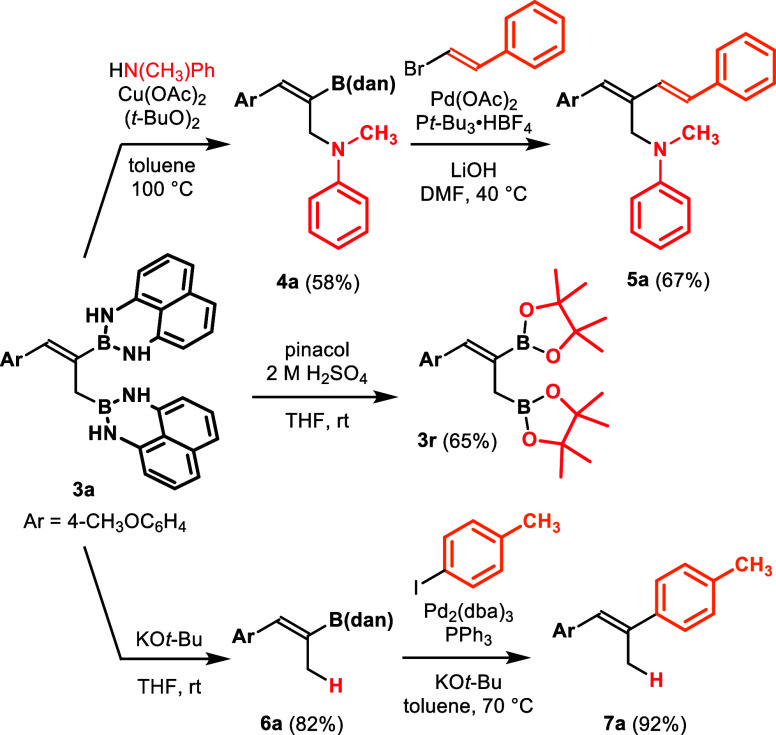
Derivatization of **3a**

We assume that the mechanism of this reaction is similar to that reported for the platinum-catalyzed diboration of allenes using other diboron compounds (Scheme 5). Thus, the oxidative addition of B_2_(dan)_2_ to a Pt(0) species (**I**) would generate a Pt­(II) complex (**II**), which then coordinate to the less sterically hindered terminal double bond of the allene to form intermediate **III**. In this intermediate, the substituent R and the platinum center would adopt an anti-configuration to minimize steric hindrance, which would determine the stereochemistry of the product to afford the (*Z*)-isomer. Subsequently, the insertion of the allene into the Pt–B bond would give the η^1^-allyl platinum intermediate (**IV**), which would further rearrange into a more stable η^3^-allyl platinum complex (**Va**).^14^ This complex would undergo reductive elimination to afford **3**. Alternatively, a pathway from **IV** to **Vb**, followed by reductive elimination, would lead to a regioisomer **3′**. This route, however, would likely be suppressed due to steric hindrance between the B­(dan) group bound to platinum and substituent R on the allene.

**Scheme 5:**
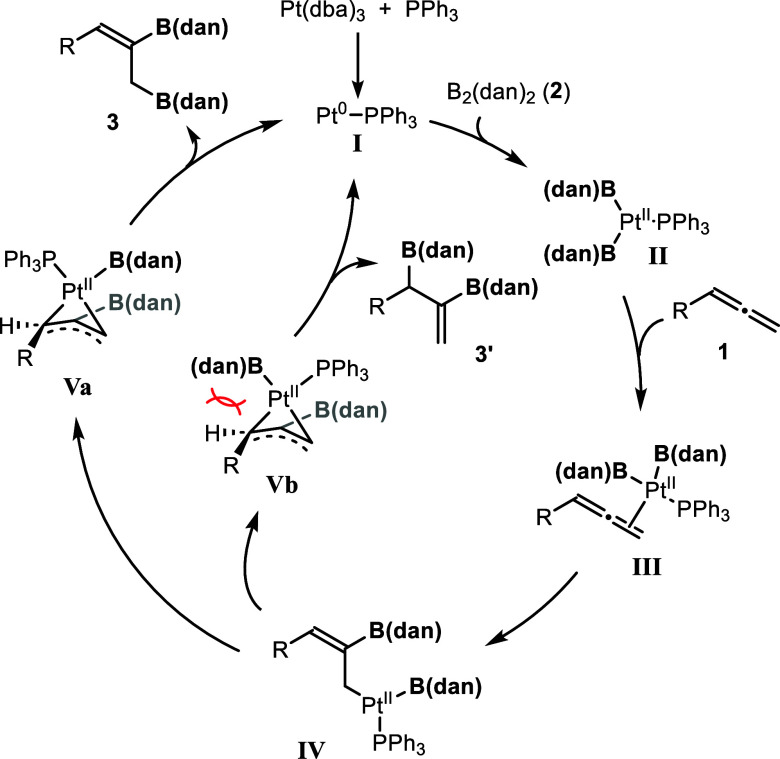
Proposed Mechanism for the Pt-Catalyzed Diboration of Allenes with B_2_(dan)_2_

## Conclusion

In conclusion, we developed a terminal diboration of allenes using a platinum catalyst and B_2_(dan)_2_. This reaction proceeds with high regio- and stereoselectivity across a wide range of substrates, including 1-arylallenes that have posed challenges in terms of selectivity. The diborated compounds possess both alkenyl-B­(dan) and allyl-B­(dan) moieties, which could be derivatized in chemoselective manner. The study provides a complementary approach to the previously reported internal diboration of allenes, thereby contributing to the expansion of the chemistry of organodiboron compounds.

## Supplementary Material



## Data Availability

The data underlying this study are available in the published article and its Supporting Information.

## Notes

General information, experimental procedures, characterization data, and NMR spectra for novel compounds (PDF)

## †

Center for Education, Faculty of Engineering, Chiba Institute of Technology, Chiba 275-0023, Japan
