# Supplementary material for: Platinum-Catalyzed Regio- and Stereoselective Diboration of Allenes by 1,8-Diaminonaphthalene-Protected Diboronic Acid (B2(dan)2)
Source: J Org Chem. 2025 Nov 8;90(46):16568–72. doi: 10.1021/acs.joc.5c02170 (PMC12645474; doi:10.1021/acs.joc.5c02170)
Supplement: Supplementary file 1 [file jo5c02170_si_001.pdf]

Supporting Information

Platinum-Catalyzed Regio- and Stereoselective Diboration of Allenes by 1,8-Diaminonaphthalene-Protected Diboronic Acid (B_2(dan)$_2$)

Yuki Ito, Tairin Kawasaki, Yusuke Yoshigoe, and Shinichi Saito*

Department of Chemistry, Faculty of Science, Tokyo University of Science
Kagurazaka, Shinjuku, Tokyo 162-8601, Japan

*E-mail: ssaito@rs.tus.ac.jp

Table of Contents

General Information S2
Experimental Section S3
I. Procedure for Preparation of Allenes (**1**) S3
II. Procedure for Preparation of B_2(dan)$_2$ (**2**) S10
III. Comparison of the Reactivity of B_2(dan)$_2$ with Other Diboron Compounds S11
IV. General Procedure for Platinum-Catalyzed Diboration of Allenes (**1**) S11
V. Characterization Data of **3** S12
VI. ^{1}H-^{1}H NOESY spectra of **3a**, **3n**, **(*Z*)-3o**, **(*E*)-3o**, **3p** and **3q** S22
VII. Derivatization of **3a** S25
References S30
Copy of NMR Spectra S32

General Information

Unless otherwise noted, all reactions were carried out in oven- or flame-dried glassware under an inert atmosphere of argon. 1,4-dioxane, THF, Et_2O, toluene and DMF of anhydrous grade were purchased from commercial sources. Unless otherwise noted, other reagents and solvents were commercially available and used without further purification. An oil bath was used as the heat source, and the external temperature was reported. NMR spectra were recorded on a 400 MHz spectrometer at 298 K. Chemical shifts were reported in delta units (δ) relative to residual $CHCl_3$ in $CDCl_3$ (7.24 ppm) or DMSO in DMSO-d_6 (2.50 ppm) for ^{1}H NMR, $CHCl_3$ in $CDCl_3$ (77.23 ppm) or DMSO in DMSO-d_6 (39.52 ppm) for ^{13}C NMR. ^{11}B{^{1}H} NMR spectra were measured in quartz NMR tube, and the chemical shifts were referenced to the ^{11}B{^{1}H} signal of $BF_3{\cdot}OEt_2$ (0.00 ppm) as an external standard. Multiplicity is indicated by s (singlet), d (doublet), t (triplet), q (quartet), m (multiplet) and br (broad). Coupling constants, *J*, are reported in Hertz. Structural assignments were made with additional information from NOESY experiments. IR spectra were recorded on a Fourier transform infrared spectrometer using a diamond ATR module. Thin layer chromatography was performed on Merck silica gel 60F-254 plates. Column chromatography was performed using Kanto Chemical silica gel 60N (spherical, neutral 40–50 μm). High-resolution mass spectra (HRMS) were obtained on a quadrupole time-of-flight (TOF) mass analyzer.

Experimental Section

I. Procedure for Preparation of Allenes (1)

Synthesis of 1a-j and 1q

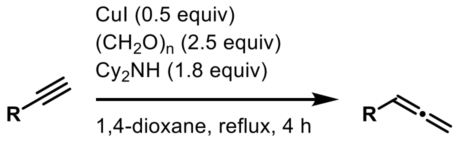

1

General Procedure

Following the modified procedure by Kuang and Ma,[1] a mixture of alkyne (Y mmol, 1.0 equiv), CuI (0.5 × Y mmol, 0.5 equiv), paraformaldehyde (2.5 × Y mmol, 2.5 equiv) and Cy_2NH (1.8 × Y mmol 1.8 equiv) in 1,4-dioxane (0.25 M, based on alkyne) was refluxed for 4 h. The resulting mixture was diluted with water and extracted with CH_2Cl_2 (× 3). The combined organic phase was dried over Na_2SO_4 and concentrated under reduced pressure. The residue was purified by silica gel column chromatography to afford **1**.

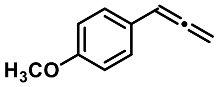

1a

1-Methoxy-4-(propa-1,2-dien-1-yl)benzene (**1a**): **1a** (400 mg, 2.7 mmol, 54%) was synthesized from 4-ethynylanisole (660 mg, 5.0 mmol). Purified by silica gel column chromatography (hexane). Pale yellow oil. ^{1}H NMR ($CDCl_3$, 400 MHz): δ 7.21 (d, J = 8.8 Hz, 2H), 6.84 (d, J = 8.8 Hz, 2H), 6.11 (t, J = 6.9 Hz, 1H), 5.10 (d, J = 6.8 Hz, 2H), 3.78 (s, 3H). The NMR data matched those reported previously.[2]

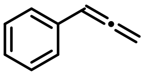

1b

Propa-1,2-dien-1-ylbenzene (**1b**): **1b** (230 mg, 2.0 mmol, 40%) was synthesized from ethynylbenzene (510 mg, 5.0 mmol). Purified by silica gel column chromatography (hexane). Colorless oil. ^{1}H NMR ($CDCl_3$, 400 MHz): δ 7.31-7.28 (m, 4H), 7.21-7.17 (m, 1H), 6.16 (t, J = 6.9 Hz, 1H), 5.14 (d, J = 6.8 Hz, 2H). The NMR data matched those reported previously.[2]

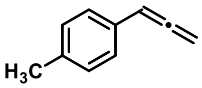

1c

1-Methyl-4-(propa-1,2-dien-1-yl)benzene (**1c**): **1c** (300 mg, 2.3 mmol, 46%) was synthesized from 4-ethynyltoluene (580 mg, 5.0 mmol). Purified by silica gel column chromatography (hexane). Colorless oil. ^{1}H NMR ($CDCl_3$, 400 MHz): δ 7.32-7.10 (m, 4H), 6.15 (t, J = 6.8 Hz, 1H), 5.13 (d, J = 6.8 Hz, 2H), 2.53 (s, 3H). The NMR data matched those reported previously.[3]

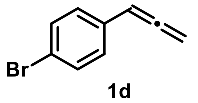

1d

1-Bromo-4-(propa-1,2-dien-1-yl)benzene (**1d**): **1d** (230 mg, 1.2 mmol, 40%) was synthesized from 1-bromo-4-ethynylbenzene (540 mg, 3.0 mmol). Purified by silica gel column chromatography (hexane). Colorless oil. ^{1}H NMR ($CDCl_3$, 400 MHz): δ 7.40 (d, *J* = 8.3 Hz, 2H), 7.14 (d, *J* = 8.5 Hz, 2H), 6.09 (t, *J* = 6.9 Hz, 1H), 5.13 (d, *J* = 7.0 Hz, 2H). The NMR data matched those reported previously.[2]

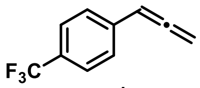

1e

1-(Propa-1,2-dien-1-yl)-4-(trifluoromethyl)benzene (**1e**): **1e** (320 mg, 1.8 mmol, 36%) was synthesized from 1-ethynyl-4-(trifuloromethyl)benzene (850 mg, 5.0 mmol). Purified by silica gel column chromatography (hexane). Pale yellow oil. ^{1}H NMR ($CDCl_3$, 400 MHz): δ 7.53 (d, *J* = 8.3 Hz, 2H), 7.37 (d, *J* = 8.3 Hz, 2H), 6.17 (t, *J* = 6.9 Hz, 1H), 5.19 (d, *J* = 6.8 Hz, 2H). The NMR data matched those reported previously.[4]

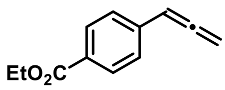

1f

Ethyl 4-(propa-1,2-dien-1-yl)benzoate (**1f**): **1f** (430 mg, 2.3 mmol, 46%) was synthesized from ethyl 4-ethynyl-benzoate (870 mg, 5.0 mmol). Purified by silica gel column chromatography (hexane/AcOEt = 20/1). Pale yellow oil. ^{1}H NMR ($CDCl_3$, 400 MHz): δ 7.96 (d, *J* = 8.4 Hz, 2H), 7.32 (d, *J* = 8.3 Hz, 2H), 6.18 (t, *J* = 6.8 Hz, 1H), 5.18 (d, *J* = 6.8 Hz, 2H), 4.35 (q, *J* = 7.2 Hz, 2H), 1.37 (t, *J* = 7.1 Hz, 3H). The NMR data matched those reported previously.[5]

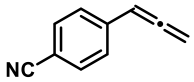

1g

4-(Propa-1,2-dien-1-yl)benzonitrile (**1g**): **1g** (390 mg, 2.8 mmol, 56%) was synthesized from 4-ethynyl-benzonitrile (640 mg, 5.0 mmol). Purified by silica gel column chromatography (hexane/AcOEt = 10/1). Yellow oil. ^{1}H NMR ($CDCl_3$, 400 MHz): δ 7.55 (d, *J* = 8.5 Hz, 2H), 7.34 (d, *J* = 8.3 Hz, 2H), 6.15 (t, *J* = 6.8 Hz, 1H), 5.21 (d, *J* = 6.8 Hz, 2H). The NMR data matched those reported previously.[6]

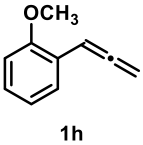

1h

1-Methoxy-2-(propa-1,2-dien-1-yl)benzene (**1h**): **1h** (330 mg, 2.3 mmol, 46%) was synthesized from 2-ethynyl-anisole (660 mg, 5.0 mmol). Purified by silica gel column chromatgraphy (hexane). Colorless oil. ^{1}H NMR ($CDCl_3$, 400 MHz): δ 7.38 (dd, *J* = 7.5, 1.5 Hz, 1H), 7.16 (td, *J* = 7.8, 1.3 Hz, 1H), 6.91 (td, *J* = 7.5, 0.9 Hz, 1H), 6.84 (dd, *J* = 8.3, 0.8 Hz, 1H), 6.55 (t, *J* = 6.9 Hz, 1H), 5.09 (d, *J* = 7.0 Hz, 2H), 3.83 (s, 3H). The NMR data matched those reported previously.[4]

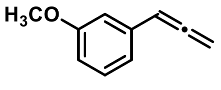

1i

1-Methoxy-3-(propa-1,2-dien-1-yl)benzene (**1i**): **1i** (360 mg, 2.4 mmol, 49%) was synthesized from 3-ethynylanisole (660 mg, 5.0 mmol). Purified by silica gel column chromatography (hexane). Colorless oil. ^{1}H NMR ($CDCl_3$, 400 MHz): δ 7.21 (t, J = 7.9 Hz, 1H), 6.89-6.85 (m, 2H), 6.75 (dd, J = 8.1, 2.1 Hz, 1H), 6.13 (t, J = 6.8 Hz, 1H), 5.14 (d, J = 6.8 Hz, 2H), 3.80 (s, 3H). The NMR data matched those reported previously.[2]

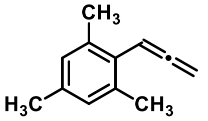

1j

1,3,5-Trimethyl-2-(propa-1,2-dien-1-yl)benzene (**1j**): **1j** (52 mg, 0.33 mmol, 7%) was synthesized from 1-ethynyl-2,4,6-trimethylbenzene (720 mg, 5.0 mmol). Purified by silica gel column chromatography (hexane). Colorless oil. ^{1}H NMR ($CDCl_3$, 400 MHz): δ 6.84 (s, 2H), 6.21 (t, J = 7.0 Hz, 1H), 4.88 (d, J = 7.0 Hz, 2H), 2.31 (s, 6H), 2.24 (s, 3H). The NMR data matched those reported previously.[7]

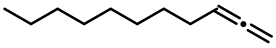

1q

Undeca-1,2-diene (**1q**): **1q** (300 mg, 2.0 mmol, 40%) was synthesized from 1-decyne (690 mg, 5.0 mmol). Purified by silica gel column chromatography (hexane). Colorless oil. ^{1}H NMR ($CDCl_3$, 400 MHz): δ 5.07 (q, J = 6.8 Hz, 1H), 4.63 (dt, J = 6.8, 3.2 Hz, 2H), 1.97 (qt, J = 7.2, 3.2 Hz, 2H), 1.41-1.25 (m, 12H), 0.86 (t, J = 6.9 Hz, 3H). The NMR data matched those reported previously.[8]

Synthesis of 1k

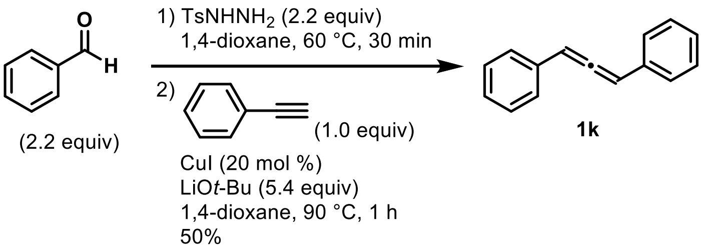

Following the procedure by Wang,[9] a mixture of benzaldehyde (1.1 g, 10 mmol, 2.2 equiv) and $TsNHNH_2$ (1.9 g, 10 mmol, 2.2 equiv) in 1,4-dioxane (10 ml) was stirred for 30 min at 60 ºC. Then a mixture of CuI (170 mg, 0.91 mmol, 20 mol %), LiO*t*-Bu (2.0 g, 24 mmol, 5.4 equiv) and ethynylbenzene (460 mg, 4.6 mmol, 1.0 equiv) in 1,4-dioxane (40 ml) was added. After stirring for 1 h at 90 ºC, the resulting mixture was filtered through a short pad of

silica gel and concentrated under reduced pressure. The residue was purified by silica gel column chromatography (hexane) to afford **1k** (440 mg, 2.3 mmol, 50%).

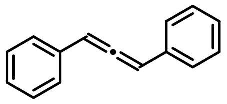

1k

1,3-Diphenylpropa-1,2-diene (**1k**): Pale yellow oil. ^{1}H NMR ($CDCl_3$, 400 MHz): δ 7.36-7.18 (m, 10H), 6.58 (s, 2H). The NMR data matched those reported previously.[10]

Synthesis of 1l, 1n and 1p

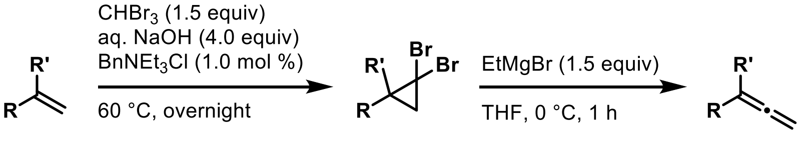

General Procedure

Following the modified procedure by Krische,[11] to a mixture of alkene (Y mmol, 1.0 equiv) and $BnNEt_3Cl$ (0.010 × Y mmol, 1.0 mol %) in $CHBr_3$ (1.5 × Y mmol, 1.5 equiv) was added 50% aq. NaOH (4.0 × Y mmol, 4.0 equiv) dropwise. The reaction mixture was stirred overnight at 60 ºC. The mixture was quenched by water and extracted with CH_2Cl_2. The organic phase was dried over Na_2SO_4 and concentrated under reduced pressure. The residue was purified by silica gel column chromatography to afford 1,1-dibromocyclopropane derivatives (**S1**).

A flame-dried round-bottomed flask was charged with **S1** (Y mmol, 1.0 equiv) and THF (1.0 M, based on **S1**). 1 M EtMgBr/THF (1.5 × Y mmol, 1.5 equiv) was added dropwise at 0 ºC. After stirring for 1 h at the same temperature, the resulting mixture was quenched by 3 M aq. HCl and extracted with Et_2O (× 3). The combined organic phase was dried over Na_2SO_4 and concentrated under reduced pressure. The residue was purified by silica gel column chromatography (hexane) to afford **1**.

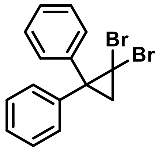

S1l

(2,2-Dibromocyclopropane-1,1-diyl)dibenzene (**S1l**): **S1l** (360 mg, 1.0 mmol, 40%) was synthesized from 1,1-diphenylethylene (450 mg, 2.5 mmol). Purified by silica gel column chromatography (hexane). White solid. ^{1}H NMR ($CDCl_3$, 400 MHz): δ 7.51-7.49 (m, 4H), 7.31-7.28 (m, 4H), 7.23-7.19 (m, 2H), 2.46 (s, 2H). The NMR data matched those reported previously.[3]

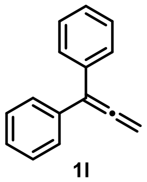

1l

Propa-1,2-diene-1,1-diyldibenzene (**1l**): **1l** (120 mg, 0.64 mmol, 80%) was synthesized from **S1l** (280 mg, 080 mmol). Purified by silica gel column chromatography (hexane). Pale yellow oil. ^{1}H NMR ($CDCl_3$, 400 MHz): δ 7.37-7.25 (m, 10H), 5.25 (s, 2H). The NMR data matched those reported previously.[3]

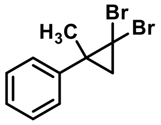

S1n

(2,2-Dibromo-1-methylcyclopropyl)benzene (**S1n**): **S1n** (610 mg, 2.1 mmol, 70%) was synthesized from α-methylstyrene (360 mmol, 3.0 mmol). Purified by silica gel column chromatography (hexane). White solid. ^{1}H NMR ($CDCl_3$, 400 MHz): δ 7.36-7.24 (m, 5H), 2.15 (d, J = 7.5 Hz, 1H), 1.76 (d, J = 7.5 Hz, 1H), 1.70 (s, 3H). The NMR data matched those reported previously.[3]

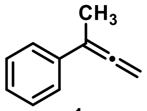

1n

Buta-2,3-dien-2-ylbenzene (**1n**): **1n** (130 mg, 1.0 mmol, 50%) was synthesized from **S1n** (580 mg, 2.0 mmol). Purified by silica gel column chromatography (hexane). Colorless oil. ^{1}H NMR ($CDCl_3$, 400 MHz): δ 7.41 (d, J = 7.6 Hz, 2H), 7.32 (t, J = 7.6 Hz, 2H), 7.19 (t, J = 7.3 Hz, 1H), 5.02 (q, J = 3.2 Hz, 2H), 2.09 (t, J = 3.2 Hz, 3H). The NMR data matched those reported previously.[3]

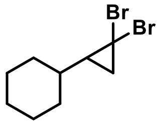

S1p

(2,2-Dibromocyclopropyl)cyclohexane (**S1p**): **S1p** was synthesized from vinylcyclohexane (1.1 g, 10 mmol). The title compound was not isolated in pure form after silica gel column chromatography (hexane). The residue was used for the next reaction without further purification.

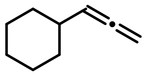

1p

Propa-1,2-dien-1-ylcyclohexane (**1p**): **1p** (140 mg, 1.2 mmol, 18% over 2 steps) was synthesized from **S1p** (1.1 g, 4.0 mmol). Purified by silica gel column chromatography (hexane). Colorless oil. ^{1}H NMR ($CDCl_3$, 400 MHz): δ 5.07 (q, J = 6.4 Hz, 1H), 4.66 (dd, J = 6.6, 3.1 Hz, 2H), 1.99-1.93 (m, 1H), 1.76-1.58 (m, 5H), 1.30-1.03 (m, 5H). The NMR data matched those reported previously.[12]

Synthesis of 1m

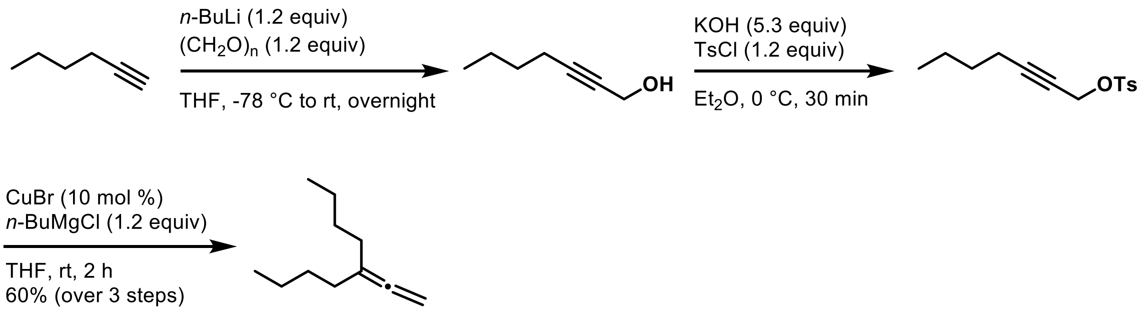

1m

Following the procedure by Ryu,[13] to a solution of 1-hexyne (2.3 mL, 20 mmol, 1.0 equiv) in THF (15 mL) was added 1.6 M *n*-BuLi/hexane (15 mL, 24 mmol, 1.2 equiv) dropwise at -78 ºC. After stirring for 1 h at 0 ºC, the solution was cooled to -78 ºC again and $(CH_2O)_n$ was added in one portion. The reaction mixture was stirred overnight at room temperature. The resulting mixture was quenched by sat. aq. NH_4Cl and the aqueous phase was extracted with Et_2O (× 3). The combined organic phase was washed with brine and dried over $MgSO_4$. Solvents were removed under reduced pressure to afford 2-heptyn-1-ol (2.1 g), which was used for the next reaction without further purification.

To a mixture of 2-heptyn-1-ol (2.1 g) and crushed KOH (6.0 g, 11 mmol, 5.3 equiv) in Et_2O (32 mL) was added TsCl (4.6 g, 24 mmol, 1.2 equiv) at 0 ºC. The reaction mixture was stirred for 30 min at the same temperature. The resulting mixture was poured into ice-cold water, and the aqueous phase was extracted with Et_2O (× 3). The combined organic phase was washed with brine and dried over $MgSO_4$. Solvents were removed under reduced pressure to afford hept-2-yn-1-yl 4-methylbenzenesulfonate (5.8 g), which was used for the next reaction without further purification.

To a mixture of hept-2-yn-1-yl 4-methylbenzenesulfonate (5.8 g) and CuBr (290 mg, 2.0 mmol, 10 mol %) in THF (40 mL) was added 2 M *n*-BuMgCl/Et_2O (12 mL, 24 mmol, 1.2 equiv) dropwise. After stirring for 2 h, the resulting mixture was quenched by sat. aq. NH_4Cl and the aqueous phase was extracted with Et_2O (× 3). The combined organic phase was washed with brine and dried over $MgSO_4$. Solvents were removed under reduced pressure. The residue was purified by silica gel column chromatography (hexane) to afford **1m** (1.8 g, 12 mmol, 60% over 3 steps).

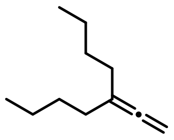

1m

5-Vinylidenenonane (1m): Colorless oil. ^{1}H NMR ($CDCl_3$, 400 MHz): δ 4.61 (quintet, J = 7.2 Hz, 2H), 1.93-1.88 (m, 4H), 1.43-1.25 (m, 8H), 0.88 (t, J = 7.3 Hz, 6H). The NMR data matched those reported previously.[14]

Synthesis of 1o

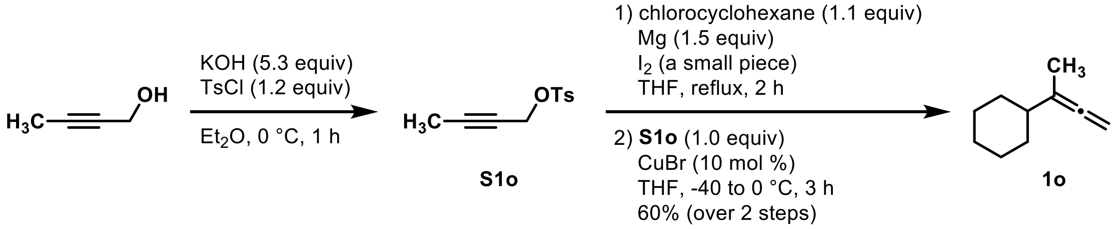

Following the procedure by Tsuji,[15] to a solution of 2-butyn-1-ol (350 mg, 5.0 mmol, 1.0 equiv) in Et_2O (20 ml) was added TsCl (1.1 g, 6.0 mmol, 1.2 equiv) and crushed KOH (1.5 g, 26 mmol, 5.3 equiv) at 0 ºC. After stirring for 1 h at the same temperature, the resulting mixture was poured into ice-cold water. The aqueous phase was extracted with Et_2O (× 3). The combined organic phase was washed with brine and dried over $MgSO_4$. Solvents were removed under reduced pressure. The residue (**S1o**) was used for the next reaction without further purification.

A two-necked flask was charged with Mg turnings (180 mg, 7.5 mmol, 1.5 equiv). Iodine (a small piece) and THF (6 ml) were added, and the mixture was stirred for 5 min. Chlorocyclohexane (650 mg, 5.5 mmol, 1.1 equiv) was added dropwise and the mixture was refluxed for 2 h. To another flask which was charged with **S1o**, CuBr (72 mg, 0.50 mmol, 10 mol %) and THF (10 ml) was added CyMgCl/THF dropwise at -40 ºC. The reaction mixture was gradually warmed to 0 ºC and stirred for 3 h. The reaction was quenched by addition of sat. aq. NH_4Cl. The aqueous phase was extracted with Et_2O (× 3). The combined organic phase was washed with brine and dried over $MgSO_4$. Solvents were removed under reduced pressure. The residue was purified by silica gel column chromatography (hexane) to afford **1o** (410 mg, 3.0 mmol, 60% over 2 steps).

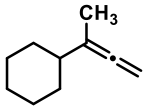

1o

Buta-2,3-dien-2-ylcyclohexane (**1o**): Colorless oil. ^1H NMR ($CDCl_3$, 400 MHz): δ 4.57 (quintet, J = 3.0 Hz, 2H), 1.83-1.63 (m, 9H), 1.31-1.01 (m, 5H). The NMR data matched those reported previously.[16]

II. Procedure for Preparation of $B_2(dan)_2$ (2)[13]

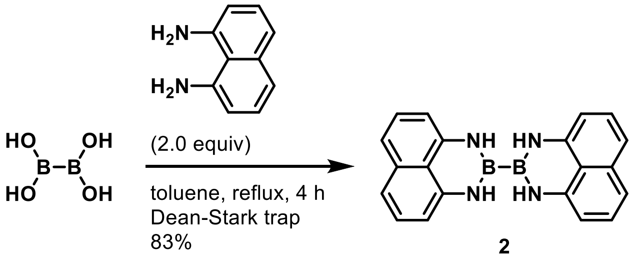

A mixture of $B_2(OH)_4$ (5.8 g, 65 mmol, 1.0 equiv) and sublimed 1,8-diaminonaphthalene (21 g, 130 mmol, 2.0 equiv) in toluene (130 mL) was refluxed for 4 h with a Dean-Stark trap. The resulting precipitate was collected by filtration. The filter cake was purified by recrystallization (AcOEt) to afford **2** (18 g, 54 mmol, 83%).

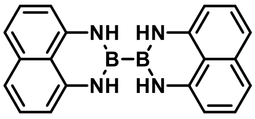

2

$B_2(dan)_2$ (**2**): White solid. ^1H NMR ($CDCl_3$, 400 MHz): δ 7.09 (t, J = 7.8 Hz, 4H), 7.01 (dd, J = 8.4, 0.9 Hz, 4H), 6.30 (dd, J = 7.3, 0.8 Hz, 4H), 5.95 (s, 4H). The NMR data matched those reported previously.[17]

III. Comparison of the Reactivity of $B_2(dan)_2$ with Other Diboron Compounds

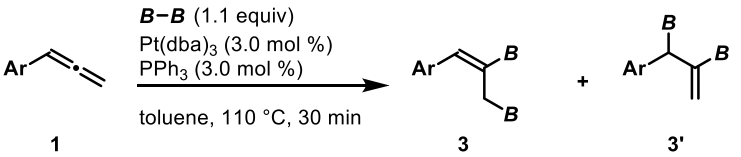

A mixture of $Pt(dba)_3$ (2.7 mg, 0.0030 mmol, 3.0 mol %), PPh_3 (0.79 mg, 0.0030 mmol, 3.0 mol %), ***B-B*** (0.11 mmol, 1.1 equiv) and allene **1** (0.10 mmol, 1.0 equiv) in toluene (1.0 mL) was stirred for 30 min at 110 °C. The yield was determined by ^1H NMR analysis using CH_3NO_2 as an internal standard. **3a** was isolated by silica gel column chromatography (hexane/AcOEt = 10/1). The NMR data of **3r**[18] and **3r'**[19] was previously reported. The reaction with B(pin)B(dan) was reported by Santos and coworkers.[20]

IV. General Procedure for Platinum-Catalyzed Diboration of Allenes (1)

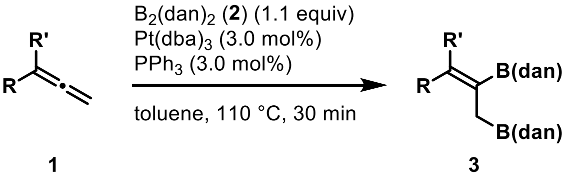

A mixture of $Pt(dba)_3$ (2.7 mg, 0.0030 mmol, 3.0 mol %), PPh_3 (0.79 mg, 0.0030 mmol, 3.0 mol %), $B_2(dan)_2$ (**2**, 37 mg, 0.11 mmol, 1.1 equiv) and allene **1** (0.10 mmol, 1.0 equiv) in toluene (1.0 mL) was stirred for 30 min at 110 °C. The resulting mixture was concentrated under reduced pressure and the residue was purified by silica gel column chromatography (hexane/AcOEt) to afford **3**.

V. Characterization Data

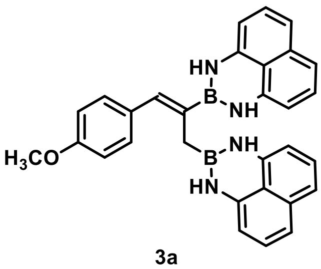

3a

(*Z*)-2,2'-(3-(4-Methoxyphenyl)prop-2-ene-1,2-diyl)bis(2,3-dihydro-1H-naphtho[1,8-de][1,3,2]-diazaborinine) (**3a**): **3a** (48 mg, 0.093 mmol, 93%) was synthesized from **1a** (15 mg, 0.10 mmol). Purified by silica gel column chromatography (hexane/AcOEt = 10/1). White amorphous solid. ^{1}H NMR ($CDCl_3$, 400 MHz): δ 7.32 (d, J = 8.8 Hz, 2H), 7.12-7.07 (m, 4H), 7.04-7.01 (m, 5H), 6.89 (d, J = 8.8 Hz, 2H), 6.36 (dd, J = 7.3, 0.9 Hz, 2H), 6.29 (dd, J = 7.1, 1.1 Hz, 2H), 5.86 (s, 2H), 5.70 (s, 2H), 3.80 (s, 3H), 2.21 (s, 2H); ^{13}C{^{1}H} NMR ($CDCl_3$, 100 MHz): δ 158.9, 141.2, 140.9, 136.5, 136.45, 136.39, 130.6, 130.4, 127.82, 127.77, 120.0, 119.9, 118.1, 118.0, 114.1, 106.3, 106.2, 55.5, 18.4, the signal of the boron-bound carbon (sp^2) was obscure due to the quadrupolar boron nucleus; ^{11}B{^{1}H} NMR ($CDCl_3$, 128 MHz): δ 31.0; IR (ATR): 3426, 3401, 1630, 1605, 1509, 1411, 770 cm^{-1}; HRMS (ESI/Q-TOF) *m/z*: $[M + H]^+$ calcd for $C_{30}H_{27}{}^{10}B_2N_4O$ 479.2438. Found 479.2440.

10 mmol scale synthesis of 3a: A mixture of $Pt(dba)_3$ (90 mg, 0.10 mmol, 1.0 mol %), PPh_3 (26 mg, 0.10 mmol, 1.0 mol %), $B_2(dan)_2$ (**2**, 3.7 mg, 11 mmol, 1.1 equiv) and **1a** (1.5 g, 10 mmol, 1.0 equiv) in toluene (100 mL) was stirred for 1 h at 110 °C. The resulting mixture was concentrated under reduced pressure and the residue was purified by silica gel column chromatography (hexane/AcOEt) to afford **3a** (4.2 g, 8.8 mmol, 88%).

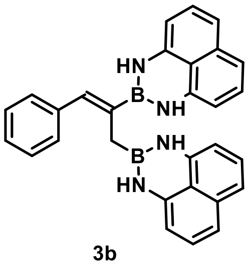

3b

(*Z*)-2,2'-(3-Phenylprop-2-ene-1,2-diyl)bis(2,3-dihydro-1H-naphtho[1,8-de][1,3,2]diazaborinine) (**3b**): **3b** (32 mg, 0.070 mmol, 70%) was synthesized from **1b** (12 mg, 0.10 mmol). Purified by silica gel column chromatography (hexane/AcOEt = 10/1). White amorphous solid. ^{1}H NMR ($CDCl_3$, 400 MHz): δ 7.35 (d, J = 4.6 Hz, 4H), 7.27-7.24 (m, 1H), 7.12-7.06 (m, 5H), 7.02 (d, J = 7.6 Hz, 4H), 6.36 (dd, J = 7.3, 0.9 Hz, 2H), 6.29 (dd, J = 7.3, 0.9 Hz, 2H), 5.86 (s, 2H), 5.68 (s, 2H), 2.21 (s, 2H); ^{13}C{^{1}H} NMR ($CDCl_3$, 100 MHz): δ 141.1, 140.9, 137.8, 136.8, 136.5, 136.4, 129.1, 128.7, 127.81, 127.77, 127.5, 120.0, 119.9, 118.1, 118.0, 106.3, 106.2, the signals of the boron-bound carbon atom were obscure due to the quadrupolar boron nucleus; ^{11}B{^{1}H} NMR ($CDCl_3$, 128 MHz): δ 31.9; IR (ATR): 3430, 3419, 3397, 3377, 3047, 1627, 1598, 1504, 1405, 757 cm^{-1}; HRMS (ESI/Q-TOF) *m/z*: $[M + H]^+$ calcd for $C_{29}H_{25}{}^{10}B_2N_4$ 449.2333. Found 449.2335.

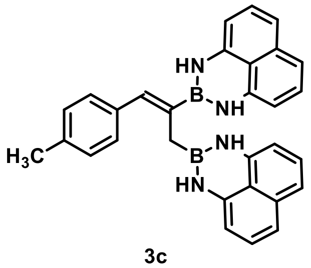

3c

(*Z*)-2,2'-(3-(*p*-Tolyl)prop-2-ene-1,2-diyl)bis(2,3-dihydro-1H-naphtho[1,8-de][1,3,2]diazaborinine) (**3c**): **3c** (38 mg, 0.081 mmol, 81%) was synthesized from **1c** (13 mg, 0.10 mmol). Purified by silica gel column chromatography (hexane/AcOEt = 10/1). White amorphous solid. ^{1}H NMR ($CDCl_3$, 400 MHz): δ 7.26 (d, J = 8.0 Hz, 2H), 7.16 (d, J = 7.8 Hz, 2H), 7.12-7.06 (m, 5H), 7.02 (d, J = 8.2 Hz, 4H), 6.36 (d, J = 7.3 Hz, 2H), 6.29 (dd, J = 7.3, 0.9 Hz, 2H), 5.86 (s, 2H), 5.68 (s, 2H), 2.34 (s, 3H), 2.21 (s, 2H); ^{13}C{^{1}H} NMR ($CDCl_3$, 100 MHz): δ 141.2, 140.9, 137.4, 136.8, 136.51, 136.46, 135.0, 129.4, 129.1, 127.81, 127.77, 120.0, 119.9, 118.1, 118.0, 106.3, 106.2, 21.4, 18.6, the signal of the boron-bound carbon (*sp*^{2}) was obscure due to the quadrupolar boron nucleus ; ^{11}B{^{1}H} NMR ($CDCl_3$, 128 MHz): δ 30.4; IR (ATR): 3426, 3053, 2878, 1629, 1607, 1510, 1412, 1337, 817, 776, 761 cm^{-1}; HRMS (ESI/Q-TOF) *m/z*: [M + H]$^+$ calcd for $C_{30}H_{27}{}^{10}B_2N_4$ 463.2489. Found 463.2489.

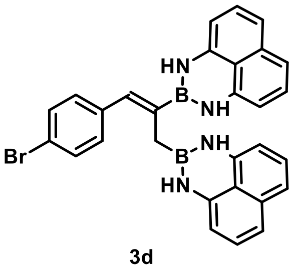

3d

(*Z*)-2,2'-(3-(4-Bromophenyl)prop-2-ene-1,2-diyl)bis(2,3-dihydro-1H-naphtho[1,8-de][1,3,2]-diazaborinine) (**3d**): **3d** (48 mg, 0.091 mmol, 91%) was synthesized from **1d** (20 mg, 0.10 mmol). Purified by silica gel column chromatography (hexane/AcOEt = 10/1). White solid. Mp: 183.0-184.4 °C; ^{1}H NMR ($CDCl_3$, 400 MHz): δ 7.47 (dt, J = 8.8, 2.3 Hz, 2H), 7.21 (dt, J = 8.8, 2.0 Hz, 2H), 7.12-7.06 (m, 4H), 7.03-7.01 (m, 5H), 6.36 (dd, J = 7.3, 1.0 Hz, 2H), 6.29 (dd, J = 7.1, 1.1 Hz, 2H), 5.85 (s, 2H), 5.65 (s, 2H), 2.17 (s, 2H); ^{13}C{^{1}H} NMR ($CDCl_3$, 100 MHz): δ 141.0, 140.7, 136.7, 136.5, 136.4, 135.5, 131.8, 130.7, 127.82, 127.77, 121.4, 120.0, 119.9, 118.3, 118.2, 106.4, 106.3, the signals of the boron-bound carbon atom were obscure due to the quadrupolar boron nucleus; ^{11}B{^{1}H} NMR ($CDCl_3$, 128 MHz): δ 30.7; IR (ATR): 3413, 3391, 3050, 2884, 1597, 1498, 1407 cm^{-1}; HRMS (ESI/Q-TOF) *m/z*: [M + H]$^+$ calcd for $C_{29}H_{24}{}^{10}B_2{}^{79}BrN_4$ 527.1438. Found 527.1441.

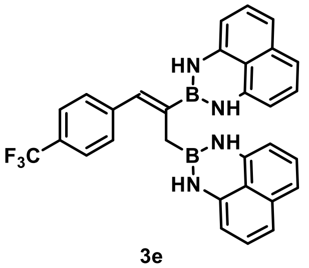

3e

(*Z*)-2,2'-(3-(4-(Trifluoromethyl)phenyl)prop-2-ene-1,2-diyl)bis(2,3-dihydro-1H-naphtho[1,8-de][1,3,2]-diazaborinine) (3e): **3e** (48 mg, 0.093 mmol, 93%) was synthesized from **1e** (18 mg, 0.10 mmol). Purified by silica gel column chromatography (hexane/AcOEt = 10/1). Yellow solid. Mp: 182.7 °C (decomp); ^{1}H NMR ($CDCl_3$, 400 MHz): δ 7.61 (d, J = 8.3 Hz, 2H), 7.44 (d, J = 8.5 Hz, 2H), 7.13-7.00 (m, 9H), 6.37 (dd, J = 7.3, 1.3 Hz, 2H), 6.30 (dd, J = 7.1, 1.1 Hz, 2H), 5.87 (s, 2H), 5.65 (s, 2H), 2.19 (s, 2H); ^{13}C{^{1}H} NMR ($CDCl_3$, 100 MHz): δ 141.4, 140.9, 140.7, 137.9, 136.5, 136.4, 135.2, 129.28 (q, $J_{F,C}$ = 31.8 Hz), 129.27, 127.84, 127.78, 125.6 (q, $J_{F,C}$ = 3.6 Hz), 124.3 (q, $J_{F,C}$ = 270 Hz), 120.1, 119.9, 118.33, 118.27, 106.5, 106.3, 18.7; ^{11}B{^{1}H} NMR ($CDCl_3$, 128 MHz): δ 32.3; IR (ATR): 3454, 3419, 3393, 3053, 2883, 1628, 1603, 1507, 1411, 1372, 1326, 1127, 821, 770 cm^{-1}; HRMS (ESI/Q-TOF) *m/z*: $[M + H]^+$ calcd for $C_{30}H_{24}{}^{10}B_2F_3N_4$ 517.2206. Found 517.2201.

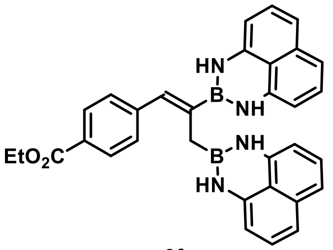

3f

Ethyl (*Z*)-4-(2,3-bis(1H-naphtho[1,8-de][1,3,2]diazaborinin-2(3H)-yl)prop-1-en-1-yl)benzoate (3f): **3f** (39 mg, 0.074 mmol, 74%) was synthesized from **1f** (19 mg, 0.10 mmol) for 1 h at 110 ºC. Purified by silica gel column chromatography (hexane/AcOEt = 5/1). Yellow solid. Mp: 178.5 °C (decomp); ^{1}H NMR ($CDCl_3$, 400 MHz): δ 8.03 (dt, J = 8.4, 1.6 Hz, 2H), 7.40 (d, J = 8.0 Hz, 2H), 7.12-7.01 (m, 9H), 6.37 (dd, J = 7.2, 1.0 Hz, 2H), 6.29 (dd, J = 7.3, 1.3 Hz, 2H), 5.88 (s, 2H), 5.66 (s, 2H), 4.35 (q, J = 7.2 Hz, 2H), 2.20 (s, 2H), 1.37 (t, J = 7.1 Hz, 3H); ^{13}C{^{1}H} NMR ($CDCl_3$, 100 MHz): δ 166.5, 142.3, 140.9, 140.7, 136.5, 136.4, 135.7, 129.9, 129.2, 129.0, 127.82, 127.75, 120.1, 119.9, 118.3, 118.2, 106.4, 106.3, 61.2, 18.8, the signals of the boron-bound carbon were obscure due to the quadrupolar boron nucleus; ^{11}B{^{1}H} NMR ($CDCl_3$, 128 MHz): δ 28.9; IR (ATR): 3430, 3389, 3363, 1599, 1406 cm^{-1}; HRMS (ESI/Q-TOF) *m/z*: $[M + H]^+$ calcd for $C_{32}H_{29}{}^{10}B_2N_4O_2$ 521.2544. Found 521.2543.

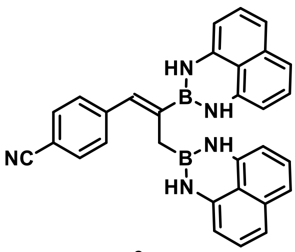

3g

(*Z*)-4-(2,3-Bis(1H-naphtho[1,8-de][1,3,2]diazaborinin-2(3H)-yl)prop-1-en-1-yl)benzonitrile (**3g**): **3g** (28 mg, 0.060 mmol, 60%) was synthesized from **1g** (14 mg, 0.10 mmol). Purified by silica gel column chromatography (hexane/AcOEt = 5/1). Yellow solid. Mp: 209.8 °C (decomp); ^{1}H NMR (DMSO-d_6, 400 MHz): δ 7.95 (s, 2H), 7.86 (d, *J* = 8.3 Hz, 2H), 7.58 (d, *J* = 8.3 Hz, 2H), 7.46 (s, 2H), 7.21 (s, 1H), 7.08-7.01 (m, 4H), 6.87 (t, *J* = 7.5 Hz, 4H), 6.53 (d, *J* = 6.8 Hz, 2H), 6.45 (d, *J* = 6.8 Hz, 2H), 2.28 (s, 2H); ^{13}C{^{1}H} NMR (DMSO-d_6, 100 MHz): δ 143.1, 142.3, 141.9, 140.5, 135.93, 135.86, 134.1, 132.2, 129.5, 127.6, 127.5, 119.7, 119.3, 119.0, 116.22, 116.19, 108.9, 105.6, 105.4, 18.6; ^{11}B{^{1}H} NMR (DMSO-d_6, 128 MHz): δ 32.4; IR (ATR): 3389, 3364, 2220, 1596, 1498, 1406, 820, 768 cm^{-1}; HRMS (ESI/Q-TOF) *m/z*: [M + H]$^{+}$ calcd for $C_{30}H_{24}{}^{10}B_2N_5$ 474.2285. Found 474.2283.

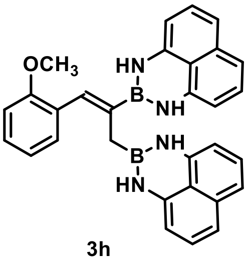

3h

(*Z*)-2,2'-(3-(2-Methoxyphenyl)prop-2-ene-1,2-diyl)bis(2,3-dihydro-1H-naphtho[1,8-de][1,3,2]-diazaborinine) (**3h**): **3h** (40 mg, 0.082 mmol, 82%) was synthesized from **1h** (15 mg, 0.10 mmol). Purified by silica gel column chromatography (hexane/AcOEt = 10/1). White amorphous solid. ^{1}H NMR (CDCl$_3$, 400 MHz): δ 7.27-7.22 (m, 2H), 7.14 (s, 1H), 7.11-7.06 (m, 4H), 7.02-6.98 (m, 4H), 6.96-6.89 (m, 2H), 6.34 (dd, *J* = 7.3, 1.0 Hz, 2H), 6.27 (dd, *J* = 7.3, 0.8 Hz, 2H), 5.88 (s, 2H), 5.72 (s, 2H), 3.87 (s, 3H), 2.07 (s, 2H); ^{13}C{^{1}H} NMR (CDCl$_3$, 100 MHz): δ 157.1, 141.2, 141.1, 136.5, 136.4, 133.2, 130.0, 128.9, 127.8, 127.7, 127.0, 120.6, 120.0, 119.8, 117.88, 117.86, 110.8, 106.2, 106.0, 55.7, 18.4, the signal of the boron-bound carbon (*sp^2*) was obscure due to the quadrupolar boron nucleus; ^{11}B{^{1}H} NMR (CDCl$_3$, 128 MHz): δ 30.7; IR (ATR): 3426, 3401, 3384, 3055, 1620, 1505, 1427, 817, 762 cm^{-1}; HRMS (ESI/Q-TOF) *m/z*: [M + H]$^{+}$ calcd for $C_{30}H_{27}{}^{10}B_2N_4O$ 479.2438. Found 479.2434.

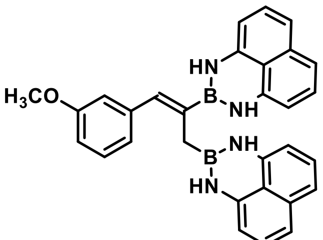

3i

(*Z*)-2,2'-(3-(3-Methoxyphenyl)prop-2-ene-1,2-diyl)bis(2,3-dihydro-1H-naphtho[1,8-de][1,3,2]-diazaborinine) (**3i**): **3i** (40 mg, 0.084 mmol, 84%) was synthesized from **1i** (15 mg, 0.10 mmol). Purified by silica gel column chromatography (hexane/AcOEt = 10/1). White amorphous solid. ^{1}H NMR ($CDCl_3$, 400 MHz): δ 7.27 (t, *J* = 8.0 Hz, 1H), 7.12-7.06 (m, 5H), 7.02 (ddd, *J* = 8.3, 3.2, 0.9 Hz, 4H), 6.95 (d, *J* = 7.8 Hz, 1H), 6.91 (t, *J* = 1.9 Hz, 1H), 6.81 (dd, *J* = 8.0, 2.3 Hz, 1H), 6.36 (dd, *J* = 7.3, 1.0 Hz, 2H), 6.29 (dd, *J* = 7.3, 1.0 Hz, 2H), 5.87 (s, 2H), 5.67 (s, 2H), 3.76 (s, 3H), 2.21 (s, 2H); ^{13}C{^{1}H} NMR ($CDCl_3$, 100 MHz): δ 159.8, 141.1, 140.8, 139.3, 136.6, 136.5, 136.4, 129.7, 127.82, 127.76, 121.5, 120.0, 119.8, 118.12, 118.06, 114.5, 113.2, 106.3, 106.2, 55.4, 18.7, the signal of the boron-bound carbon (*sp*^{2}) was obscure due to the quadrupolar boron nucleus; ^{11}B{^{1}H} NMR ($CDCl_3$, 128 MHz): δ 31.0; IR (ATR): 3438, 3401, 3361, 3049, 1599, 1506, 1410 cm^{-1}; HRMS (ESI/Q-TOF) *m/z*: $[M + H]^+$ calcd for $C_{30}H_{27}{}^{10}B_2N_4O$ 479.2438. Found 479.2438.

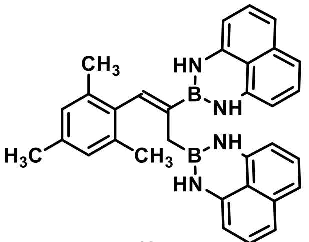

3j

(*Z*)-2,2'-(3-Mesitylprop-2-ene-1,2-diyl)bis(2,3-dihydro-1H-naphtho[1,8-de][1,3,2]diazaborinine) (**3j**): **3j** (42 mg, 0.086 mmol, 86%) was synthesized from **1j** (16 mg, 0.10 mmol). Purified by silica gel column chromatography (hexane/AcOEt = 10/1). White amorphous solid. ^{1}H NMR ($CDCl_3$, 400 MHz): δ 7.13-6.89 (m, 11H), 6.37 (dd, *J* = 7.3, 0.9 Hz, 2H), 6.19 (dd, *J* = 7.3, 0.9 Hz, 2H), 5.89 (s, 2H), 5.41 (s, 2H), 2.29 (s, 3H), 2.20 (s, 6H), 1.75 (s, 2H); ^{13}C{^{1}H} NMR ($CDCl_3$, 100 MHz): δ 141.1, 140.9, 136.7, 136.6, 136.4, 135.6, 134.4, 128.5, 127.8, 127.7, 120.0, 119.7, 118.1, 117.9, 106.3, 105.9, 21.2, 20.5, 18.5, the signal of the boron-bound carbon (*sp*^{2}) was obscure due to the quadrupolar boron nucleus and one signal was missing; ^{11}B{^{1}H} NMR ($CDCl_3$, 128 MHz): δ 31.0; IR (ATR): 3420, 3051, 2914, 1628, 1594, 1508, 1409, 1373, 1337, 820, 765 cm^{-1}; HRMS (ESI/Q-TOF) *m/z*: $[M + H]^+$ calcd for $C_{32}H_{31}{}^{10}B_2N_4$ 491.2802. Found 491.2800.

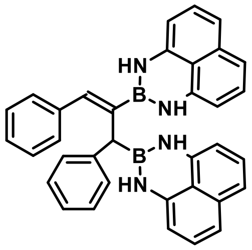

3k

(*Z*)-2,2'-(1,3-Diphenylprop-2-ene-1,2-diyl)bis(2,3-dihydro-1H-naphtho[1,8-de][1,3,2]diazaborinine) (**3k**): **3k** (30 mg, 0.057 mmol, 57%) was synthesized from **1k** (19 mg, 0.10 mmol). Purified by silica gel column chromatography (hexane/AcOEt = 10/1). Yellow amorphous solid. ^{1}H NMR ($CDCl_3$, 400 MHz): δ 7.44-7.42 (m, 2H), 7.38-7.33 (m, 4H), 7.28-7.24 (m, 3H), 7.21-7.18 (m, 1H), 7.11-6.96 (m, 9H), 6.27 (dd, *J* = 7.0, 1.0 Hz, 2H), 6.04 (dd, *J* = 6.8, 1.2 Hz, 2H), 5.65 (s, 2H), 5.42 (s, 2H), 3.60 (s, 1H); ^{13}C{^{1}H} NMR ($CDCl_3$, 100 MHz): δ 142.0, 140.82, 140.80, 139.9, 138.3, 136.5, 136.4, 129.5, 129.3, 128.7, 128.5, 127.8, 127.7, 127.6, 126.5, 119.83, 119.78, 118.2, 117.9, 106.34, 106.28, 45.7, the signal of the boron-bound carbon (*sp*^{2}) was obscure due to the quadrupolar boron nucleus; ^{11}B{^{1}H} NMR ($CDCl_3$, 128 MHz): δ 31.9; IR (ATR): 3416, 3053, 1599, 1498, 1410, 1374, 820, 765 cm^{-1}; HRMS (ESI/Q-TOF) *m/z*: $[M + H]^+$ calcd for $C_{35}H_{29}{}^{10}B_2N_4$ 525.2646. Found 525.2646.

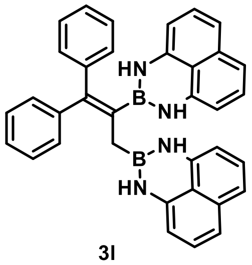

3l

2,2'-(3,3-Diphenylprop-2-ene-1,2-diyl)bis(2,3-dihydro-1H-naphtho[1,8-de][1,3,2]diazaborinine) (**3l**): **3l** (49 mg, 0.093 mmol, 93%) was synthesized from **1l** (19 mg, 0.10 mmol). Purified by silica gel column chromatography (hexane/AcOEt =10/1). Yellow amorphous solid. ^{1}H NMR ($CDCl_3$, 400 MHz): δ 7.41 (t, *J* = 7.0 Hz, 2H), 7.34-7.24 (m, 8H), 7.14-7.02 (m, 8H), 6.28 (d, *J* = 7.3 Hz, 2H), 6.12 (d, *J* = 7.0 Hz, 2H), 5.64 (s, 2H), 5.48 (s, 2H), 2.07 (s, 2H); ^{13}C{^{1}H} NMR ($CDCl_3$, 100 MHz): δ 150.3, 143.9, 143.0, 141.0, 140.9, 136.4, 136.3, 133.3, 129.5, 129.4, 128.7, 128.4, 127.7, 127.6, 127.2, 119.7, 119.6, 117.9, 117.8, 22.6, three signals were missing; ^{11}B{^{1}H} NMR ($CDCl_3$, 128 MHz): δ 31.3; IR (ATR): 3421, 3053, 1632, 1607, 1507, 1413, 821, 768 cm^{-1}; HRMS (ESI/Q-TOF) *m/z*: $[M + H]^+$ calcd for $C_{35}H_{29}{}^{10}B_2N_4$ 525.2645. Found 525.2641.

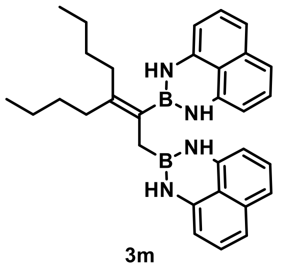

3m

2,2'-(3-Butylhept-2-ene-1,2-diyl)bis(2,3-dihydro-1H-naphtho[1,8-de][1,3,2]diazaborinine) (**3m**): **3m** (45 mg, 0.092 mmol, 92%) was synthesized from **1m** (15 mg, 0.10 mmol). Purified by silica gel column chromatography (hexane/AcOEt = 10/1). Colorless oil. ^{1}H NMR ($CDCl_3$, 400 MHz): δ 7.14-7.01 (m, 8H), 6.30-6.27 (m, 4H), 5.66 (s, 2H), 5.59 (s, 2H), 2.27 (t, *J* = 7.5 Hz, 2H), 2.12 (t, *J* = 7.8 Hz, 2H), 1.88 (s, 2H), 1.51-1.34 (m, 8H), 0.96 (td, *J* = 7.1, 2.0 Hz, 6H); ^{13}C{^{1}H} NMR ($CDCl_3$, 100 MHz): δ 146.7, 141.3, 141.1, 136.5, 136.4, 127.79, 127.77, 119.8, 119.7, 117.9, 117.7, 106.0, 105.9, 36.4, 32.4, 30.9, 30.6, 23.4, 23.0, 19.6, 14.3, the signal of the boron-bound carbon (*sp*^{2}) was obscure due to the quadrupolar boron nucleus and one signal was missing; ^{11}B{^{1}H} NMR ($CDCl_3$, 128 MHz): δ 31.8; IR (ATR): 3409, 3052, 2953, 2927, 2856, 1602, 1505, 1409, 820, 778 cm^{-1}; HRMS (ESI/Q-TOF) *m/z*: [M + H]$^+$ calcd for $C_{31}H_{37}{}^{10}B_2N_4$ 485.3271. Found 485.3272.

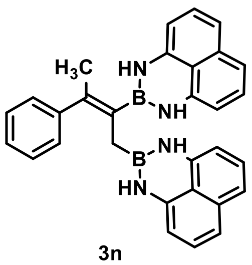

3n

(*Z*)-2,2'-(3-Phenylbut-2-ene-1,2-diyl)bis(2,3-dihydro-1H-naphtho[1,8-de][1,3,2]diazaborinine) (**3n**): **3n** (40 mg, 0.087 mmol, 87%) was synthesized from **1n** (13 mg, 0.10 mmol). Purified by silica gel column chromatography (hexane/AcOEt = 10/1). White amorphous solid. ^{1}H NMR ($CDCl_3$, 400 MHz): δ 7.35-7.27 (m, 5H), 7.15-7.09 (m, 2H), 7.03 (dd, *J* = 8.4, 0.9 Hz, 2H), 7.00-6.97 (m, 2H), 6.92 (dd, *J* = 8.3, 1.0 Hz, 2H), 6.32 (dd, *J* = 7.2, 0.9 Hz, 2H), 5.99 (dd, *J* = 7.3, 1.0 Hz, 2H), 5.70 (s, 2H), 5.18 (s, 2H), 2.14 (s, 3H), 2.07 (s, 2H); ^{13}C{^{1}H} NMR ($CDCl_3$, 100 MHz): δ 146.1, 145.8, 141.2, 141.0, 136.5, 136.3, 128.6, 128.0, 127.8, 127.7, 127.4, 119.9, 119.4, 118.1, 117.5, 106.1, 105.8, 21.8, 20.7, the signal of the boron-bound carbon (*sp*^{2}) was obscure due to the quadrupolar boron nucleus; ^{11}B{^{1}H} NMR ($CDCl_3$, 128 MHz): δ 31.3; IR (ATR): 3424, 3052, 2903, 1630, 1506, 1412, 821, 768 cm^{-1}; HRMS (ESI/Q-TOF) *m/z*: [M + H]$^+$ calcd for $C_{30}H_{27}{}^{10}B_2N_4$ 463.2489. Found 463.2490.

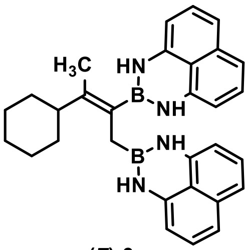

(*Z*)-3o

(*Z*)-2,2'-(3-Cyclohexylbut-2-ene-1,2-diyl)bis(2,3-dihydro-1H-naphtho[1,8-de][1,3,2]diazaborinine) (**(*Z*)-3o**): **(*Z*)-3o** (21 mg, 0.045 mmol, 45%) was synthesized from **1o** (14 mg, 0.10 mmol). Purified by silica gel column chromatography (hexane/AcOEt = 10/1 and hexane/MTBE = 5/1). White amorphous solid. ^{1}H NMR ($CDCl_3$, 400 MHz): δ 7.11-6.98 (m, 8H), 6.30-6.26 (m, 4H), 5.63 (s, 2H), 5.58 (s, 2H), 2.49-2.44 (m, 1H), 1.87 (s, 2H), 1.82-1.67 (m, 6H), 1.52-1.15 (m, 7H); ^{13}C{^{1}H} NMR ($CDCl_3$, 100 MHz): δ 145.9, 141.4, 141.2, 136.52, 136.48, 127.82, 127.78, 119.8, 119.7, 117.9, 117.7, 106.0, 105.9, 41.3, 31.2, 26.9, 26.4, 19.2, 18.8, the signal of the boron-bound carbon (*sp*^{2}) was obscure due to the quadrupolar boron nucleus; ^{11}B{^{1}H} NMR ($CDCl_3$, 128 MHz): δ 31.4; IR (ATR): 3403, 3051, 2924, 2848, 1598, 1498, 1408, 764 cm^{-1}; HRMS (ESI/Q-TOF) *m/z*: [M + H]$^+$ calcd for $C_{30}H_{32}{}^{10}B_2N_4$ 469.2958. Found 469.2961.

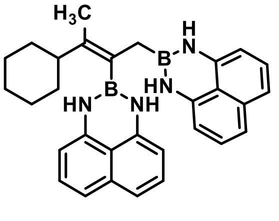

(*E*)-3o

(*E*)-2,2'-(3-cyclohexylbut-2-ene-1,2-diyl)bis(2,3-dihydro-1H-naphtho[1,8-de][1,3,2]diazaborinine) (**(*E*)-3o**): **(*E*)-3o** (17 mg, 0.036 mmol, 36%) was synthesized from **1o** (14 mg, 0.10 mmol). Purified by silica gel column chromatography (hexane/AcOEt = 10/1 and hexane/MTBE = 5/1). White amorphous solid. ^{1}H NMR ($CDCl_3$, 400 MHz): δ 7.11-7.05 (m, 4H), 7.02-6.98 (m, 4H), 6.27 (d, J = 7.3 Hz, 4H), 5.62 (s, 2H), 5.53 (s, 2H), 2.45-2.39 (m, 1H), 1.82 (s, 2H), 1.77-1.73 (m, 2H), 1.67-1.62 (m, 4H), 1.56-1.43 (m, 4H), 1.33-1.23 (m, 2H), 1.19-1.12 (m, 1H); ^{13}C{^{1}H} NMR ($CDCl_3$, 100 MHz): δ 147.3, 141.4, 141.2, 136.53, 136.46, 127.8, 119.83, 119.76, 117.9, 117.8, 106.1, 105.9, 48.1, 32.6, 26.5, 26.2, 19.7, 13.7, one signal was missing and the signal of the boron-bound carbon (*sp*^{2}) was obscure due to the quadrupolar boron nucleus; ^{11}B{^{1}H} NMR ($CDCl_3$, 128 MHz): δ 31.5; IR (ATR): 3409, 3053, 2926, 2851, 1629, 1605, 1505, 1411, 766 cm^{-1}; HRMS (ESI/Q-TOF) *m/z*: [M + H]$^+$ calcd for $C_{30}H_{32}{}^{10}B_2N_4$ 469.2958. Found 469.2959.

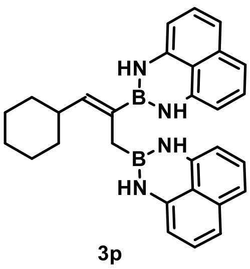

3p

(*Z*)-2,2'-(3-Cyclohexylprop-2-ene-1,2-diyl)bis(2,3-dihydro-1H-naphtho[1,8-de][1,3,2]diazaborinine) (**3p**): **3p** (40 mg, 0.087 mmol, 87%) was synthesized from **1p** (12 mg, 0.10 mmol). Purified by silica gel column chromatography (hexane/AcOEt = 10/1). Colorless oil. ^{1}H NMR ($CDCl_3$, 400 MHz): δ 7.09 (td, *J* = 8.0, 2.0 Hz, 4H), 7.02 (t, *J* = 7.8 Hz, 4H), 6.34-6.24 (m, 4H), 5.95 (d, *J* = 9.3 Hz, 1H), 5.73 (s, 2H), 5.62 (s, 2H), 2.39-2.31 (m, 1H), 1.94 (s, 2H), 1.76-1.66 (m, 5H), 1.35-1.11 (m, 5H); ^{13}C{^{1}H} NMR ($CDCl_3$, 100 MHz): δ 145.6, 141.3, 141.0, 136.5, 129.8, 127.8, 119.8, 117.9, 117.7, 106.05, 105.98, 37.9, 33.0, 26.2, 26.1, 17.0, three signals were missing; ^{11}B{^{1}H} NMR ($CDCl_3$, 128 MHz): δ 30.8; IR (ATR): 3421, 3052, 2926, 2848, 1629, 1606, 1506, 1412, 910, 821, 767, 736 cm^{-1}; HRMS (ESI/Q-TOF) *m/z*: [M + H]$^{+}$ calcd for $C_{29}H_{31}{}^{10}B_2N_4$ 455.2802. Found 455.2803.

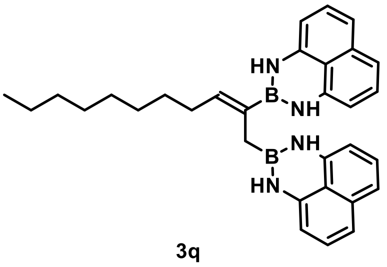

3q

(*Z*)-2,2'-(Undec-2-ene-1,2-diyl)bis(2,3-dihydro-1H-naphtho[1,8-de][1,3,2]diazaborinine) (**3q**): **3q** (11mg, 0.023 mmol, 23%) was synthesized from **1q** (15 mg, 0.10 mmol). Purified by silica gel column chromatography (hexane/AcOEt = 20/1). Colorless oil. ^{1}H NMR ($CDCl_3$, 400 MHz): δ 7.12 (td, *J* = 7.6, 2.0 Hz, 4H), 7.04 (dd, *J* = 8.3, 3.0 Hz, 4H), 6.35 (dd, *J* = 7.6, 0.8 Hz, 2H), 6.31 (dd, *J* = 7.3, 1.0 Hz, 2H), 6.15 (t, *J* = 6.8 Hz, 1H), 5.75 (s, 2H), 5.62 (s, 2H), 2.18 (q, *J* = 7.2 Hz, 2H), 1.93 (s, 2H), 1.51-1.29 (m, 12H), 0.90 (t, *J* = 6.9 Hz, 3H); ^{13}C{^{1}H} NMR ($CDCl_3$, 100 MHz): δ 141.3, 141.0, 140.1, 136.5, 132.1, 127.8, 127.7, 119.8, 117.9, 117.7, 106.1, 106.0, 32.1, 29.80, 29.78, 29.6, 29.5, 29.1, 22.9, 17.0, 14.3, two signals were missing; ^{11}B{^{1}H} NMR ($CDCl_3$, 128 MHz): δ 30.0; IR (ATR): 3419, 3052, 2926, 2852, 1629, 1606, 1506, 1412, 820, 768 cm^{-1}; HRMS (ESI/Q-TOF) *m/z*: [M + H]$^{+}$ calcd for $C_{31}H_{37}{}^{10}B_2N_4$ 485.3271. Found 485.3272.

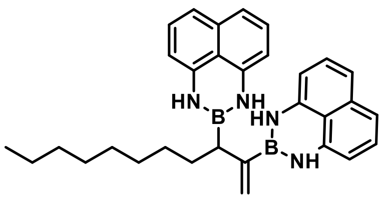

3q'

2,2'-(Undec-1-ene-2,3-diyl)bis(2,3-dihydro-1H-naphtho[1,8-de][1,3,2]diazaborinine) (**3q'**): **3q'** (29 mg, 0.059 mmol, 59%) was synthesized from **1q** (15 mg, 0.10 mmol). Purified by silica gel column chromatography (hexane/AcOEt = 10/1). Colorless oil. ^{1}H NMR (CDCl$_3$, 400 MHz): δ 7.20-7.04 (m, 8H), 6.39 (d, *J* = 7.0 Hz, 2H), 6.30 (d, *J* = 7.2 Hz, 2H), 5.81 (s, 2H), 5.71 (s, 2H), 5.64 (d, *J* = 2.5 Hz, 1H), 5.57 (s, 1H), 2.03 (t, *J* = 7.8 Hz, 1H), 1.70-1.68 (m, 2H), 1.48-1.34 (m, 12H), 0.95 (t, *J* = 5.9 Hz, 3H); ^{13}C{^{1}H} NMR (CDCl$_3$, 100 MHz): δ 149.1, 141.0, 140.9, 136.45, 136.41, 127.8, 127.7, 122.0, 120.0, 119.8, 118.1, 117.9, 106.2, 35.5, 32.1, 30.2, 29.9, 29.7, 29.6, 29.5, 22.9, 14.3, one signal was missing; ^{11}B{^{1}H} NMR (CDCl$_3$, 128 MHz): δ 30.1; IR (ATR): 3419, 3052, 2924, 2852, 1630, 1607, 1506, 1411, 820, 774, 768 cm^{-1}; HRMS (ESI/Q-TOF) *m/z*: [M + H]$^{+}$ calcd for $C_{31}H_{37}{}^{10}B_2N_4$ 485.3271. Found 485.3271.

VI. ^{1}H-^{1}H NOESY analysis of 3a, 3n, (*Z*)-3o, (*E*)-3o, 3p and 3q

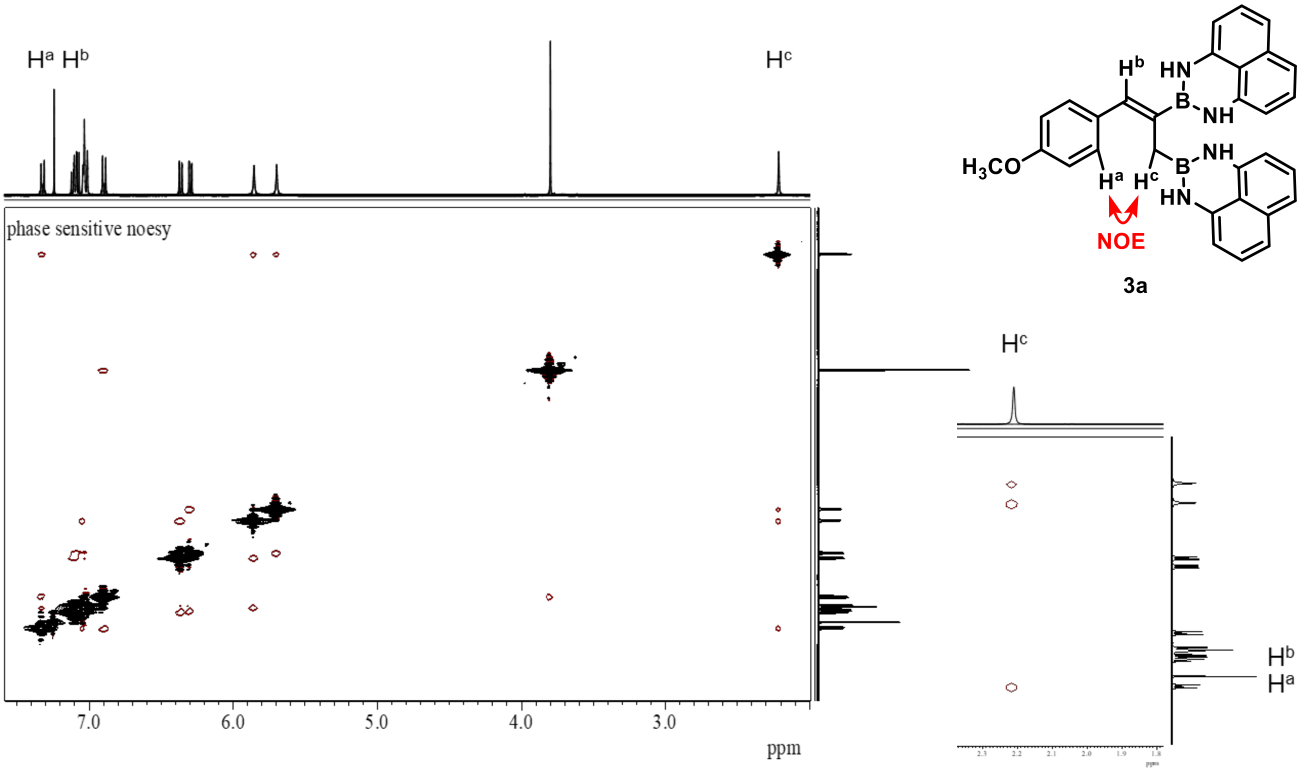

Figure S1. ^{1}H-^{1}H NOESY (CDCl$_3$, 400 MHz) spectrum of **3a**

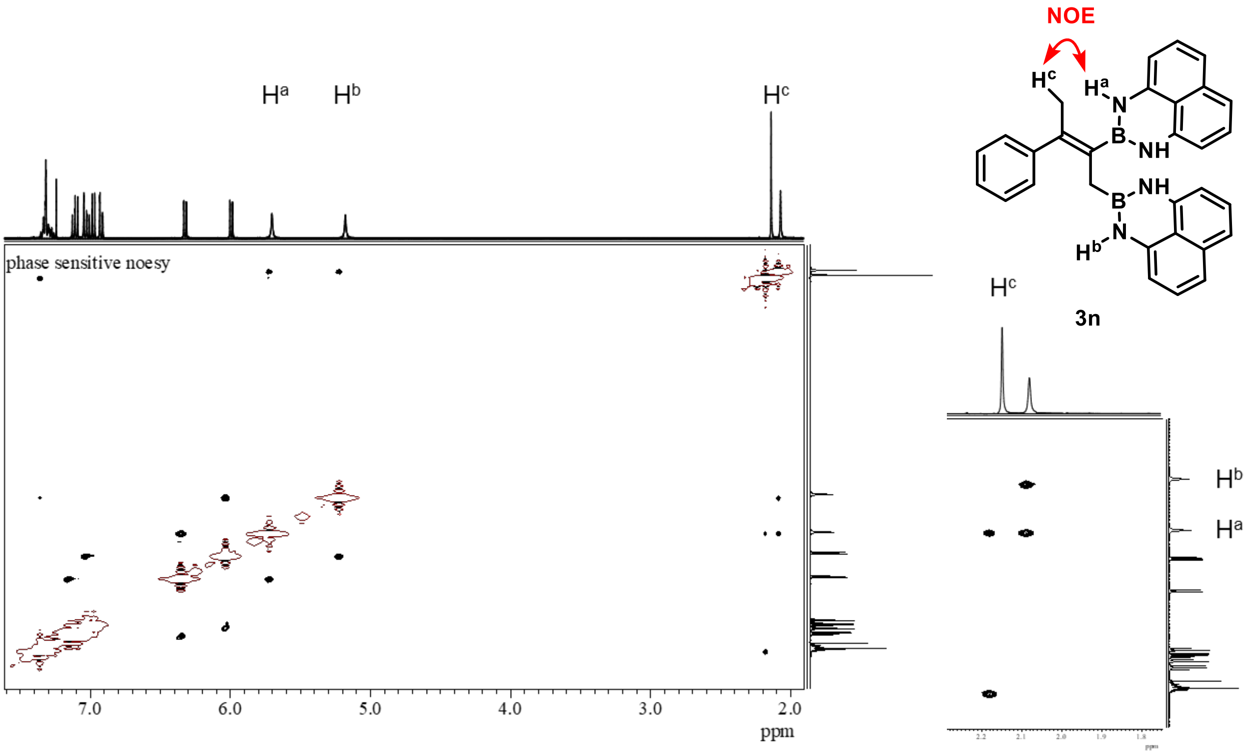

Figure S2. ^{1}H-^{1}H NOESY (CDCl$_3$, 400 MHz) spectrum of **3n**

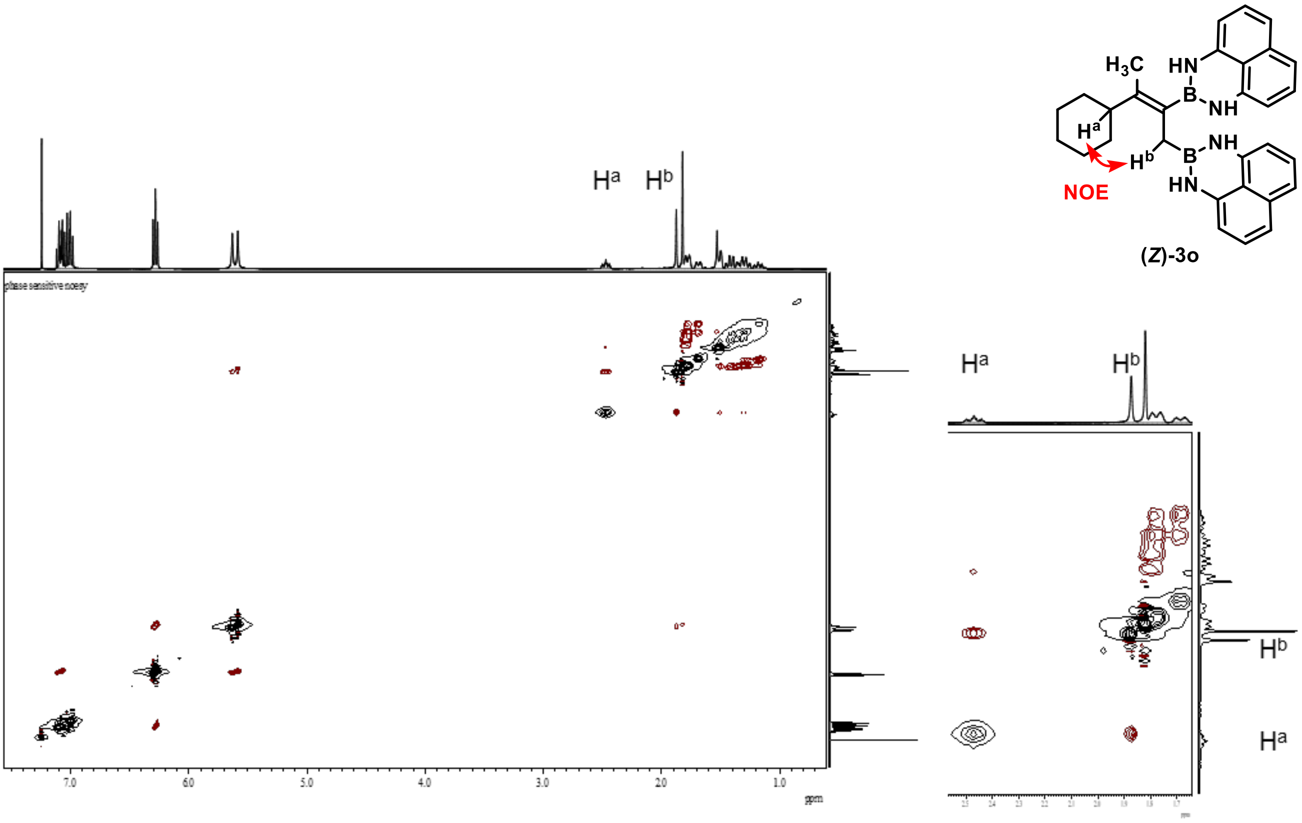

Figure S3. ^{1}H-^{1}H NOESY (CDCl$_3$, 400 MHz) spectrum of **(*Z*)-3o**

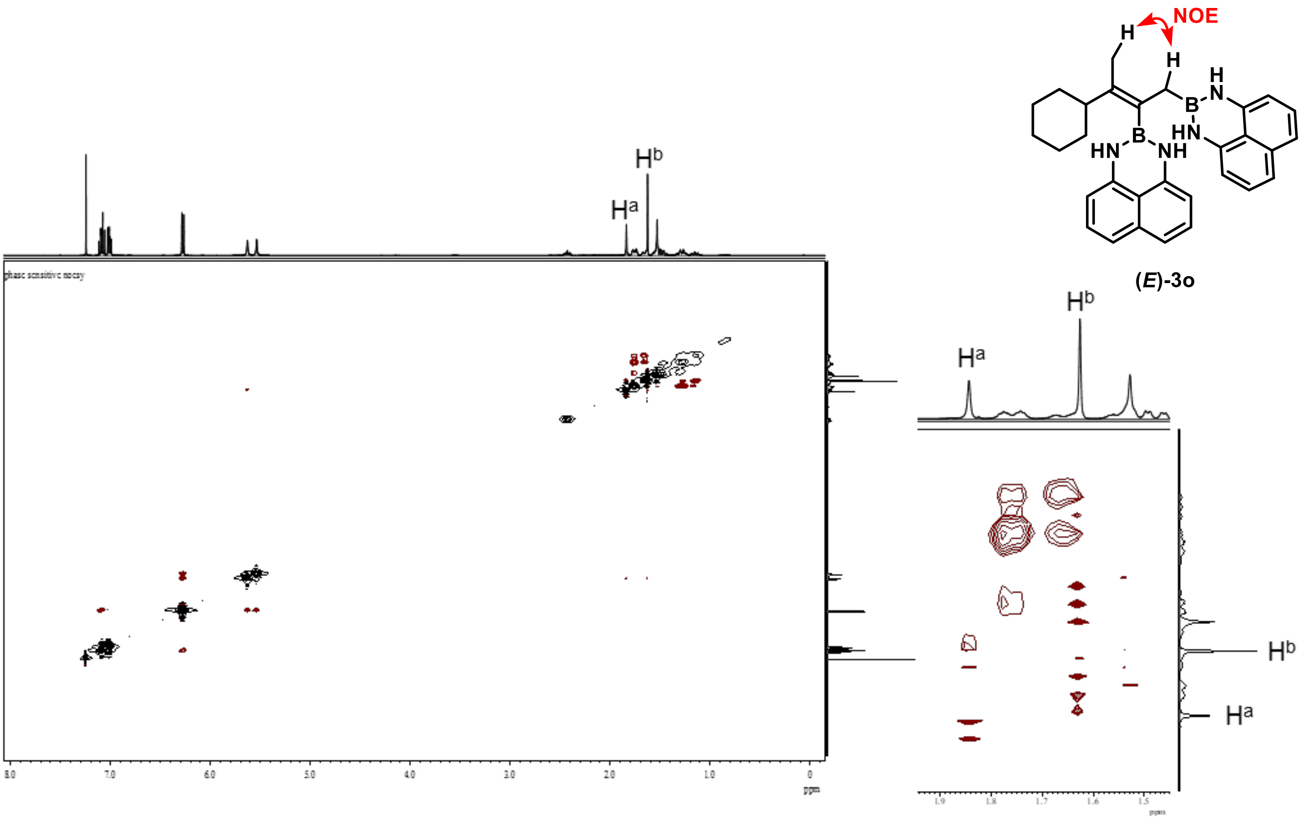

Figure S4. ^{1}H-^{1}H NOESY (CDCl$_3$, 400 MHz) spectrum of **(*E*)-3o**

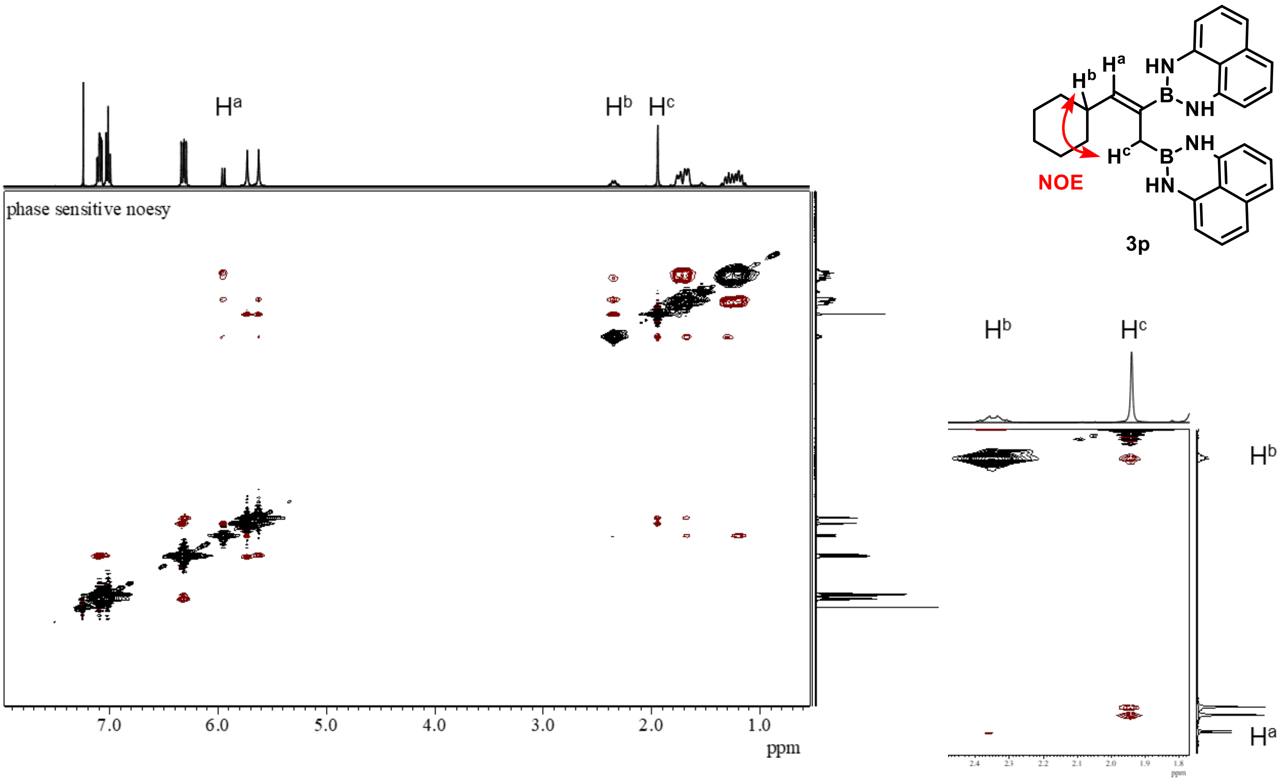

Figure S5. ^{1}H-^{1}H NOESY (CDCl$_3$, 400 MHz) spectrum of **3p**

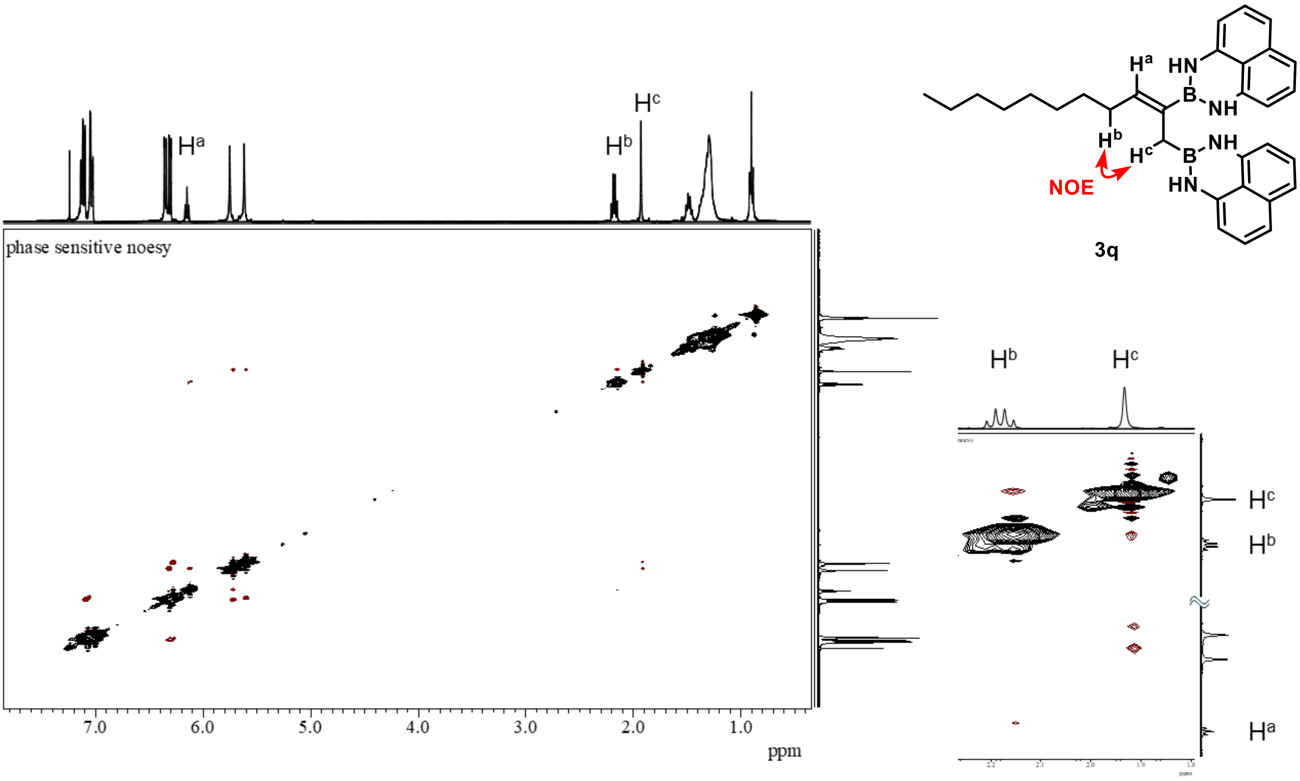

Figure S6. ^{1}H-^{1}H NOESY (CDCl$_3$, 400 MHz) spectrum of **3q**

VII. Derivatization of 3a

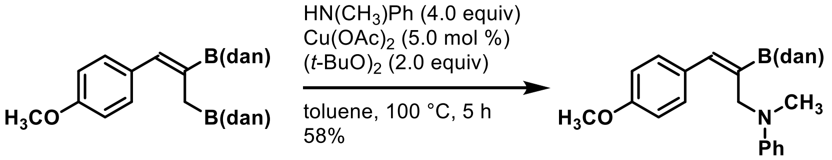

3a **4a**

According to the literature,[21,22] a pressure tube was charged with **3a** (48 mg, 0.10 mmol, 1.0 equiv), $Cu(OAc)_2$ (0.91 mg, 0.0050 mmol, 5.0 mol %), toluene (0.10 mL), (*t*-BuO)$_2$ (0.037 mL, 0.20 mmol, 2.0 equiv) and $HN(CH_3)Ph$ (0.043 mL, 0.40 mmol, 4.0 equiv). After stirring for 5 h at 100 ºC, the resulting mixture was filtered through a pad of silica gel (AcOEt). Solvents were removed under reduced pressure and the residue was purified by silica gel column chromatography (hexane/AcOEt = 20/1) to afford **4a** (24 mg, 0.058 mmol, 58%).

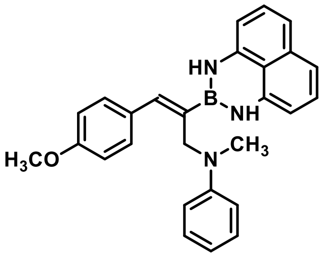

4a

(*Z*)-*N*-(3-(4-Methoxyphenyl)-2-(1H-naphtho[1,8-de][1,3,2]diazaborinin-2(3H)-yl)allyl)-*N*-methylaniline (**4a**): White amorphous solid. ^{1}H NMR ($CDCl_3$, 400 MHz): δ 7.34 (d, *J* = 8.4 Hz, 2H), 7.26-7.22 (m, 2H), 7.04 (t, *J* = 7.6 Hz, 2H), 6.98 (dd, *J* = 8.4, 1.1 Hz, 2H), 6.88-6.74 (m, 6H), 6.10 (dd, *J* = 7.2, 1.1 Hz, 2H), 5.70 (s, 2H), 4.11 (d, *J* = 1.5 Hz, 2H), 3.76 (s, 3H), 2.95 (s, 3H); ^{13}C{^{1}H} NMR ($CDCl_3$, 100 MHz): δ 159.3, 150.4, 141.1, 139.5, 136.5, 130.6, 129.9, 129.4, 127.8, 120.0, 117.8, 117.6, 114.04, 113.96, 106.1, 61.7, 55.5, 38.5, the signal of the boron-bound carbon was obscure due to the quadrupolar boron nucleus; ^{11}B{^{1}H} NMR ($CDCl_3$, 128 MHz): δ 31.3; IR (ATR): 3393, 3051, 2931, 2834, 1602, 1507 cm^{-1}; HRMS (ESI/Q-TOF) *m/z*: $[M + H]^+$ calcd for $C_{27}H_{27}{}^{10}BN_3O$ 419.2278. Found 419.2278.

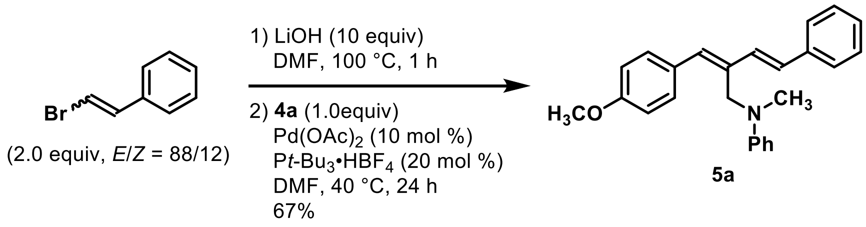

According to the literature,[23] a Schlenk tube was charged with *β*-bromostyrene (0.077 mL, 0.60 mmol, 2.0 equiv), LiOH (72 mg, 3.0 mmol, 10 equiv) and DMF (1.2 mL). The mixture was stirred for 1 h at 100 ºC. Then **4a** (130 mg, 0.30 mmol, 1.0 equiv), $Pd(OAc)_2$ (6.7 mg, 0.030 mmol, 10 mol %), P*t*-Bu_3·HBF_4 (17 mg, 0.060 mmol, 20 mol %) was added. After stirring for 24 h at 40 ºC, water was added. The aqueous phase was extracted with AcOEt (× 3). The combined organic phase was washed with brine and dried over Na_2SO_4. Solvents were removed under reduced pressure and the residue was purified by silica gel column chromatography (hexane/AcOEt = 20/1) to afford **5a** (71 mg, 0.20 mmol, 67%).

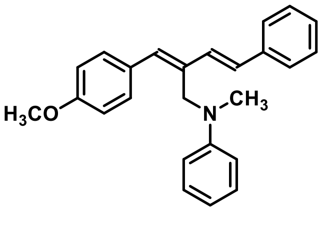

5a

***N*-((*E*)-2-((*Z*)-4-Methoxybenzylidene)-4-phenylbut-3-en-1-yl)-*N*-methylaniline** (**5a**): Yellow solid. Mp: 103.2-103.9 ºC; ^{1}H NMR ($CDCl_3$, 400 MHz): δ 7.37-7.27 (m, 5H), 7.25-7.18 (m, 5H), 6.87 (d, *J* = 7.6 Hz, 2H), 6.75-6.65 (m, 4H), 6.48 (s, 1H), 4.28 (d, *J* = 0.9 Hz, 2H), 3.79 (s, 3H), 3.03 (s, 3H); $^{13}C\{^1H\}$ NMR ($CDCl_3$, 100 MHz): δ 158.8, 150.0, 137.8, 131.5, 131.1, 130.1, 129.3, 129.1, 128.9, 128.7, 127.7, 126.6, 126.0, 116.5, 113.9, 112.3, 56.3, 55.5, 38.5; IR (ATR): 3035, 2903, 1603, 1507, 1252 cm^{-1}; HRMS (ESI/Q-TOF) *m/z*: $[M + H]^+$ calcd for $C_{25}H_{26}NO$ 356.2009. Found 356.2011.

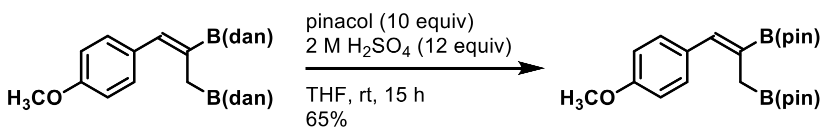

3a **3r**

According to the procedure by Hall,[24] to a mixture of **3a** (48 mg, 0.10 mmol, 1.0 equiv), pinacol (120 mg, 1.0 mmol, 10 equiv) in THF (1 mL) was added 2 M aq. H_2SO_4 (0.60 mL, 1.2 mmol, 12 equiv). After stirring for 15 h at room temperature, water was added. The aqueous phase was extracted with Et_2O (× 3). The combined organic phase was washed with brine and dried over Na_2SO_4. Solvents were removed under reduced pressure and the residue was purified by silica gel column chromatography (hexane/AcOEt = 10/1) to afford **3r** (26 mg, 0.065 mmol, 65%).

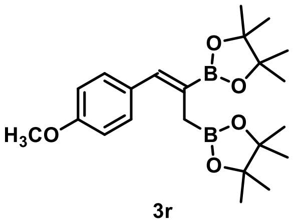

3r

(*Z*)-2,2'-(3-(4-Methoxyphenyl)prop-2-ene-1,2-diyl)bis(4,4,5,5-tetramethyl-1,3,2-dioxaborolane) (**3r**): White solid. ^{1}H NMR ($CDCl_3$, 400 MHz): δ 7.30 (d, J = 8.0 Hz, 2H), 7.13 (s, 1H), 6.83 (d, J = 8.4 Hz, 2H), 3.78 (s, 3H), 2.03 (s, 2H), 1.26 (s, 12H), 1.21 (s, 12H). The NMR data matched those reported previously.[18]

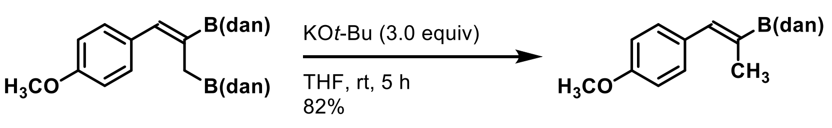

3a **6a**

To a solution of **3a** (48 mg, 0.10 mmol, 1.0 equiv) in THF (1 mL) was added 1 M KO*t*-Bu/THF (0.90 mL, 0.30 mmol, 3.0 equiv). After stirring for 5 h at room temperature, the resulting mixture was quenched by brine. The aqueous phase was extracted with AcOEt (× 3). The combined organic phase was washed with brine and dried over Na_2SO_4. Solvents were removed under reduced pressure and the residue was purified by silica gel column chromatography (hexane/AcOEt = 10/1) to afford **6a** (26 mg, 0.082 mmol, 82%).

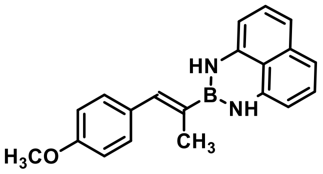

6a

(*Z*)-2-(1-(4-Methoxyphenyl)prop-1-en-2-yl)-2,3-dihydro-1H-naphtho[1,8-de][1,3,2]diazaborinine (**6a**): White solid. Mp: 122.2-123.2 °C; ^{1}H NMR ($CDCl_3$, 400 MHz): δ 7.32 (d, J = 8.8 Hz, 2H), 7.12 (t, J = 7.8 Hz, 2H), 7.02 (d, J = 8.0 Hz, 2H), 6.93-6.91 (m, 3H), 6.36 (dd, J = 7.3, 0.8 Hz, 2H), 5.83 (s, 2H), 3.83 (s, 3H), 2.03 (d, J = 1.6 Hz, 3H); ^{13}C{^{1}H} NMR ($CDCl_3$, 100 MHz): δ 158.8, 141.4, 136.8, 136.5, 130.8, 130.7, 127.8, 119.9, 117.7, 113.8, 106.0, 55.5, 16.0, the signal of the boron-bound carbon was obscure due to the quadrupolar boron nucleus; ^{11}B{^{1}H} NMR ($CDCl_3$, 128 MHz): δ 29.6; IR (ATR): 3444, 3424, 3406, 3045, 3007, 2971, 2838, 1629, 1604, 1517, 819, 779 cm^{-1}; HRMS (ESI/Q-TOF) *m/z*: $[M + H]^+$ calcd for $C_{20}H_{20}{}^{10}BN_2O$ 314.1700. Found 314.1700.

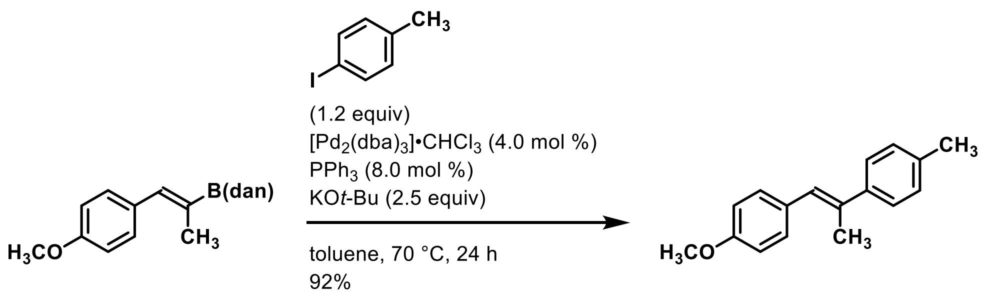

6a **7a**

According to the literature,[25] a Schlenk tube was charged with **6a** (16 mg, 0.050 mmol, 1.0 equiv), $[Pd_2(dba)_3]\cdot CHCl_3$ (2.1 mg, 0.0020 mmol, 4.0 mol %), PPh_3 (1.0 mg, 0.0040 mmol, 8.0 mol %), 4-iodotoluene (13 mg, 0.060 mmol, 1.2 equiv) and degassed toluene (1 mL). 1 M KO*t*-Bu/THF (0.12 mL, 0.12 mmol, 2.5 equiv) was added and the reaction mixture was stirred for 24 h at 70 ºC. To the resulting mixture was added brine and the aqueous phase was extracted with AcOEt (× 3). The combined organic phase was washed with brine and dried over Na_2SO_4. Solvents were removed under reduced pressure and the residue was purified by silica gel column chromatography (hexane/AcOEt = 20/1) to afford **7a** (11 mg, 0.046 mmol, 92%).

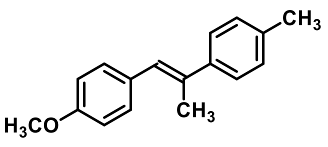

7a

(*E*)-1-Methoxy-4-(2-(p-tolyl)prop-1-en-1-yl)benzene (**7a**): White solid. ^1H NMR ($CDCl_3$, 400 MHz): δ 7.40 (d, *J* = 8.4 Hz, 2H), 7.29 (d, *J* = 8.4 Hz, 2H), 7.16 (d, *J* = 7.6 Hz, 2H), 6.90 (d, *J* = 8.8 Hz, 2H), 6.75 (s, 1H), 3.82 (s, 3H), 2.35 (s, 3H), 2.25 (d, *J* = 1.6 Hz, 3H). The NMR data matched those reported previously.[26]

References

(1) Kuang, J.; Ma, S. An Efficient Synthesis of Terminal Allenes from Terminal 1-Alkynes. *J. Org. Chem.* **2009**, *74* (4), 1763−1765. DOI: 10.1021/jo802391x.

(2) Bolte, B.; Odabachian, Y.; Gagosz, F. Gold(I)-Catalyzed Rearrangement of Propargyl Benzyl Ethers: A Practical Method for the Generation and in Situ Transformation of Substituted Allenes. *J. Am Chem. Soc.* **2010**, *132* (21), 7294−7296. DOI: 10.1021/ja1020469.

(3) Clavier, H.; Jeune, K. L.; Riggi, I. d.; Tenaglia, A.; Buono, G. Highly Selective Cobalt-Mediated [6 + 2] Cycloaddition of Cycloheptatriene and Allenes. *Org. Lett.* **2011**, *13* (2), 308−311. DOI: 10.1021/ol102783x.

(4) Li, Q.-H.; Liao, J.-W.; Huang, Y.-L.; Chiang, R.-T.; Gau, H.-M. Nickel-catalyzed substitution reactions of propargyl halides with organotitanium reagents. *Org. Biomol. Chem.* **2014**, *12* (38), 7634−7642. DOI: 10.1039/C4OB00677A.

(5) Nakamura, H.; Kamakura, T.; Ishikura, M.; Biellmann, J.-F. Synthesis of Allenes via Palladium-Catalyzed Hydrogen-Transfer Reactions: Propargylic Amines as an Allenyl Anion Equivalent. *J. Am. Chem. Soc.* **2004**, *126* (19), 5958−5959. DOI: 10.1021/ja039175+.

(6) Furuyama, T.; Yonehara, M.; Arimoto, S.; Kobayashi, M.; Matsumoto, Y.; Uchiyama, M. Development of Highly Chemoselective Bulky Zincate Complex, $^{t}Bu_4ZnLi_2$: Design, Structure, and Practical Applications in Small-/Macromolecular Synthesis. *Chem. Eur. J.* **2008**, *14* (33), 10348−10356. DOI: 10.1002/chem.200800536.

(7) Rae, J.; Hu, Y. C.; Procter, D. J. Cu(I)–NHC-Catalyzed Silylation of Allenes: Diastereoselective Three-Component Coupling with Aldehydes. *Chem. Eur. J.* **2014**, *20* (41), 13143−13145. DOI: 10.1002/chem.201404330.

(8) Buechi, G.; Wuest, H. Transformation of nitrimines to acetylenes and allenes. 1,3 Rearrangement of N-nitroenamines to C-nitro compounds. *J. Org. Chem.* **1979**, *44* (23), 4116−4120. DOI: 10.1021/jo01337a021.

(9) Hossain, M. L.; Ye, F.; Zhang, Y.; Wang, J. CuI-Catalyzed Cross-Coupling of N-Tosylhydrazones with Terminal Alkynes: Synthesis of 1,3-Disubstituted Allenes. *J. Org. Chem.* **2013**, *78* (3), 1236−1241. DOI: 10.1021/jo3024686.

(10) Elsevier, C. J.; Vermeer, P. Stereochemistry of the palladium(0)-catalyzed phenylation of 1-halooallenes. *J. Org. Chem.* **1985**, *50* (17), 3042−3045. DOI: 10.1021/jo00217a004.

(11) Oda, S.; Sam, B.; Krische, M. J. Hydroaminomethylation Beyond Carbonylation: Allene–Imine Reductive Coupling by Ruthenium-Catalyzed Transfer Hydrogenation. *Angew. Chem., Int. Ed.* **2015**, *54* (29), 8525−8528. DOI: 10.1002/anie.201503250.

(12) Kim, Y.; Lee, H.; Park, S.; Lee, Y. Copper-Catalyzed Propargylic Reduction with Diisobutylaluminum Hydride. *Org. Lett.* **2018**, *20* (17), 5478−5481. DOI: 10.1021/acs.orglett.8b02413.

(13) Kippo, T.; Fukuyama, T.; Ryu, I. Regioselective Radical Bromoallylation of Allenes Leading to 2-Bromo-Substituted 1,5-Dienes. *Org. Lett.* **2011**, *13* (15), 3864−3867. DOI: 10.1021/ol201395p.

(14) Crandall, J. K.; Batal, D. J.; Sebesta, D. P.; Lin, F. 1,4-Dioxaspiro[2.2]pentanes. Synthesis, spectroscopic properties, and reactions with nucleophiles. *J. Org. Chem.* **1991**, *56* (3), 1153−1166. DOI: 10.1021/jo00003a044.

(15) Tani, Y.; Fujihara, T.; Terao, J.; Tsuji, Y. Copper-Catalyzed Regiodivergent Silacarboxylation of Allenes with Carbon Dioxide and a Silylborane. *J. Am. Chem. Soc.* **2014**, *136* (51), 17706−17709. DOI: 10.1021/ja512040c.

(16) Danheiser, R. L.; Choi, Y. M.; Menichincheri, M.; Stoner, E. J. Synthesis of allenes via thermal cycloreversion

of .alpha.-alkylidene-.beta.-lactones. *J. Org. Chem.* **1993**, *58* (2), 322–327. DOI: 10.1021/jo00054a011.

(17) Yasuda, T.; Yoshigoe, Y.; Saito, S. Copper-Catalyzed Borylation of Styrenes by 1,8-Diaminonaphthalene-Protected Diboronic Acid. *Org. Lett.* **2023**, *25* (12), 2093–2097. DOI: 10.1021/acs.orglett.3c00451.

(18) Kidonakis, M.; Stratakis, M. Regioselective Diboration and Silaboration of Allenes Catalyzed by Au Nanoparticles. *ACS Catal.* **2018**, *8* (2), 1227–1230. DOI: 10.1021/acscatal.7b04084.

(19) Burks, H. E.; Liu, S.; Morken, J. P. Development, Mechanism, and Scope of the Palladium-Catalyzed Enantioselective Allene Diboration. *J. Am. Chem. Soc.* **2007**, *129* (28), 8766–8773. DOI: 10.1021/ja070572k.

(20) Guo, X.; Nelson, A. K.; Slebodnick, C.; Santos, W. L. Regio- and Chemoselective Diboration of Allenes with Unsymmetrical Diboron: Formation of Vinyl and Allyl Boronic Acid Derivatives. *ACS Catal.* **2015**, *5* (4), 2172–2176. DOI: 10.1021/acscatal.5b00387.

(21) Sueki, S.; Kuninobu, Y. Copper-Catalyzed *N*- and *O*-Alkylation of Amines and Phenols using Alkylborane Reagents. *Org. Lett.* **2013**, *15* (7), 1544-1547, DOI: 10.1021/ol400323z.

(22) Yoshida, H.; Murashige, Y.; Osaka, I. Copper-Catalyzed B(dan)-Installing Allylic Borylation of Allylic Phosphates. *Adv. Synth. Catal.* **2019***, 361,* 2286-2290, DOI: 10.1002/adsc.201900342.

(23) Andoh, H.; Nakagawa, R.; Akutagawa, T.; Katata, E.; Tsuchimoto, T. Direct Suzuki–Miyaura cross-coupling of C(sp^2)–B(dan) bonds: designed in pursuit of usability. *Org. Chem. Front.* **2025**, *12*, 3759-3774, DOI: 10.1039/D5QO00230C.

(24) Lee, J. C. H.; McDonald, R.; Hall, D. G. Enantioselective preparation and chemoselective cross-coupling of 1,1-diboron compounds. *Nat. Chem.* **2011**, *3* (11), 894–899. DOI: 10.1038/nchem.1150.

(25) Mutoh, Y.; Yamamoto, K.; Saito, S. Suzuki–Miyaura Cross-Coupling of 1,8-Diaminonaphthalene (dan)-Protected Arylboronic Acids. *ACS Catal.* **2020**, *10* (1), 352-357, DOI: 10.1021/acscatal.9b03667.

(26) Yang, F.-L.; Ma, X.-T.; Tian, S.-K. Oxidative Mizoroki–Heck-Type Reaction of Arylsulfonyl Hydrazides for a Highly Regio- and Stereoselective Synthesis of Polysubstituted Alkenes. *Chem. Eur. J.* **2012**, *18* (6), 1582–1585. DOI: 10.1002/chem.201103671.

Copy of NMR Spectra

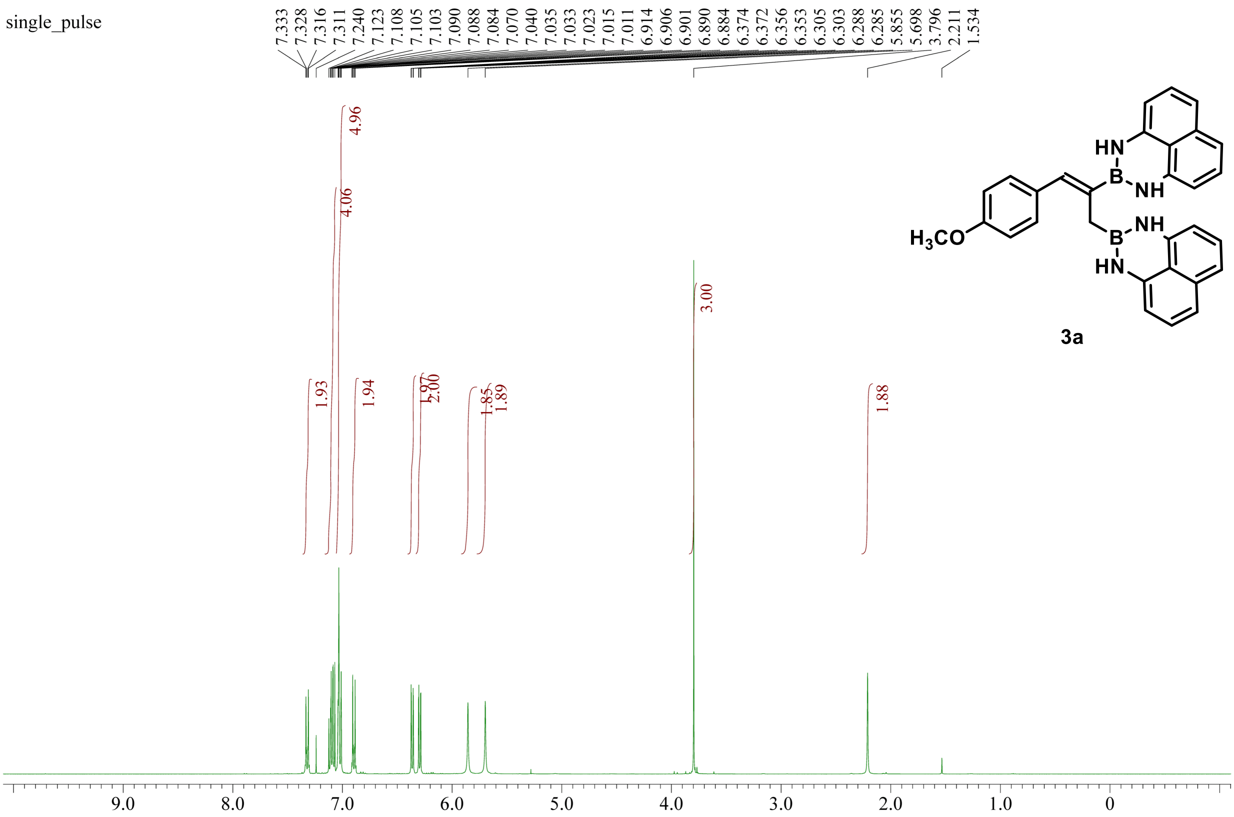

Figure S7. ^{1}H NMR ($CDCl_3$, 400 MHz) spectrum of **3a**

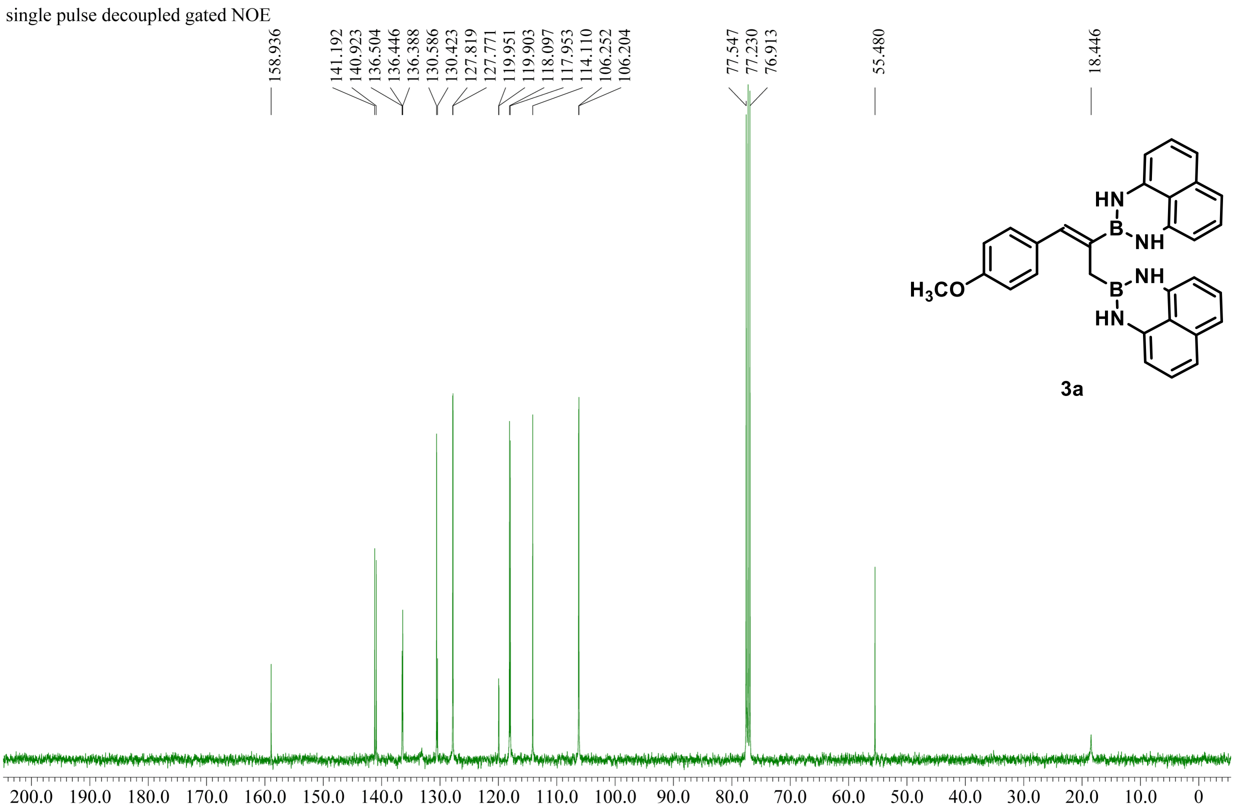

Figure S8. $^{13}C\{^{1}H\}$ NMR ($CDCl_3$, 100 MHz) spectrum of **3a**

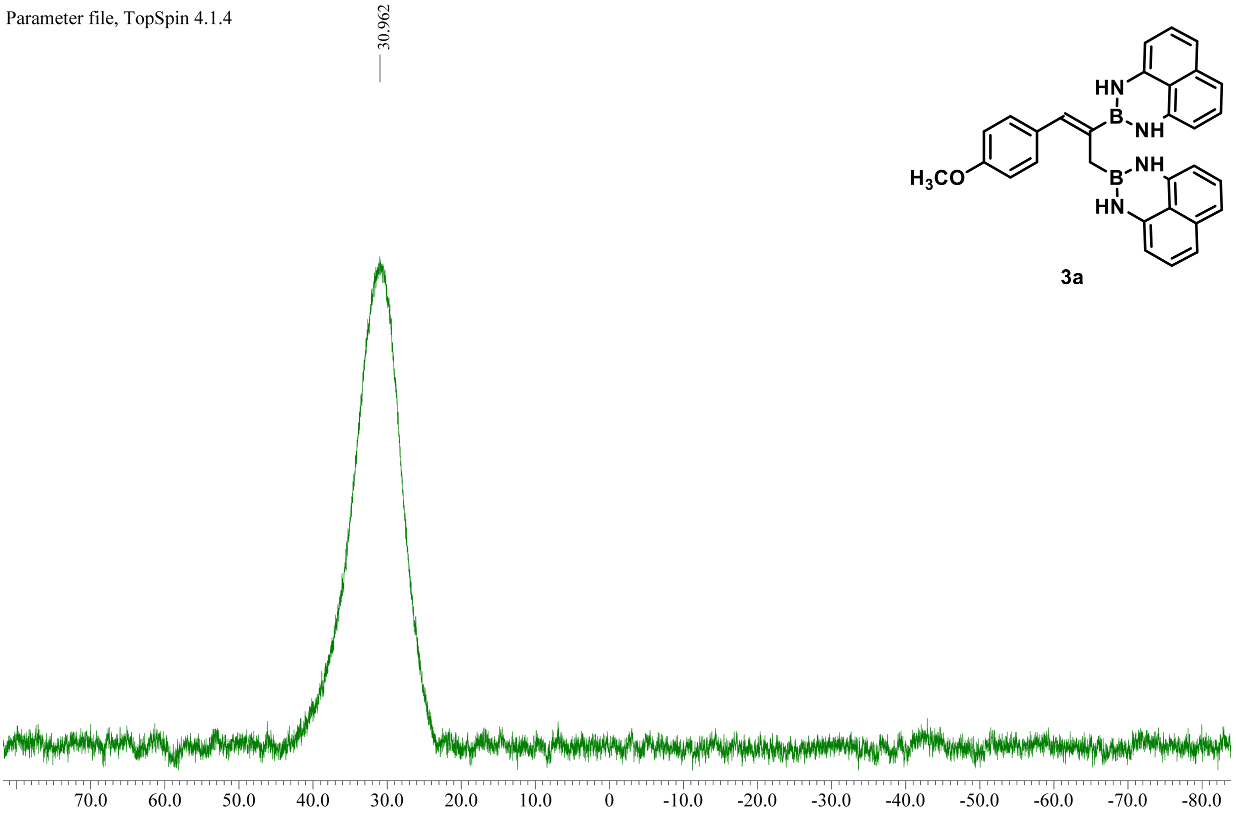

Figure S9. $^{11}B\{^{1}H\}$ NMR ($CDCl_3$, 128 MHz) spectrum of **3a**

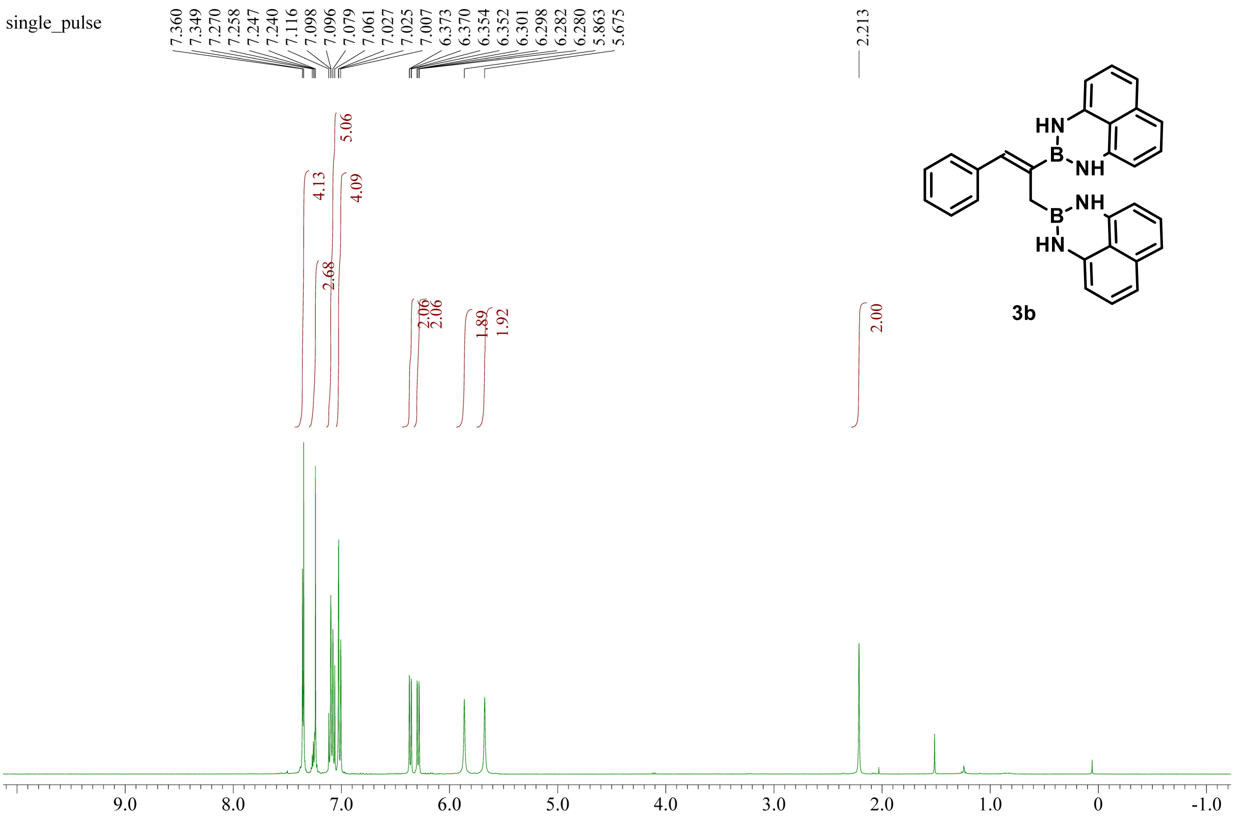

Figure S10. ^{1}H NMR ($CDCl_3$, 400 MHz) spectrum of **3b**

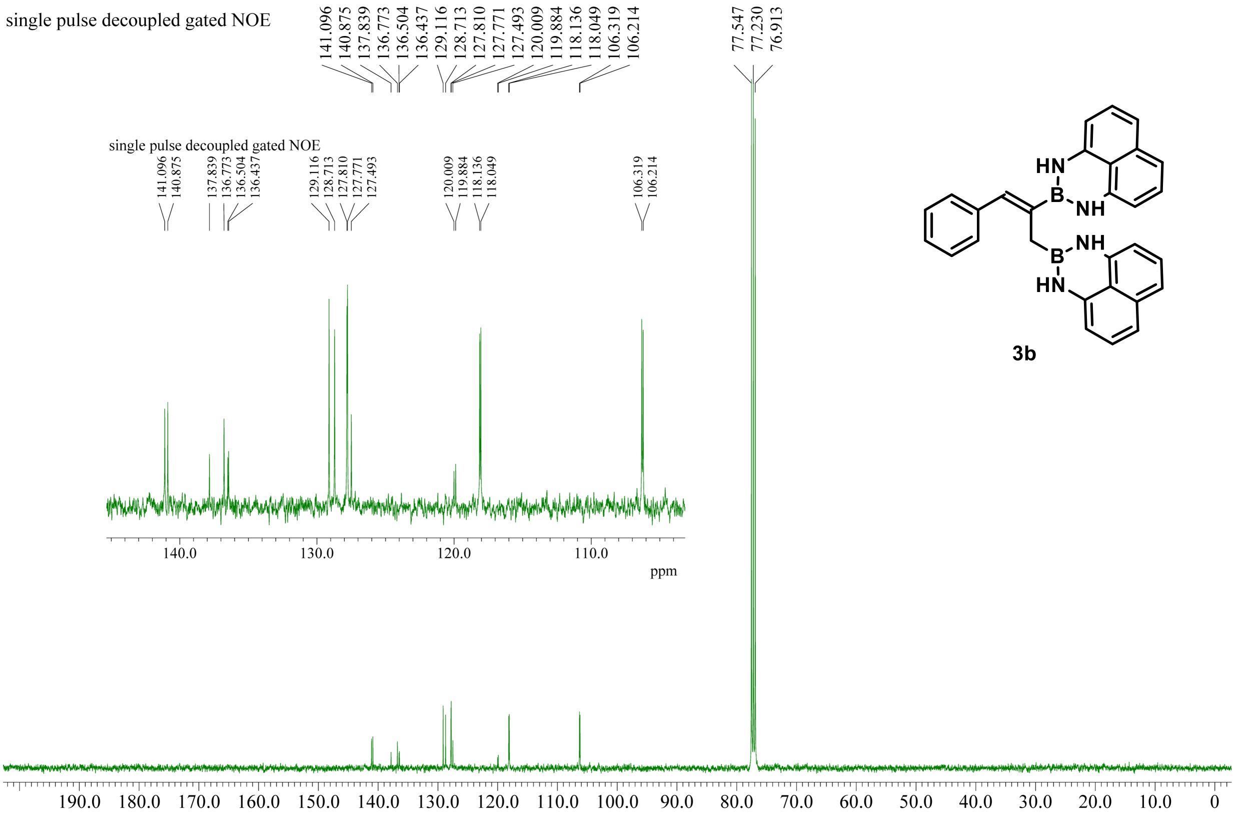

Figure S11. ^{13}C{^{1}H} NMR ($CDCl_3$, 100 MHz) spectrum of **3b**

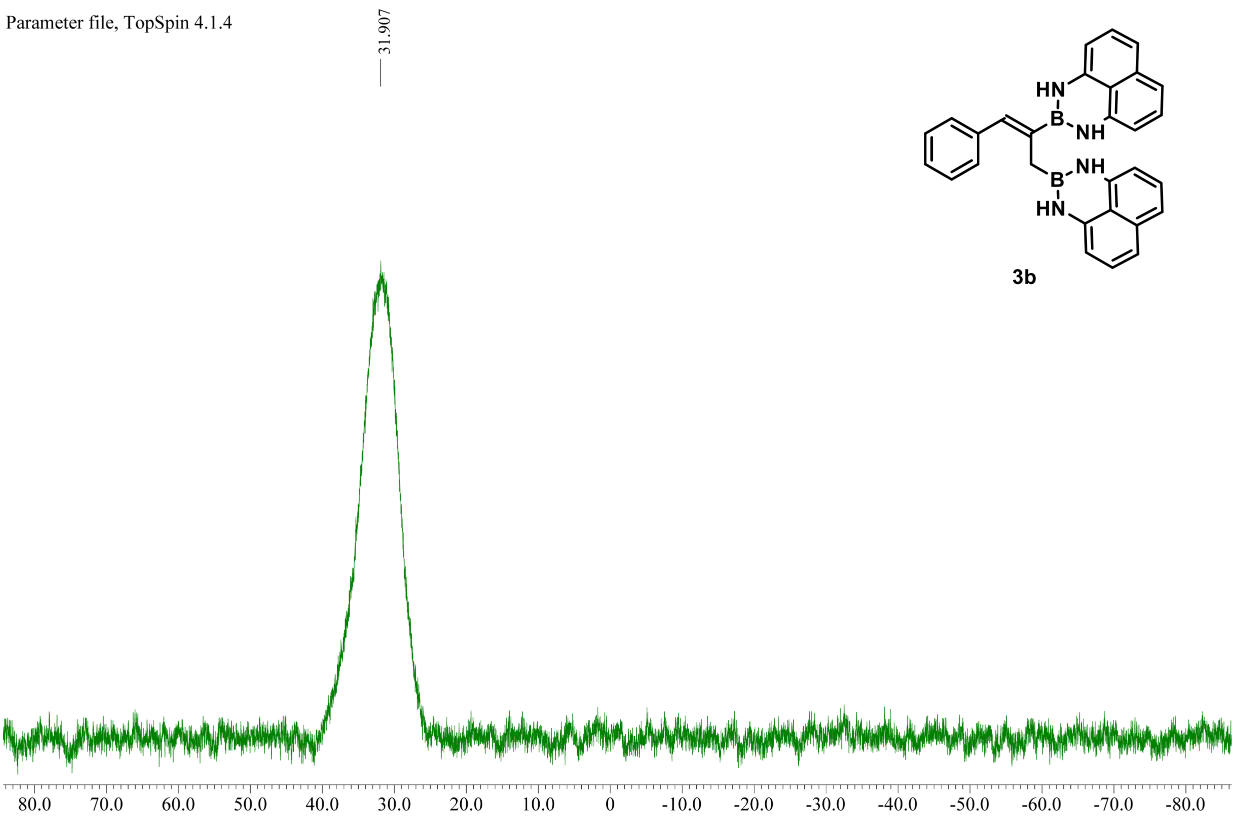

Figure S12. $^{11}B\{^{1}H\}$ NMR ($CDCl_3$, 128 MHz) spectrum of **3b**

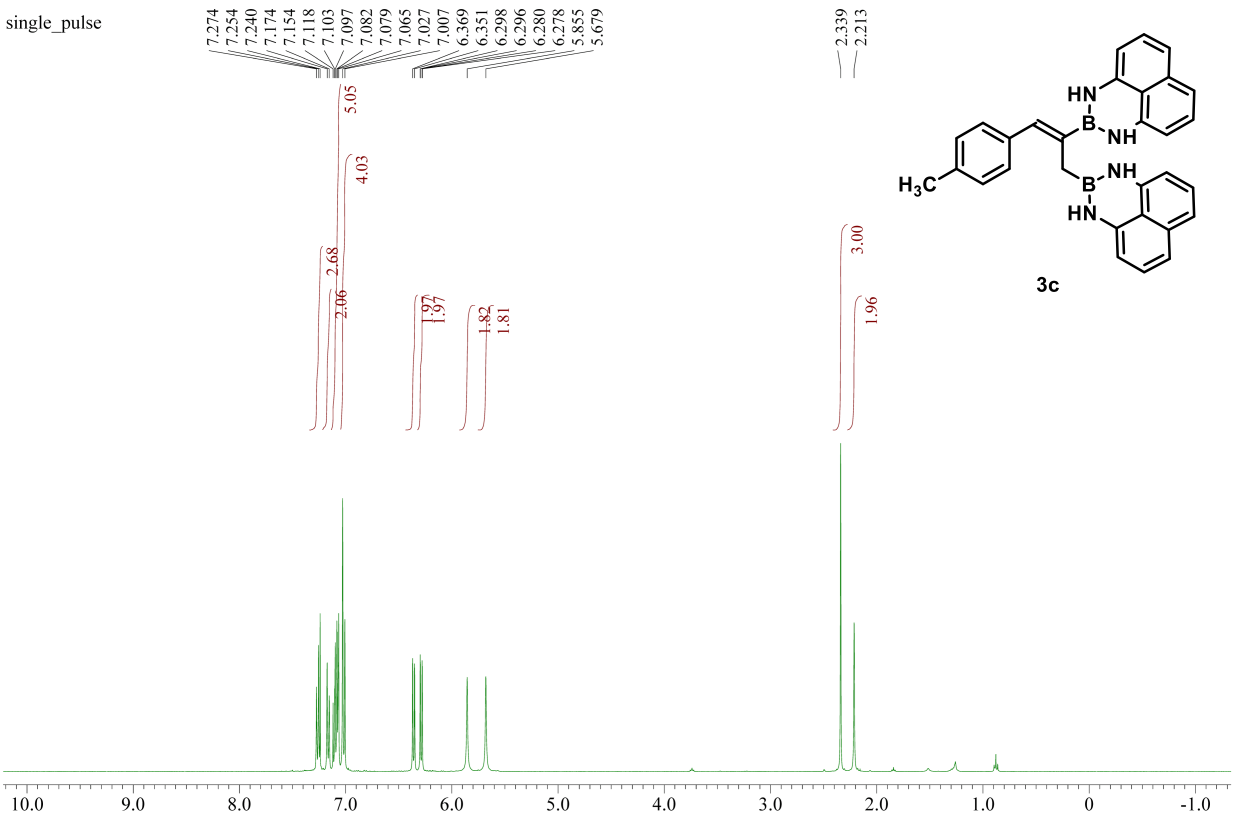

Figure S13. ^{1}H NMR ($CDCl_3$, 400 MHz) spectrum of **3c**

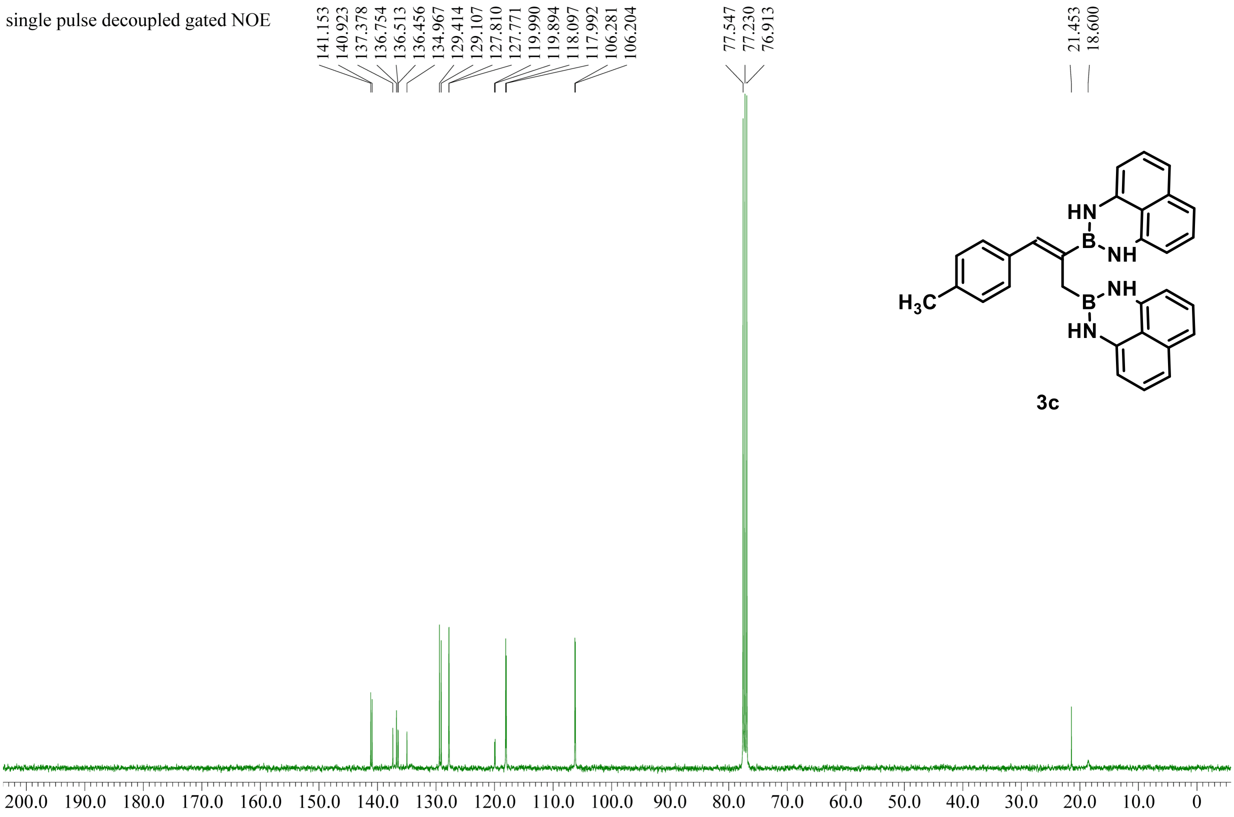

Figure S14. $^{13}C\{^{1}H\}$ NMR ($CDCl_3$, 100 MHz) spectrum of **3c**

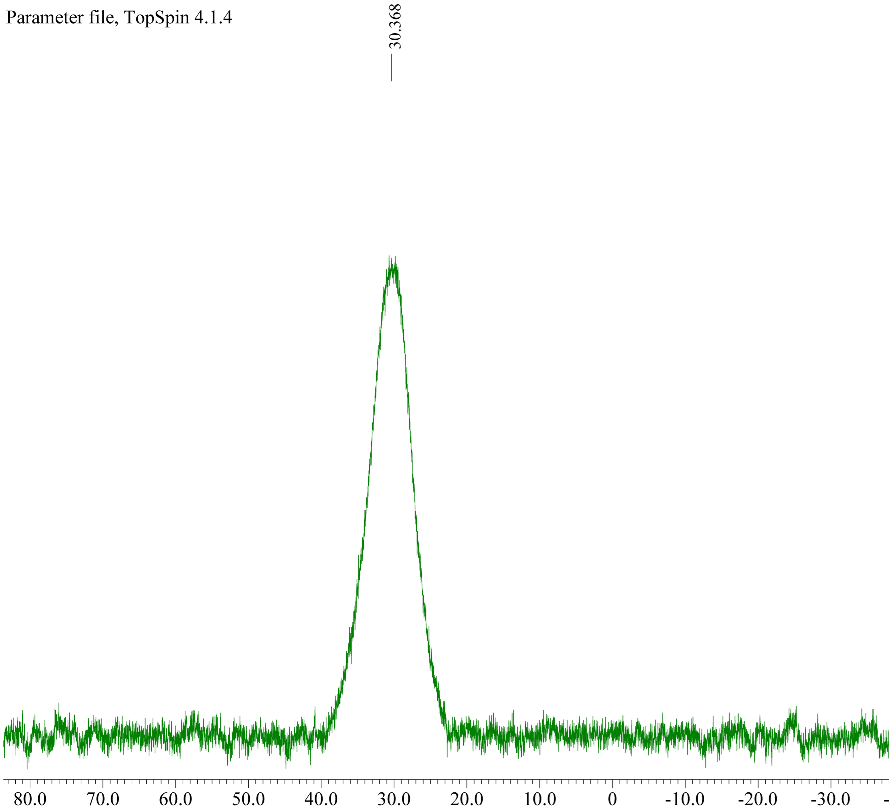

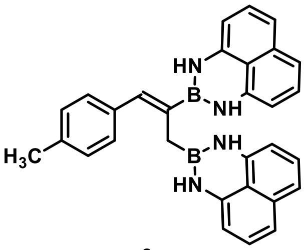

3c

Figure S15. $^{11}B\{^{1}H\}$ NMR ($CDCl_3$, 128 MHz) spectrum of **3c**

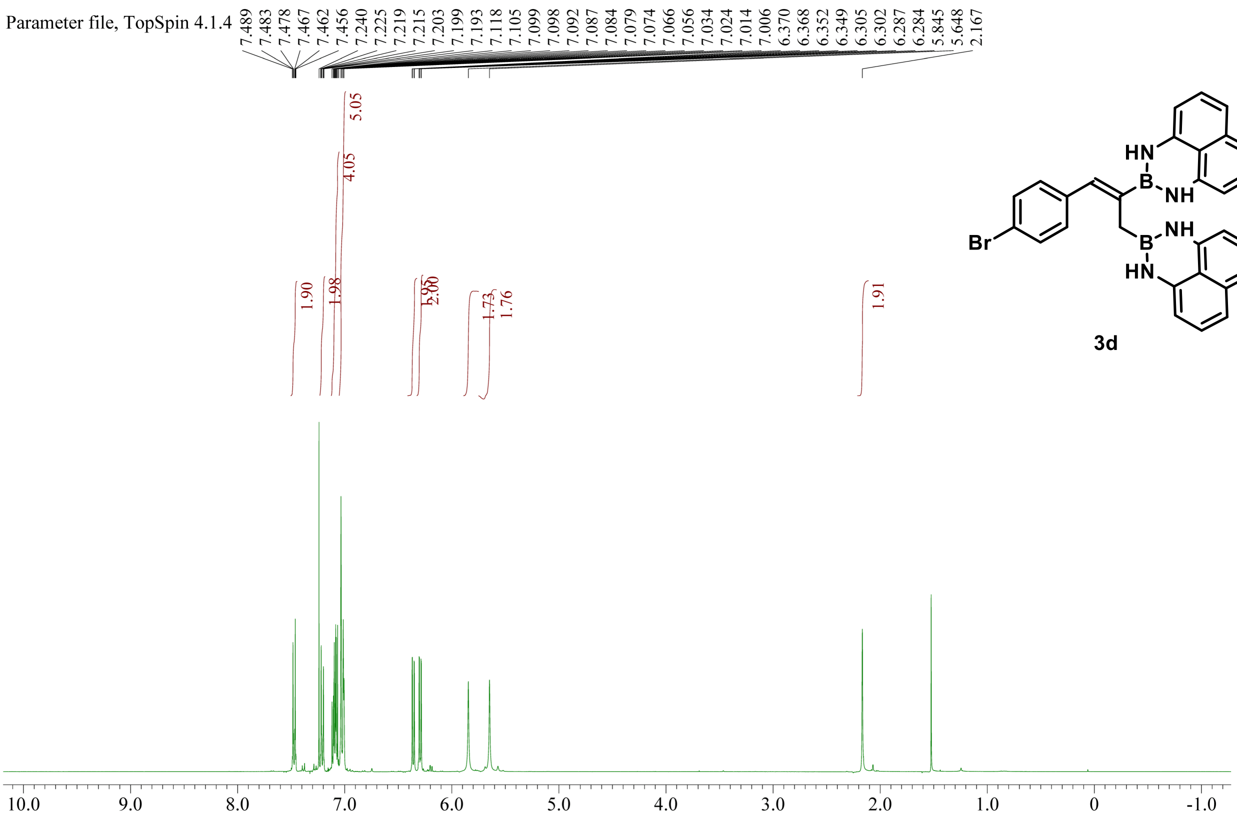

Figure S16. ^{1}H NMR (CDCl$_3$, 400 MHz) spectrum of **3d**

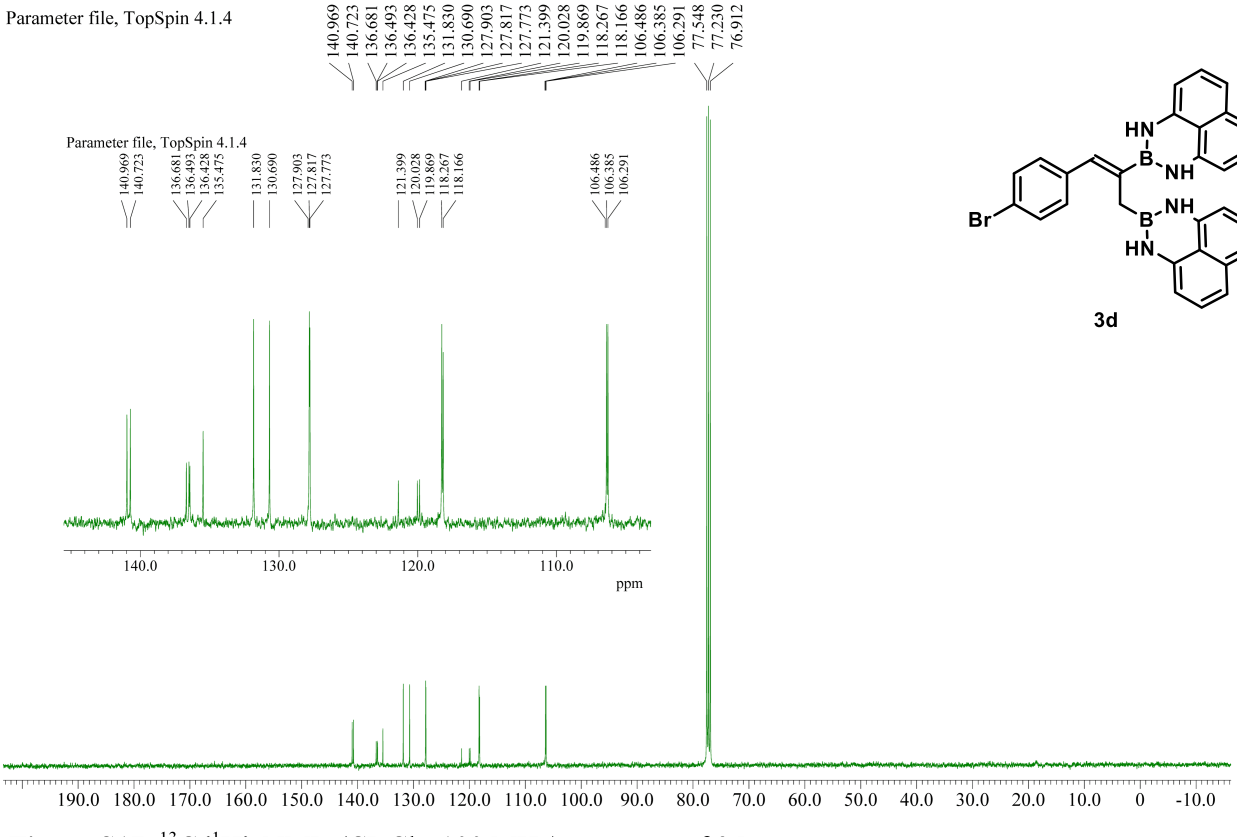

Figure S17. ^{13}C{^{1}H} NMR (CDCl$_3$, 100 MHz) spectrum of **3d**

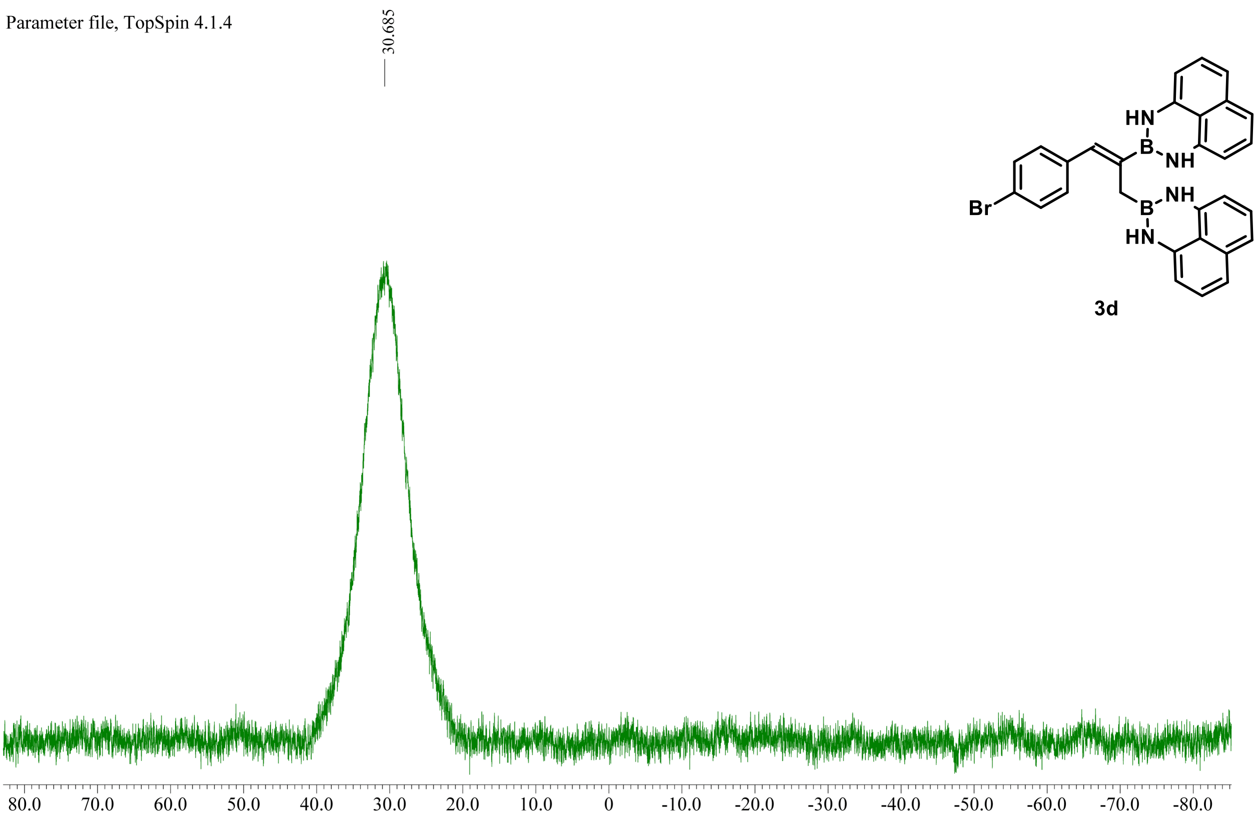

Figure S18. $^{11}B\{^{1}H\}$ NMR ($CDCl_3$, 128 MHz) spectrum of **3d**

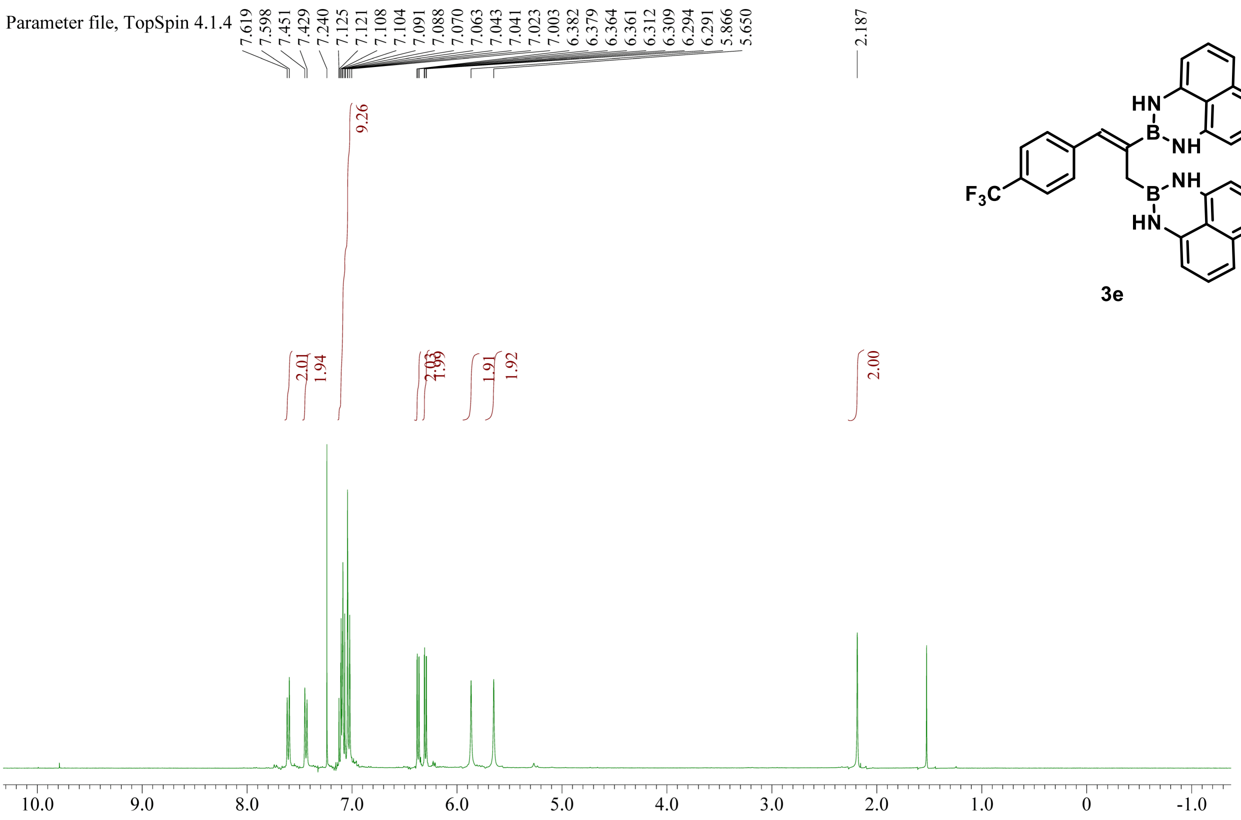

Figure S19. ^{1}H NMR ($CDCl_3$, 400 MHz) spectrum of **3e**

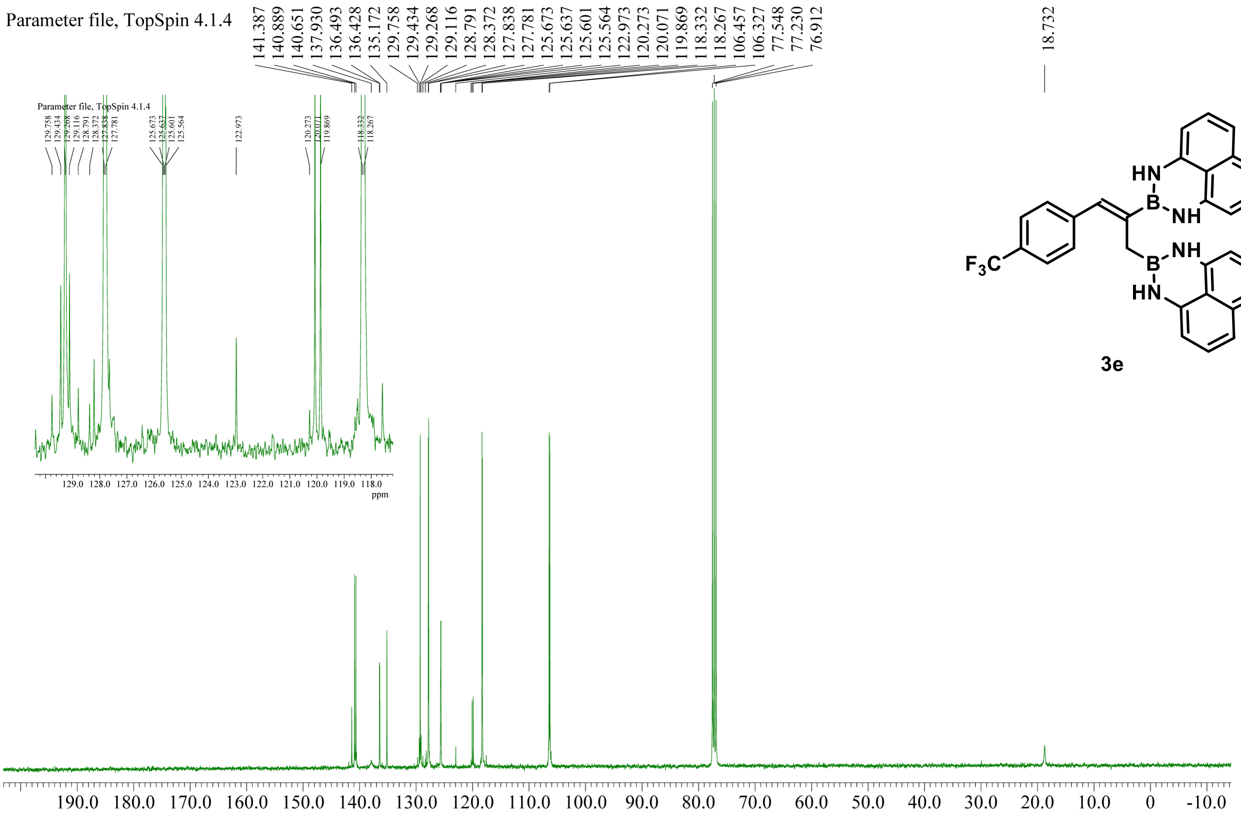

Figure S20. $^{13}C\{^{1}H\}$ NMR ($CDCl_3$, 100 MHz) spectrum of **3e**

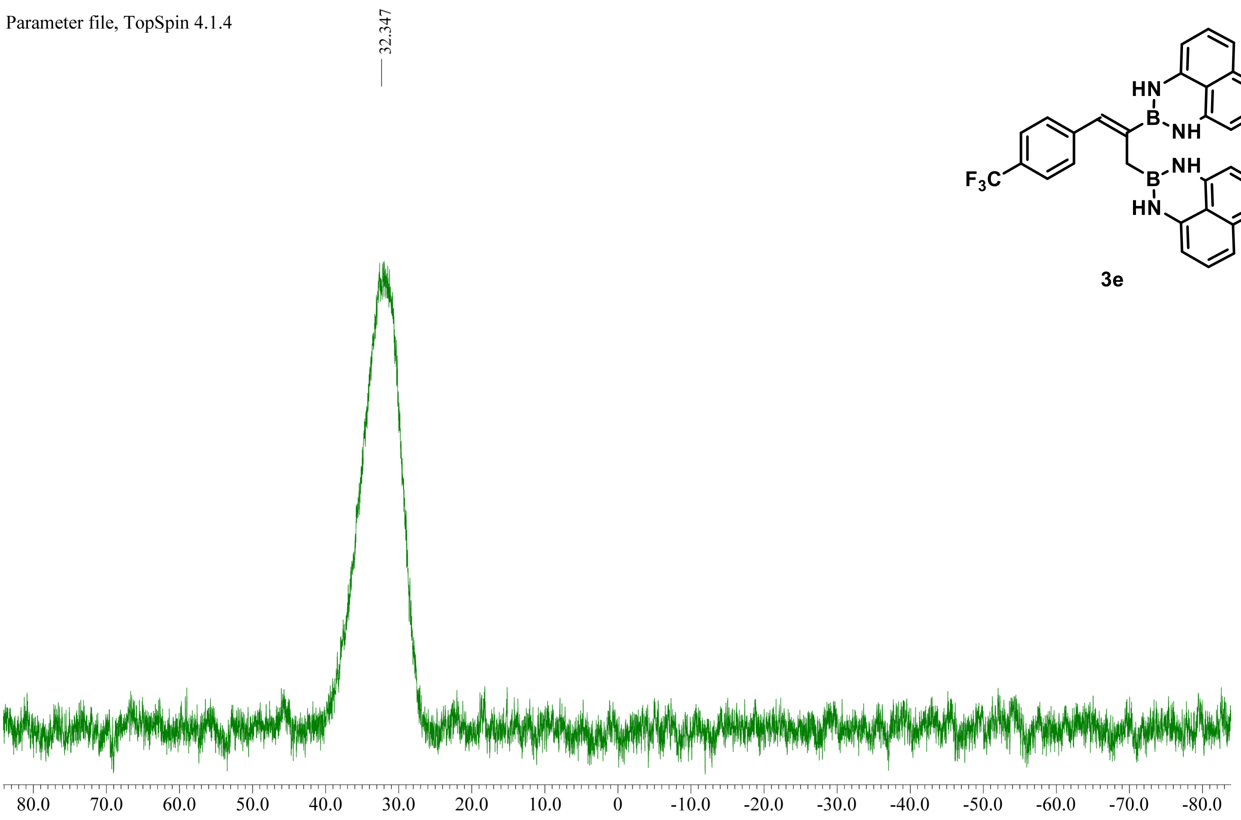

Figure S21. $^{11}B\{^{1}H\}$ NMR ($CDCl_3$, 128 MHz) spectrum of **3e**

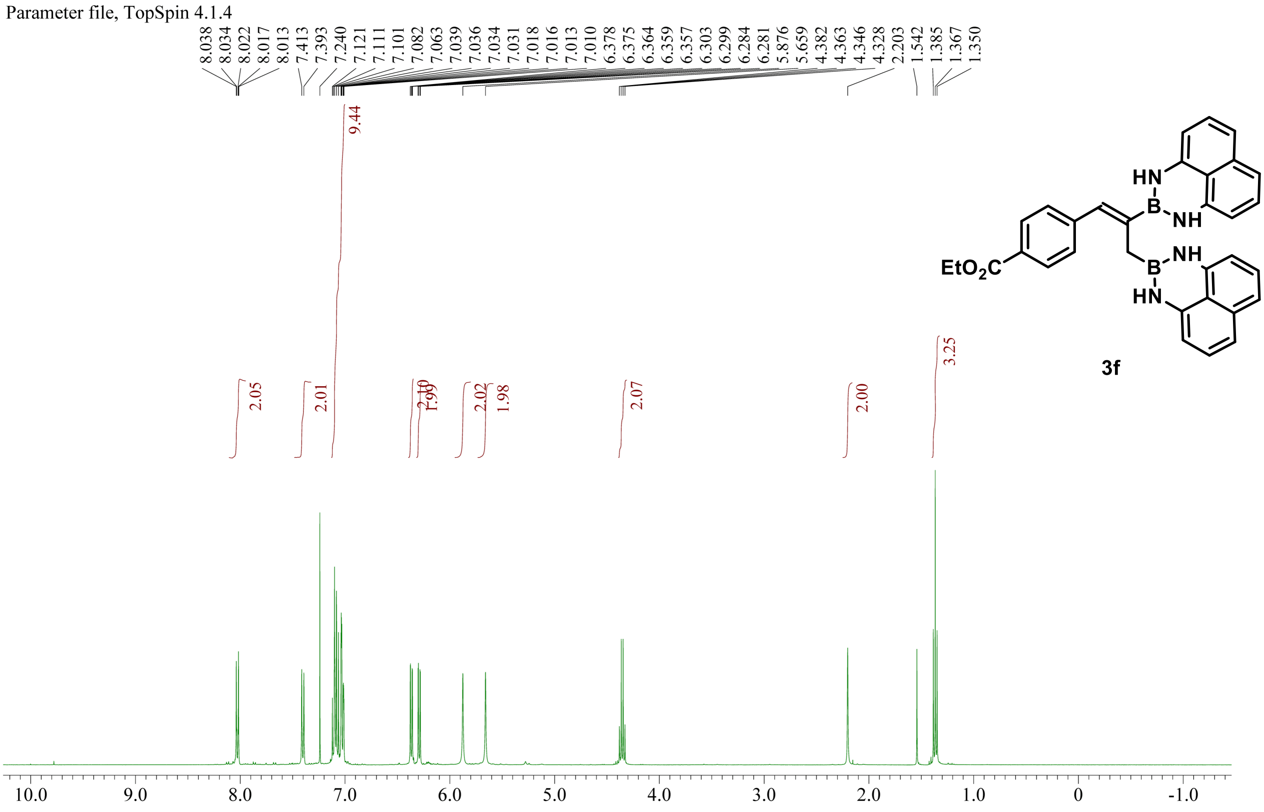

Figure S22. ^{1}H NMR ($CDCl_3$, 400 MHz) spectrum of **3f**

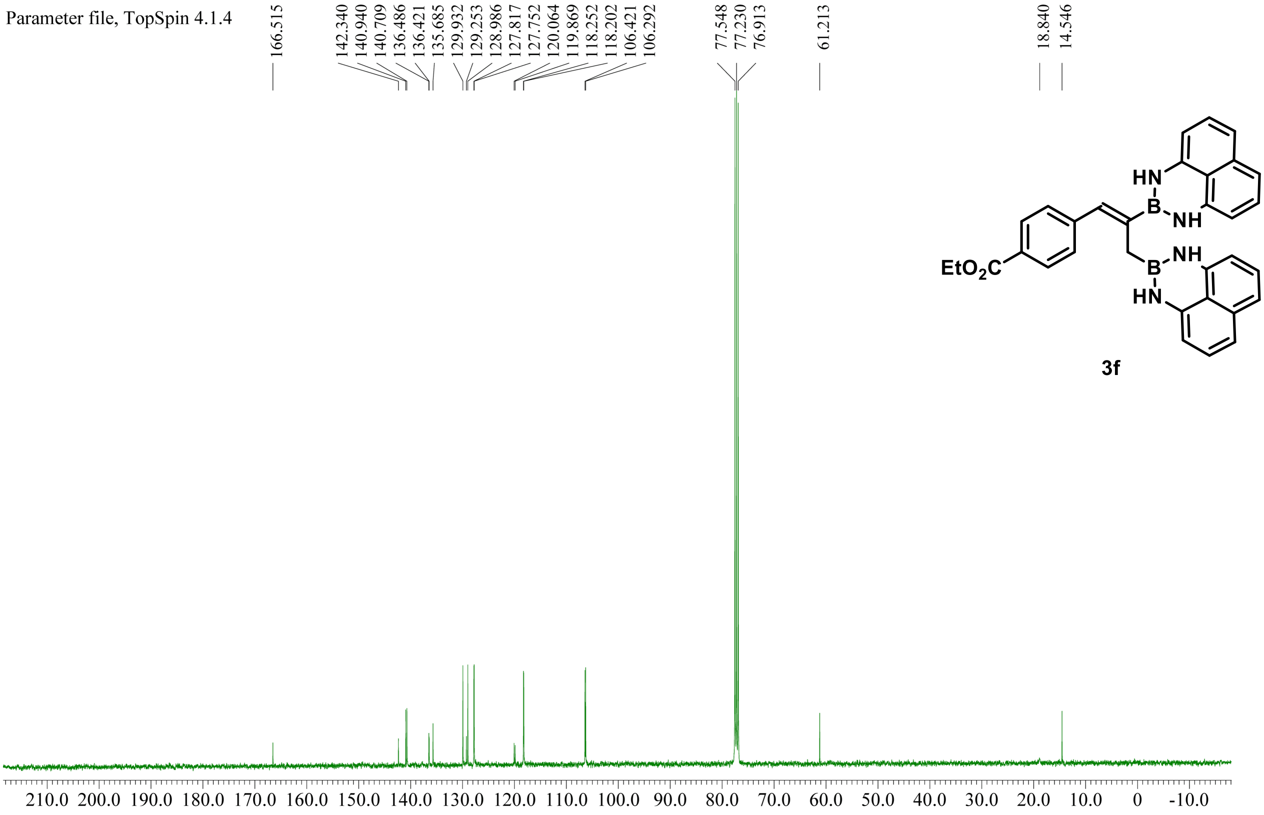

Figure S23. ^{13}C{^{1}H} NMR ($CDCl_3$, 100 MHz) spectrum of **3f**

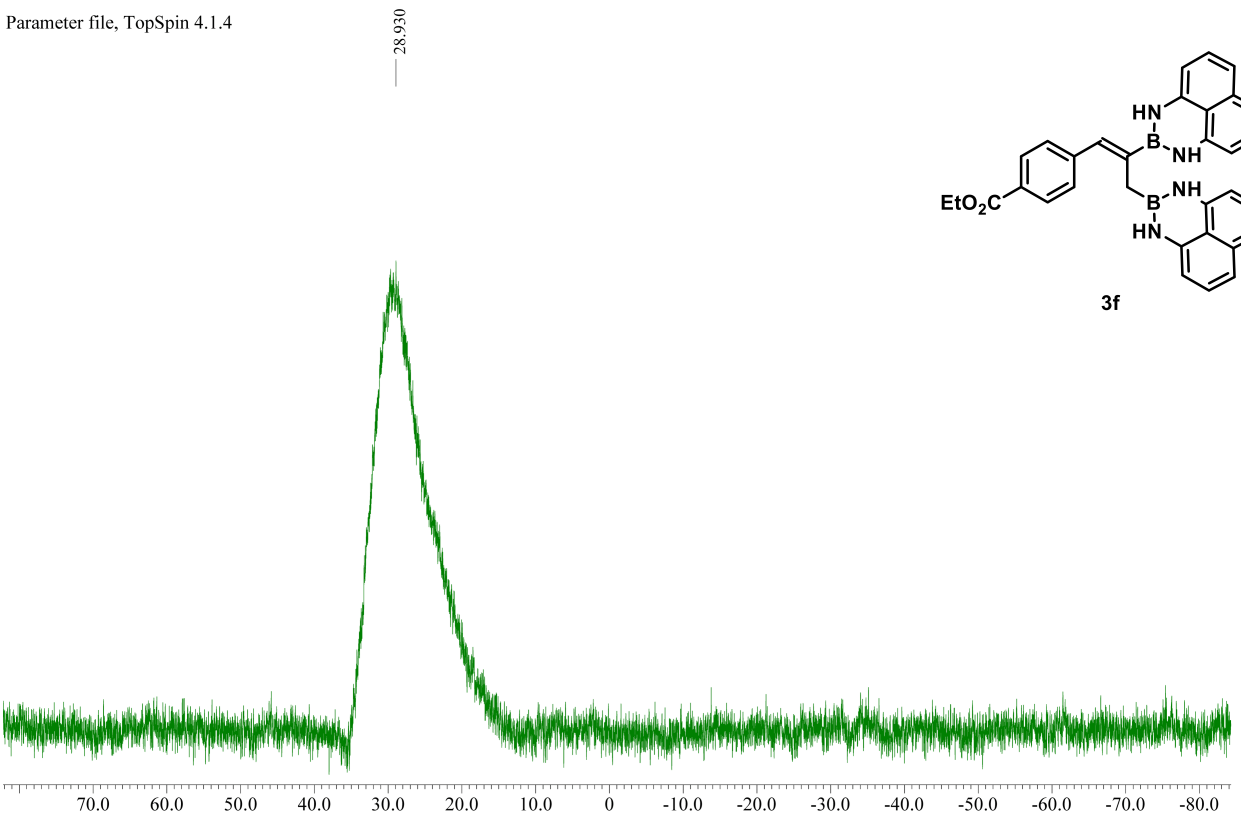

Figure S24. $^{11}B\{^{1}H\}$ NMR ($CDCl_3$, 128 MHz) spectrum of **3f**

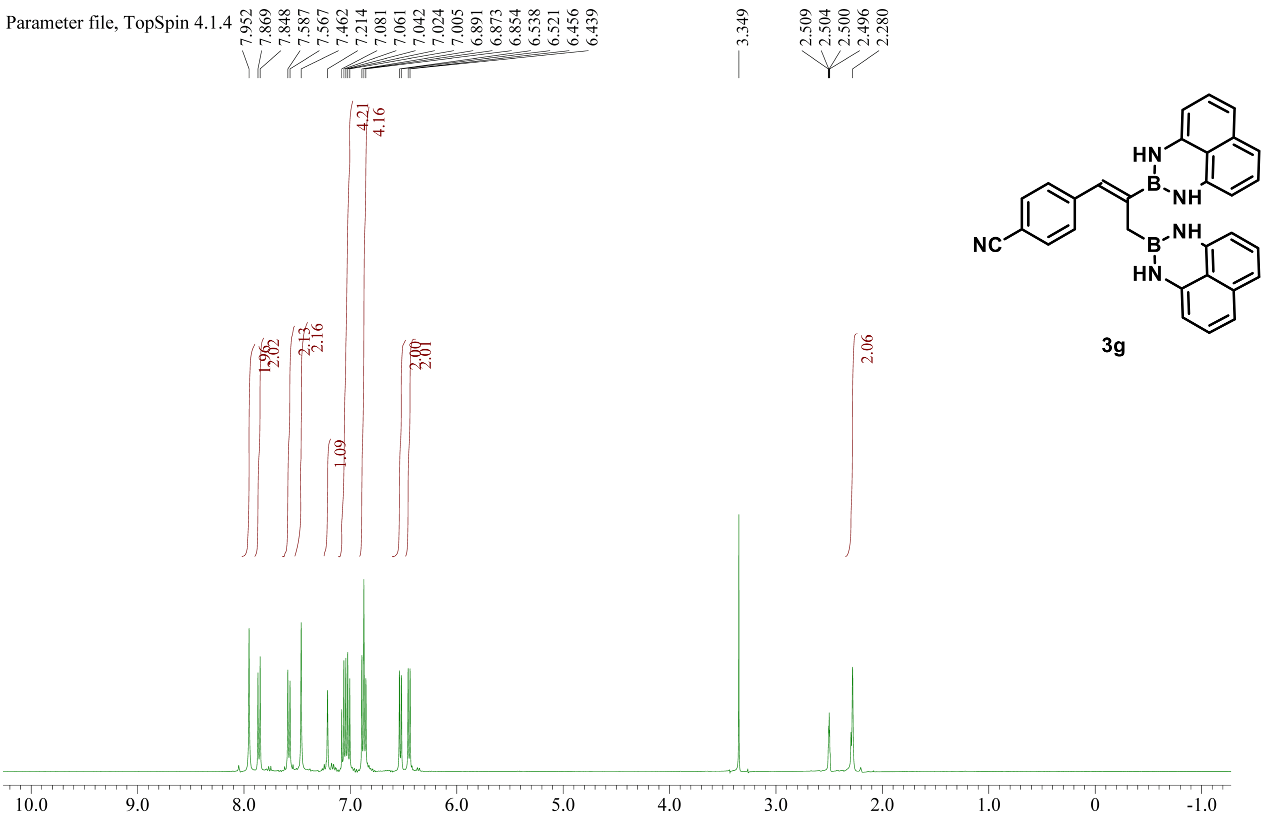

Figure S25. ^{1}H NMR (DMSO-d_6, 400 MHz) spectrum of **3g**

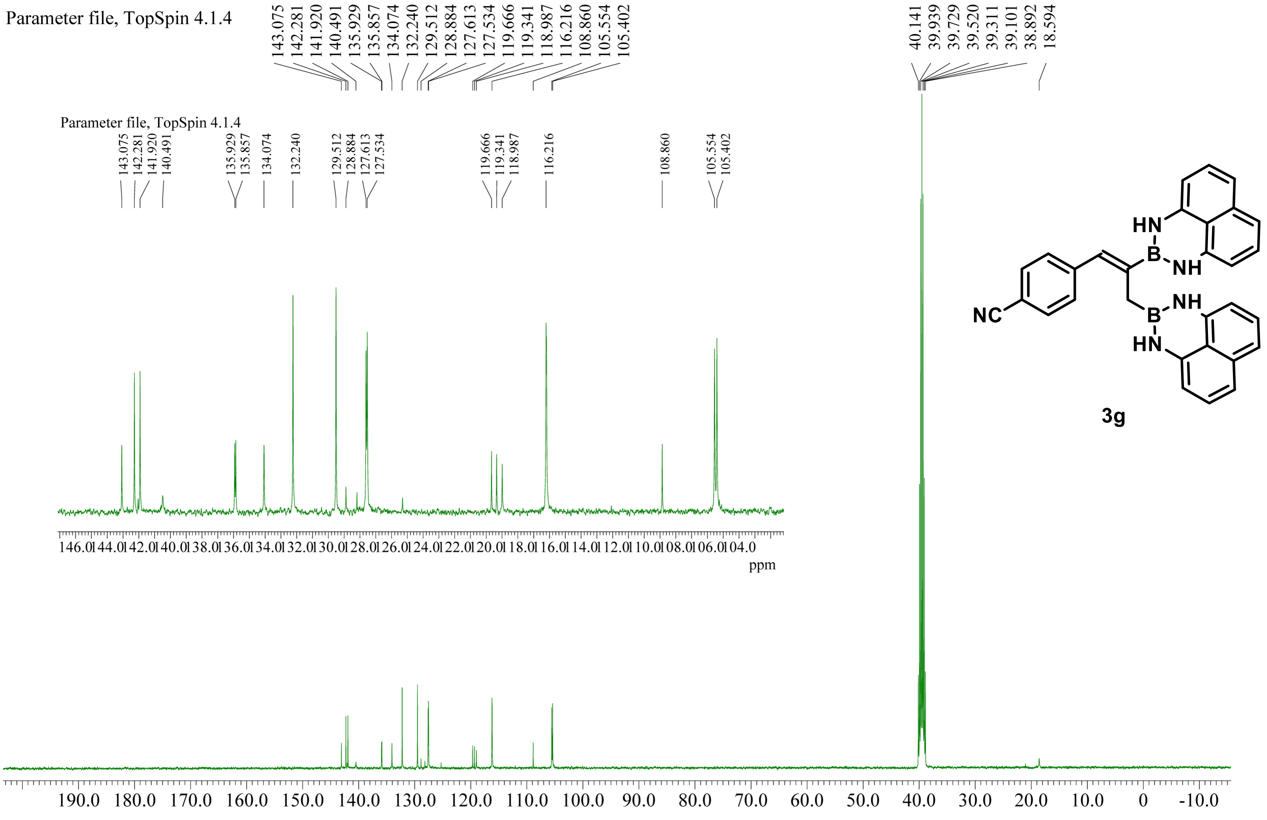

Figure S26. ^{13}C{^{1}H} NMR (DMSO-d_6, 100 MHz) spectrum of **3g**

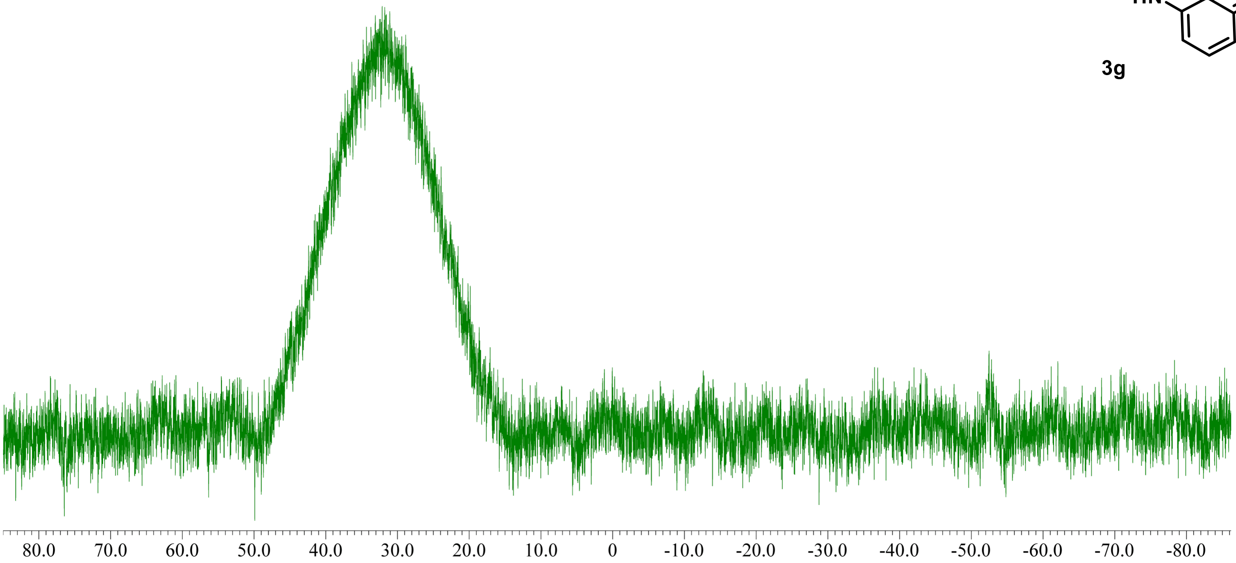

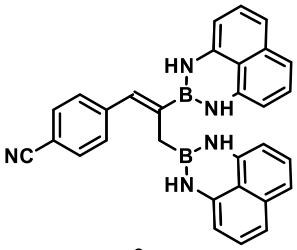

3g

Figure S27. $^{11}B\{^{1}H\}$ NMR (DMSO-d_6, 128 MHz) spectrum of **3g**

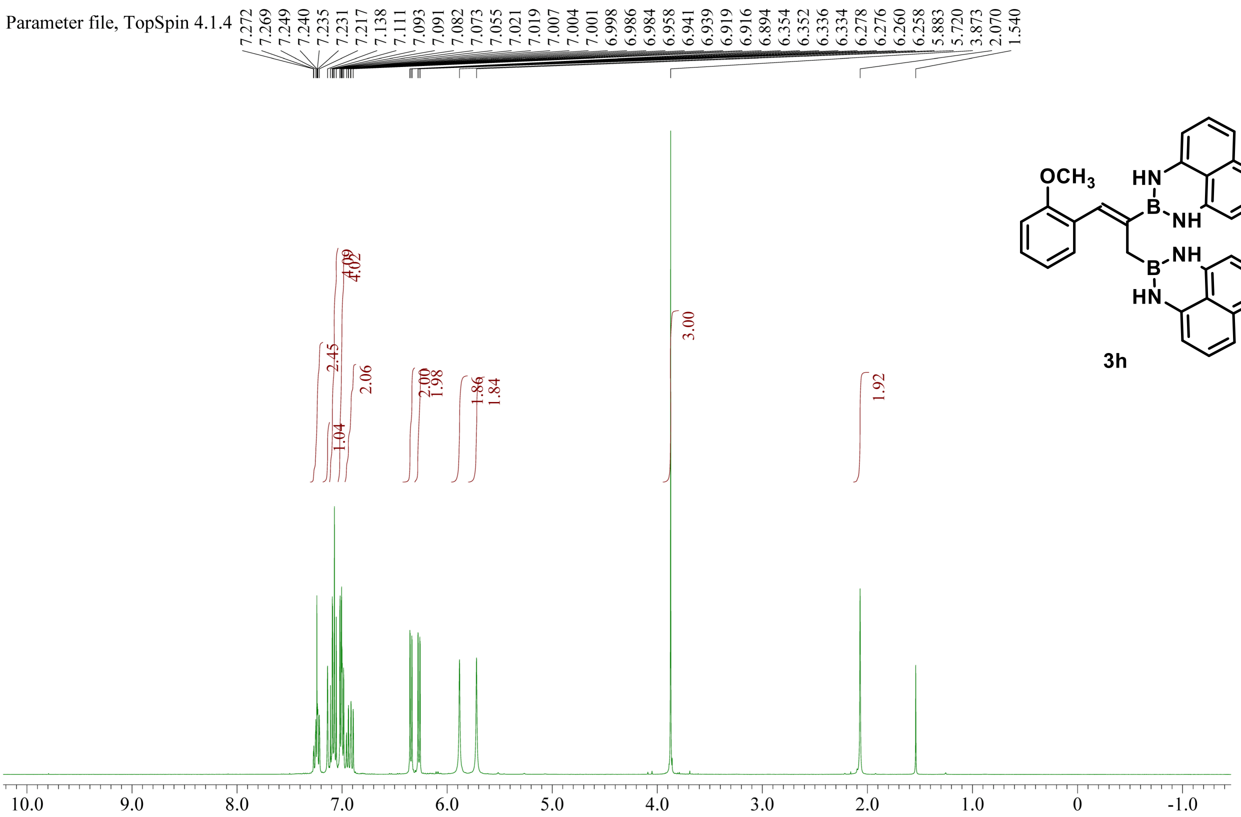

Figure S28. ^{1}H NMR ($CDCl_3$, 400 MHz) spectrum of **3h**

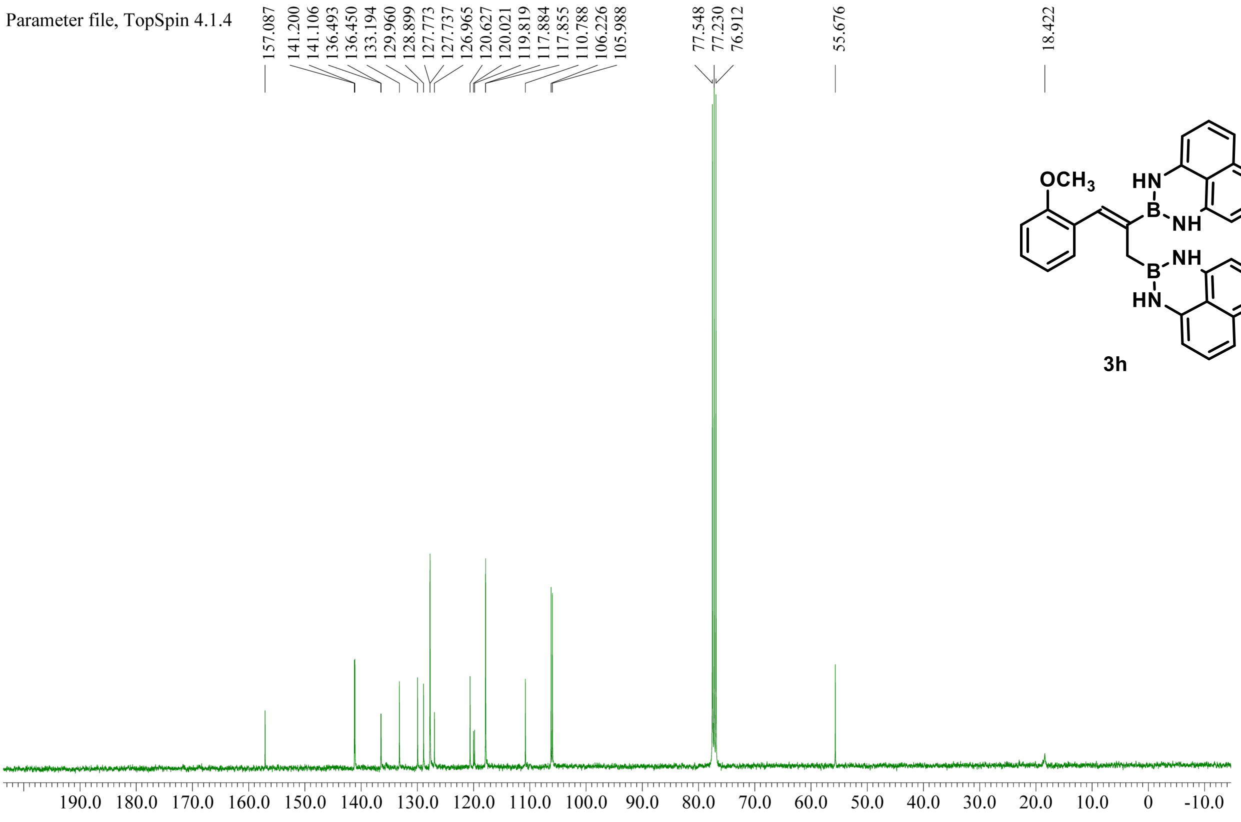

Figure S29. $^{13}C\{^{1}H\}$ NMR ($CDCl_3$, 100 MHz) spectrum of **3h**

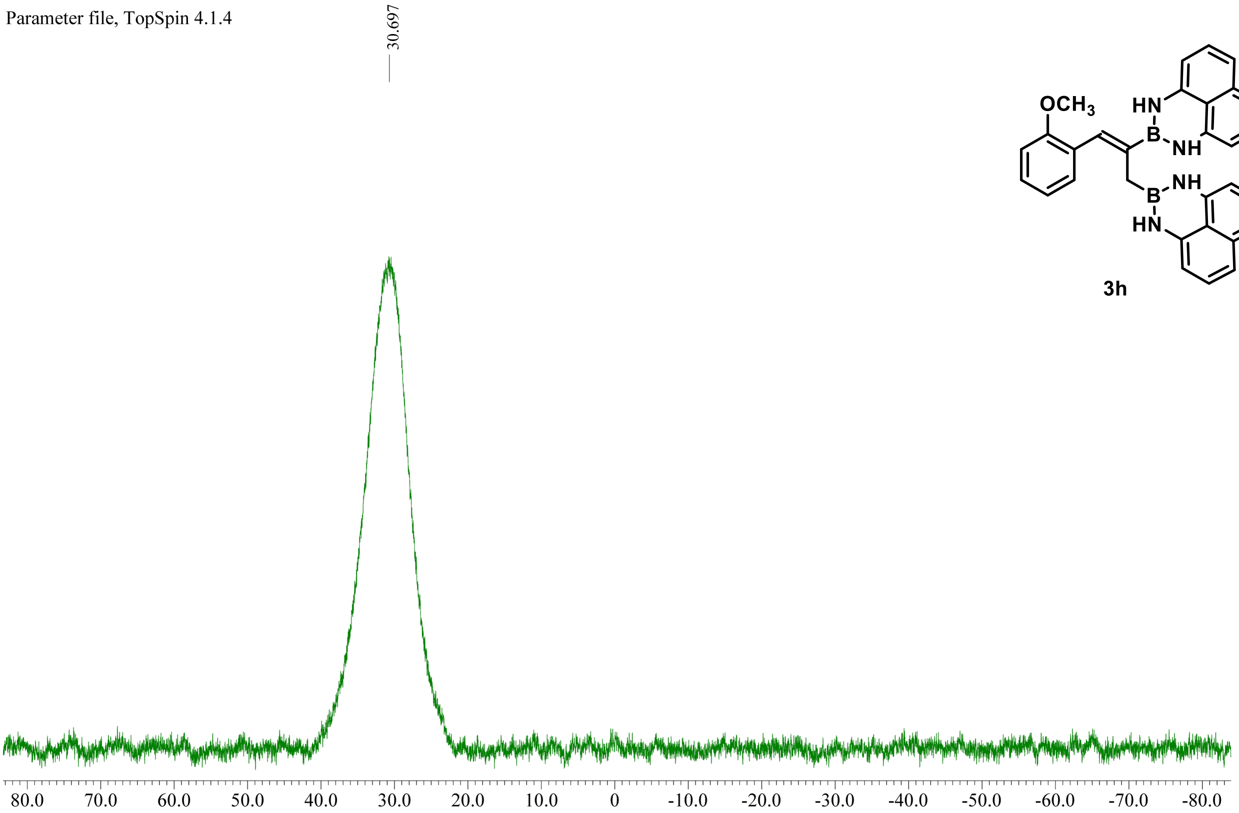

Figure S30. $^{11}B\{^{1}H\}$ NMR ($CDCl_3$, 128 MHz) spectrum of **3h**

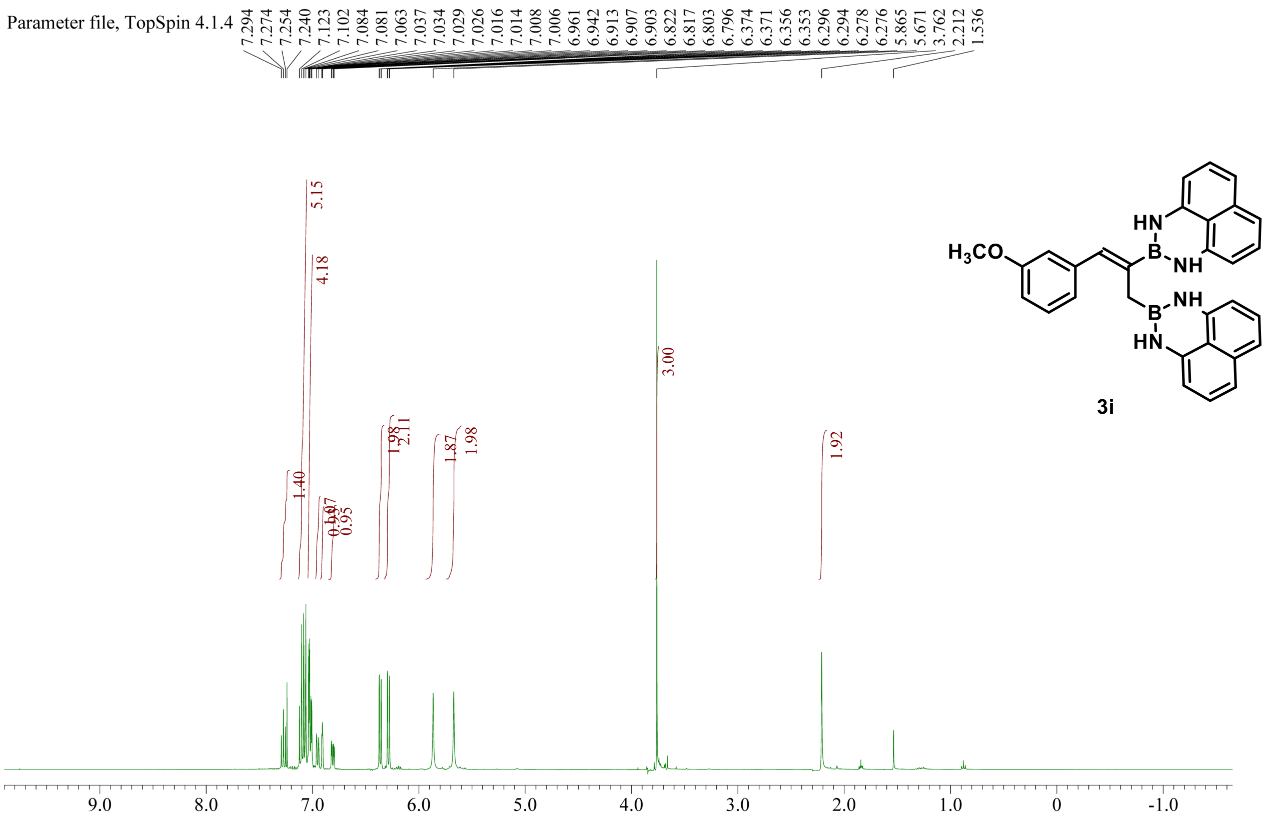

Figure S31. ^{1}H NMR ($CDCl_3$, 400 MHz) spectrum of **3i**

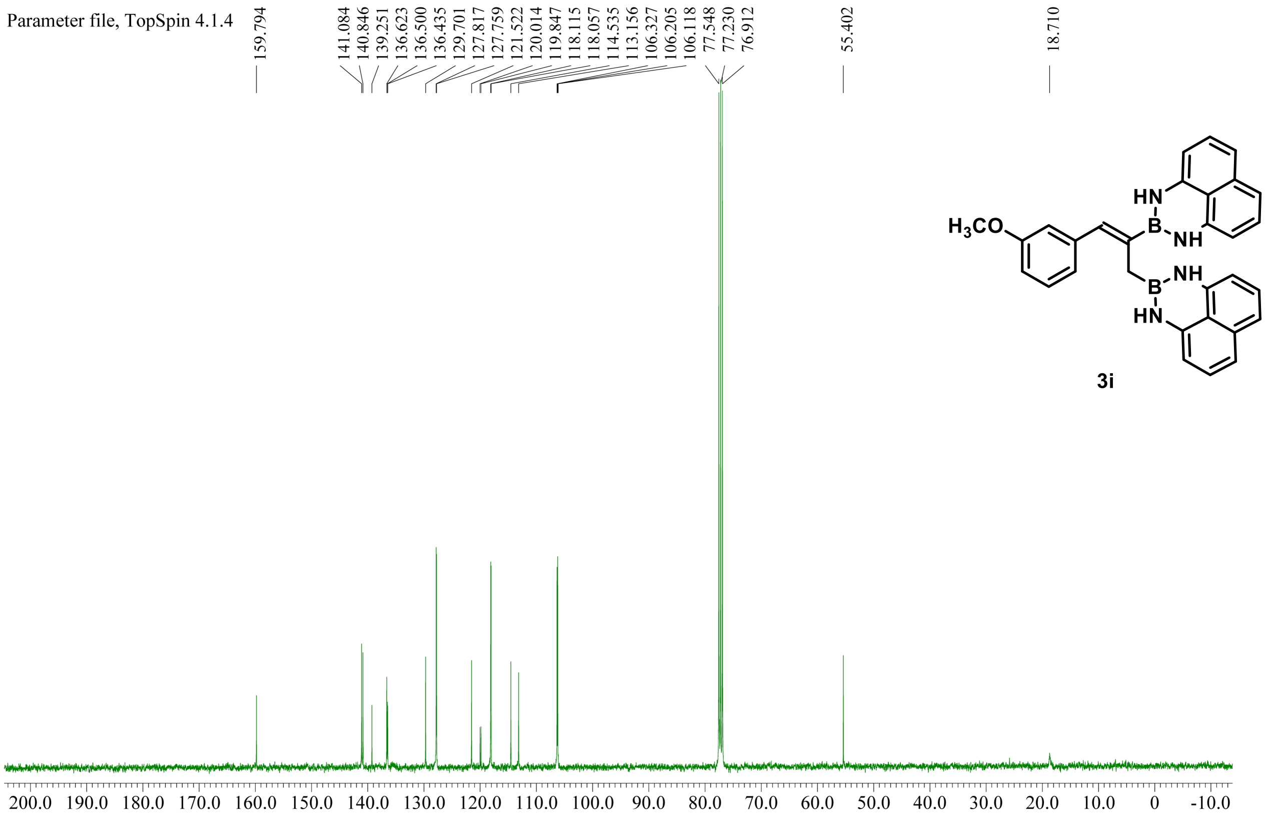

Figure S32. $^{13}C\{^{1}H\}$ NMR ($CDCl_3$, 100 MHz) spectrum of **3i**

Parameter file, TopSpin 4.1.4

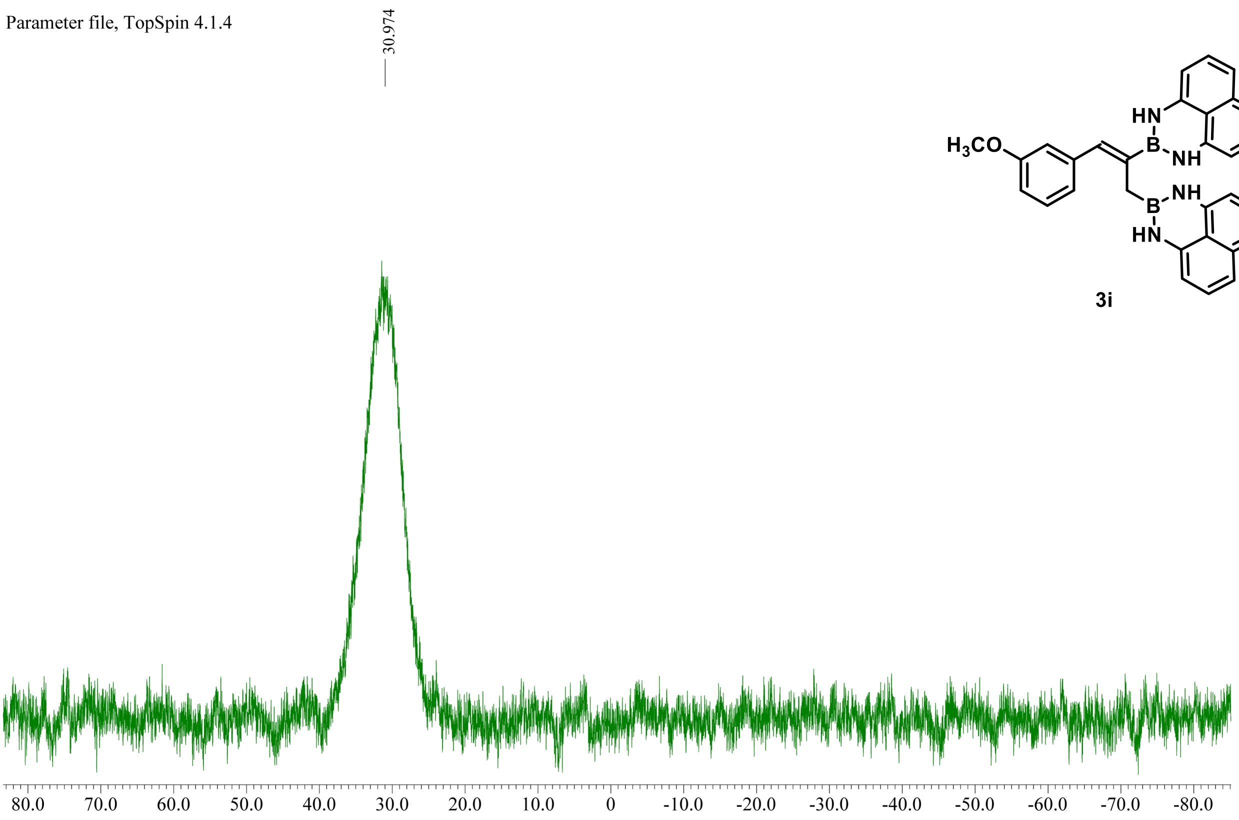

Figure S33. $^{11}B\{^{1}H\}$ NMR ($CDCl_3$, 128 MHz) spectrum of **3i**

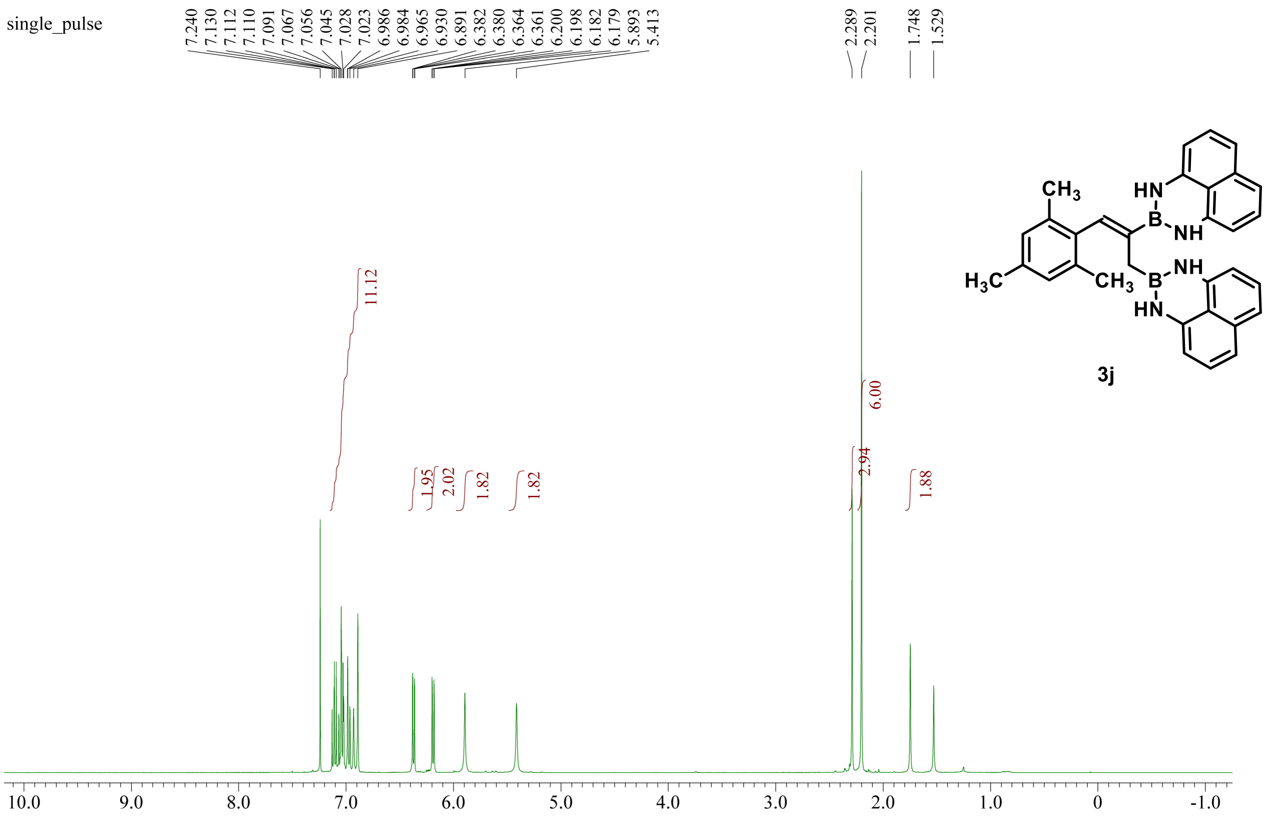

Figure S34. ^{1}H NMR ($CDCl_3$, 400 MHz) spectrum of **3j**

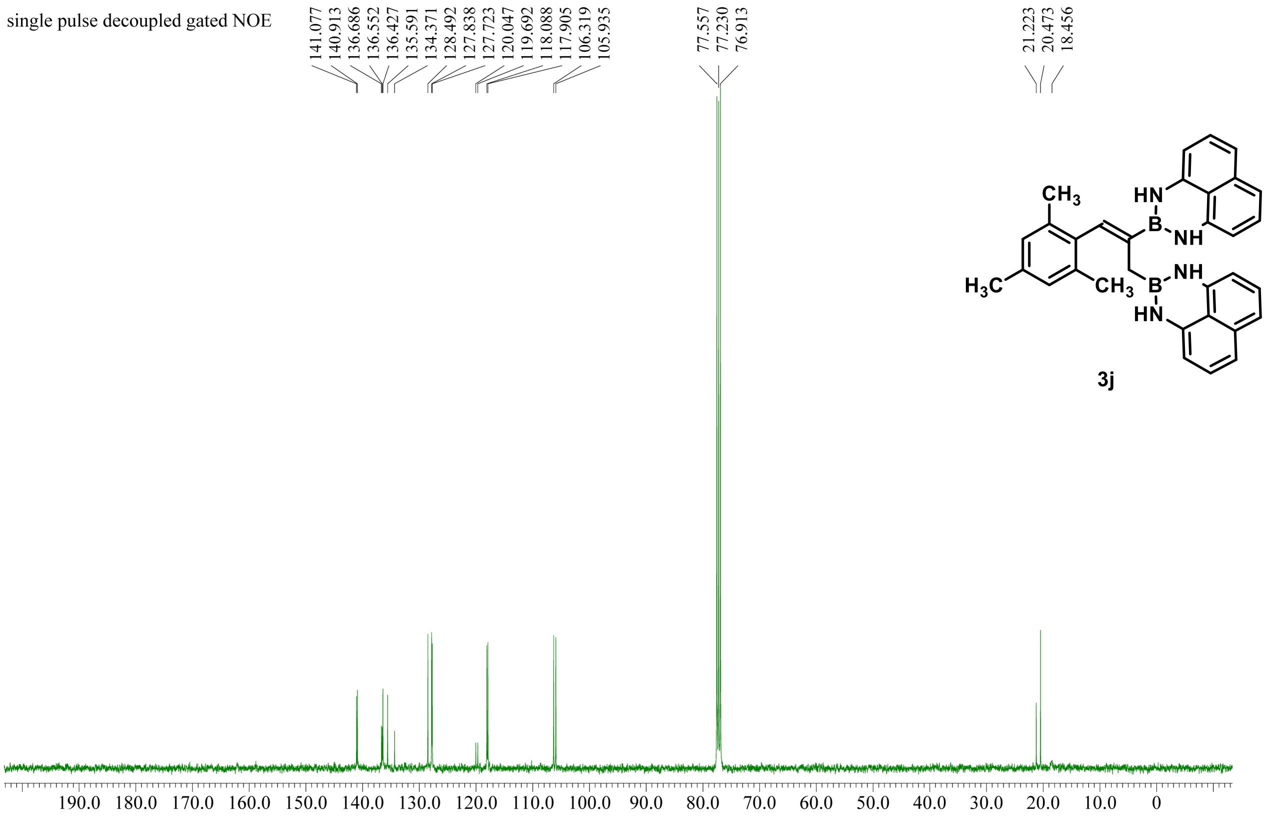

Figure S35. ^{13}C{^{1}H} NMR ($CDCl_3$, 100 MHz) spectrum of **3j**

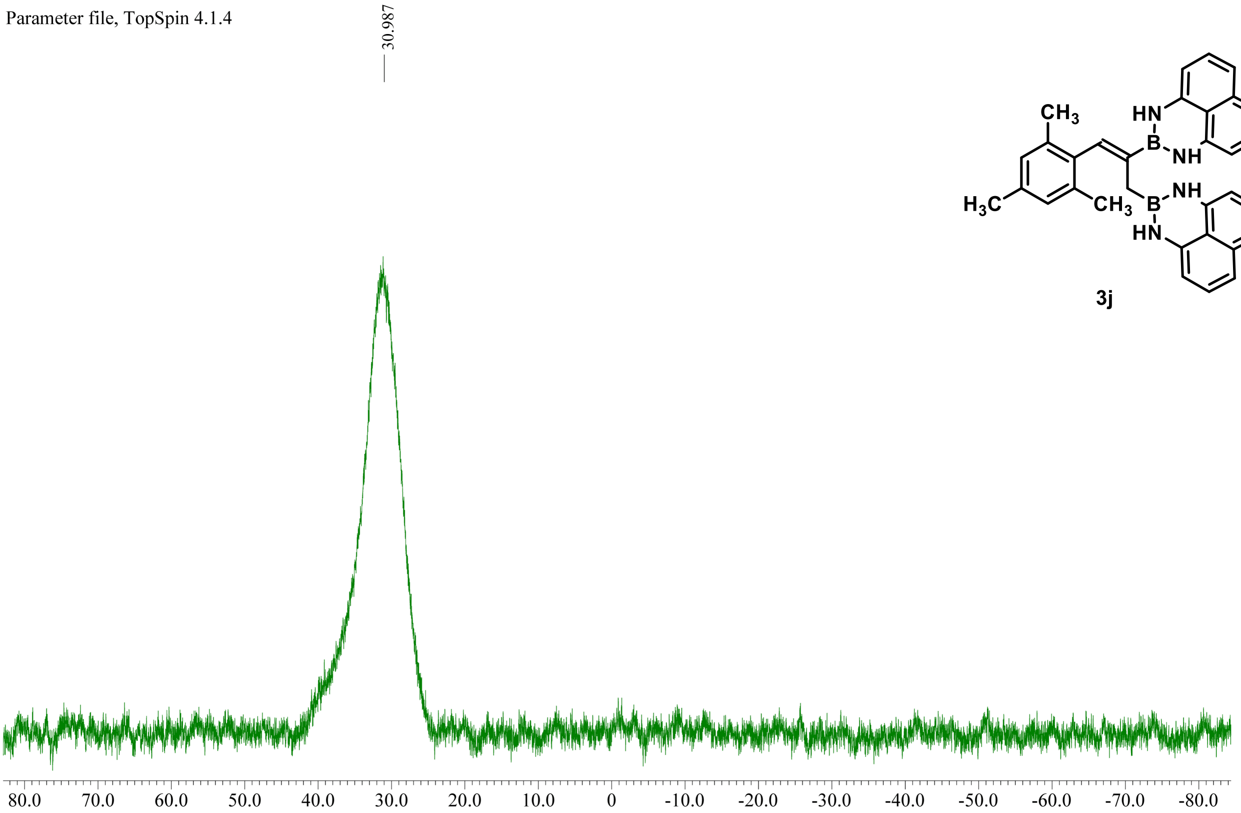

Figure S36. $^{11}B\{^1H\}$ NMR ($CDCl_3$, 128 MHz) spectrum of **3j**

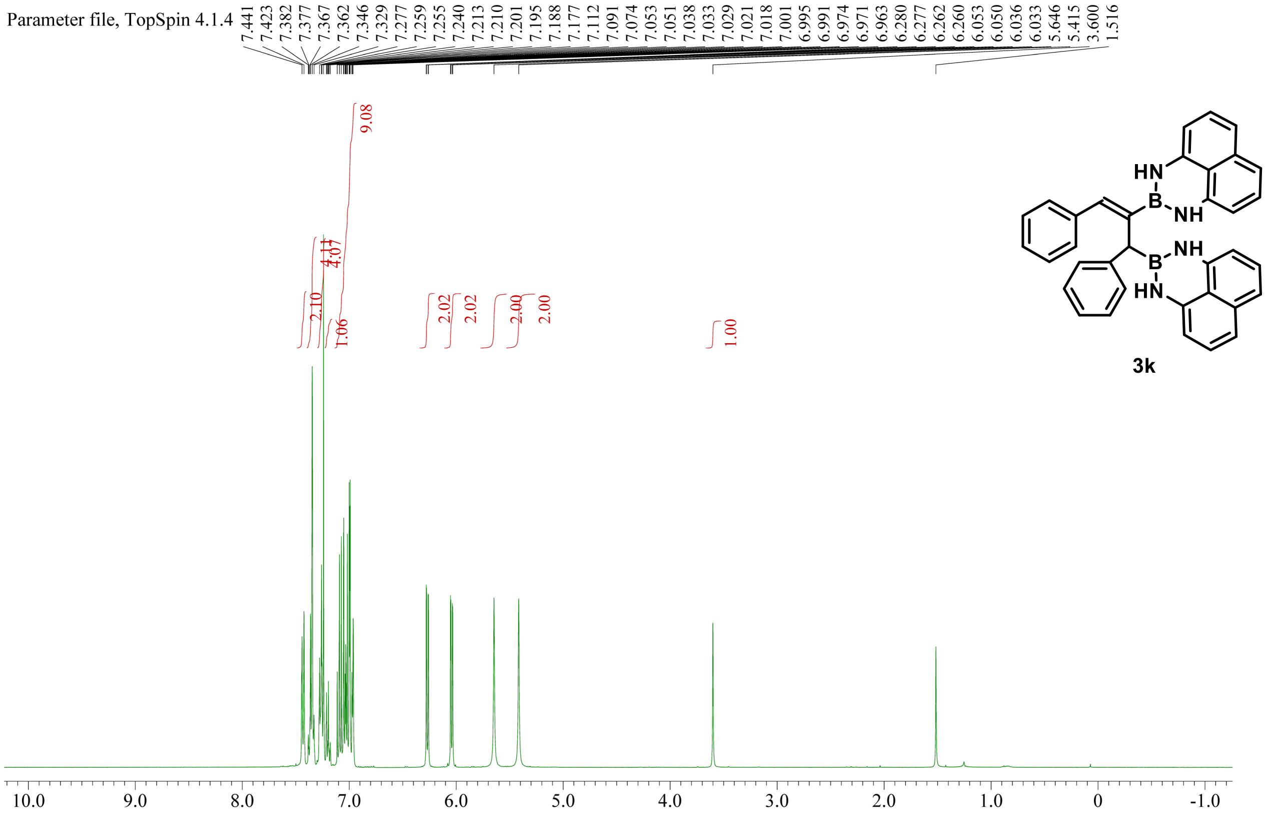

Figure S37. ^{1}H NMR ($CDCl_3$, 400 MHz) spectrum of **3k**

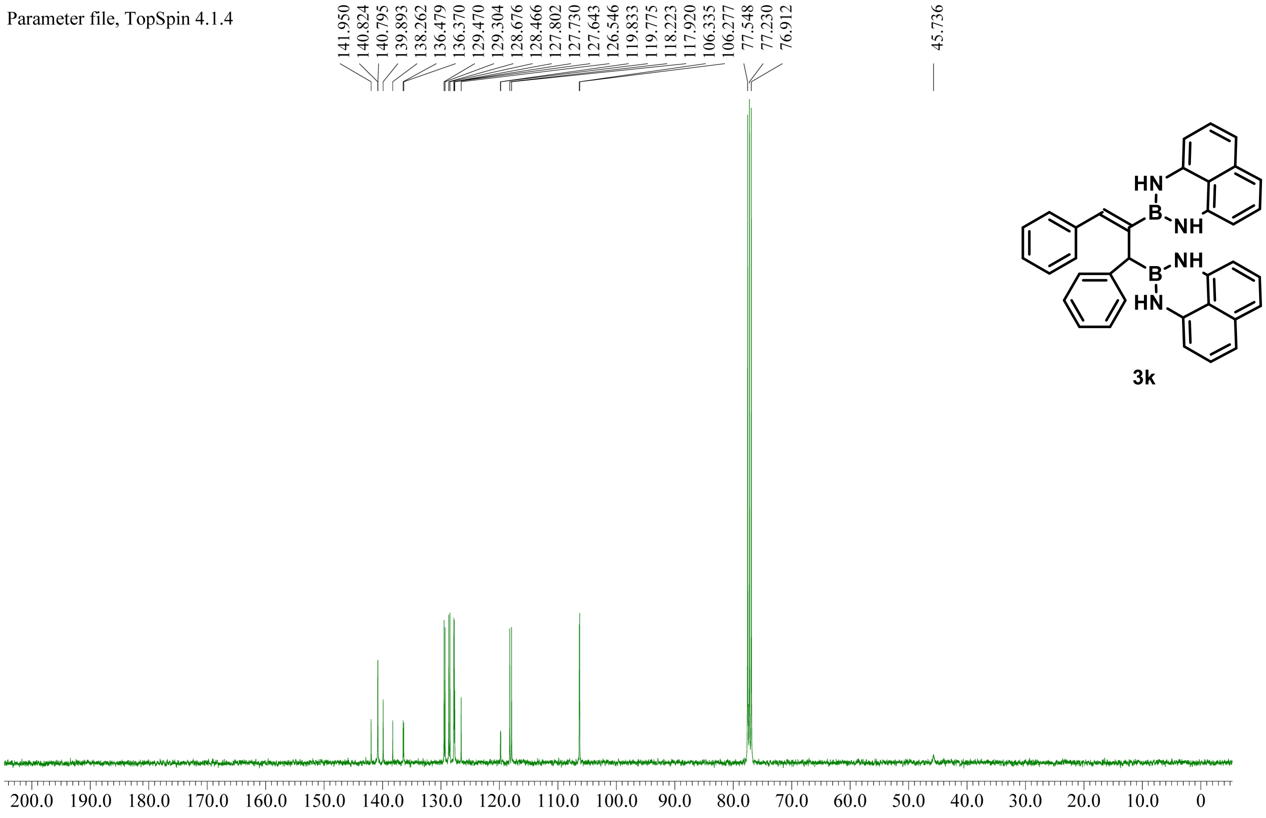

Figure S38. $^{13}C\{^{1}H\}$ NMR ($CDCl_3$, 100 MHz) spectrum of **3k**

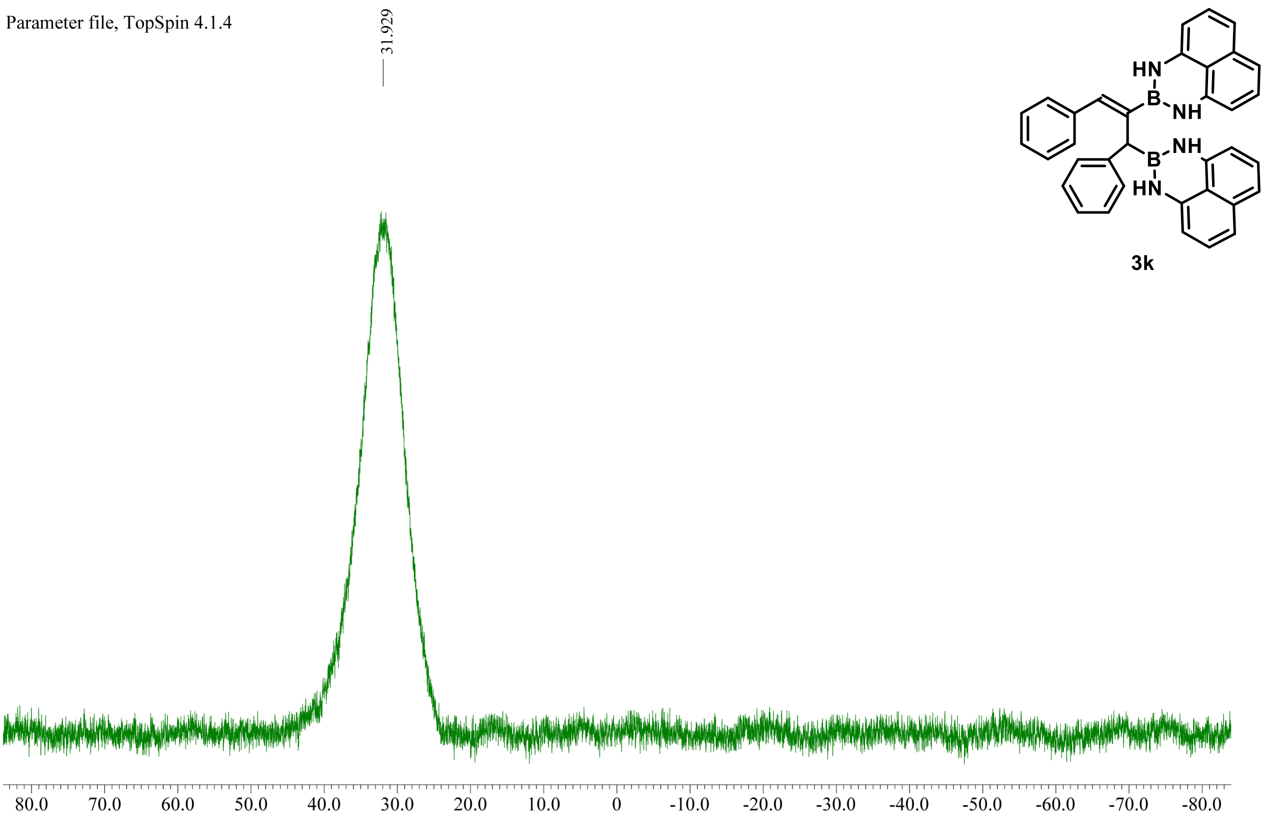

Figure S39. $^{11}B\{^{1}H\}$ NMR ($CDCl_3$, 128 MHz) spectrum of **3k**

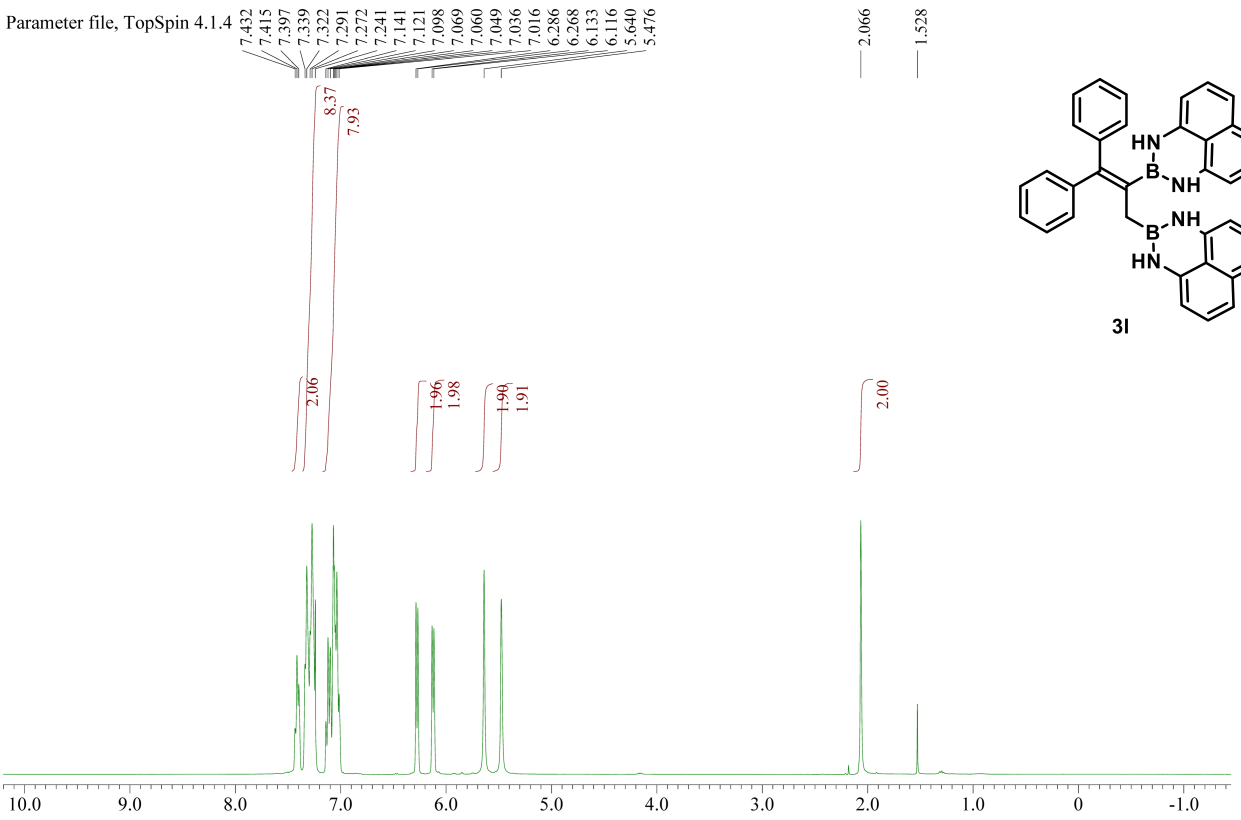

Figure S40. ^{1}H NMR ($CDCl_3$, 400 MHz) spectrum of **3l**

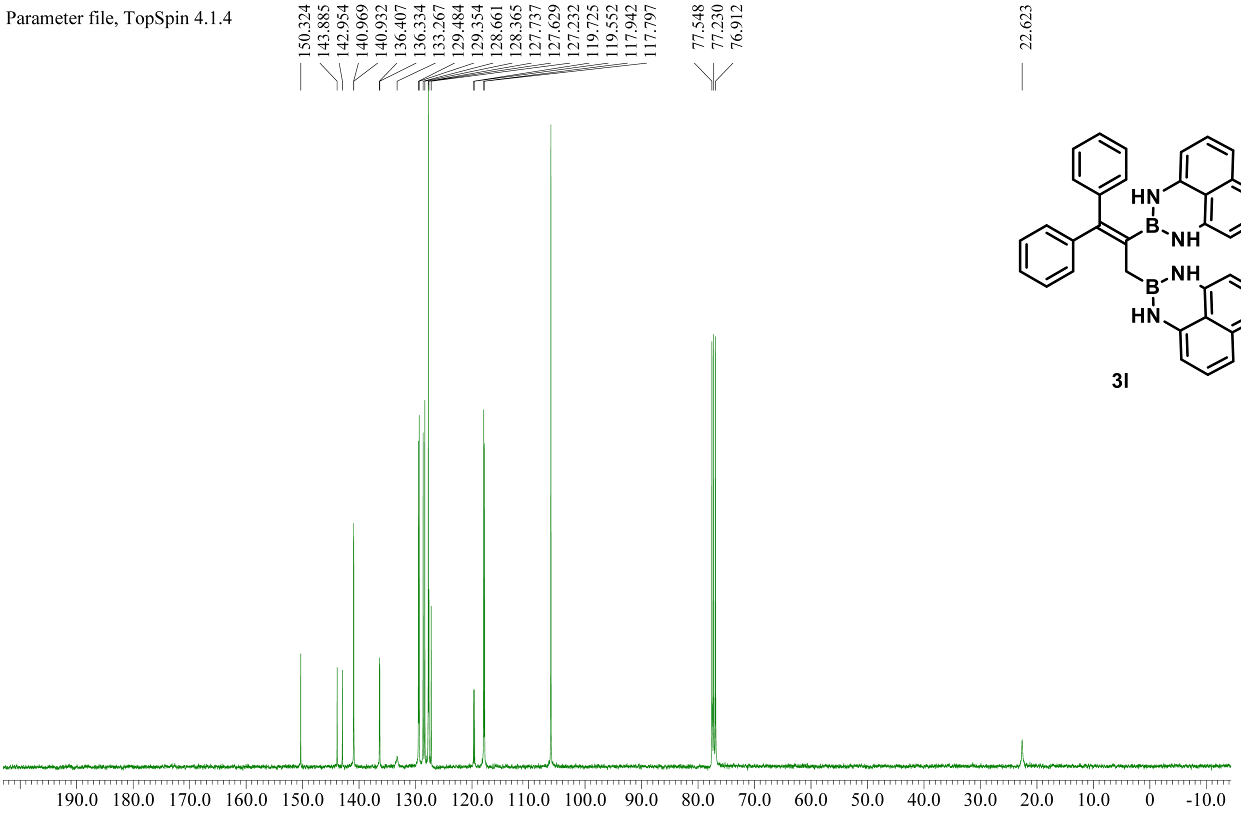

Figure S41. $^{13}C\{^{1}H\}$ NMR ($CDCl_3$, 100 MHz) spectrum of **3l**

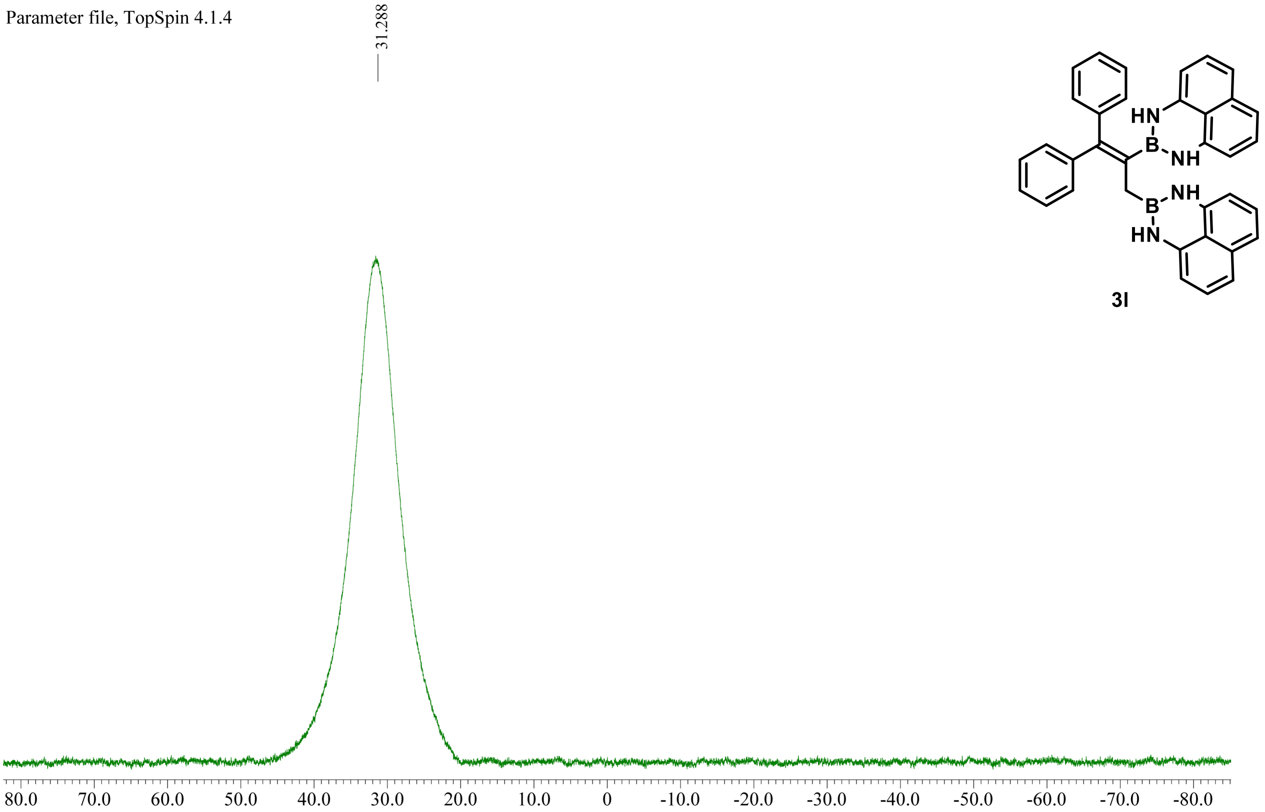

Figure S42. $^{11}B\{^1H\}$ NMR ($CDCl_3$, 128 MHz) spectrum of **3l**

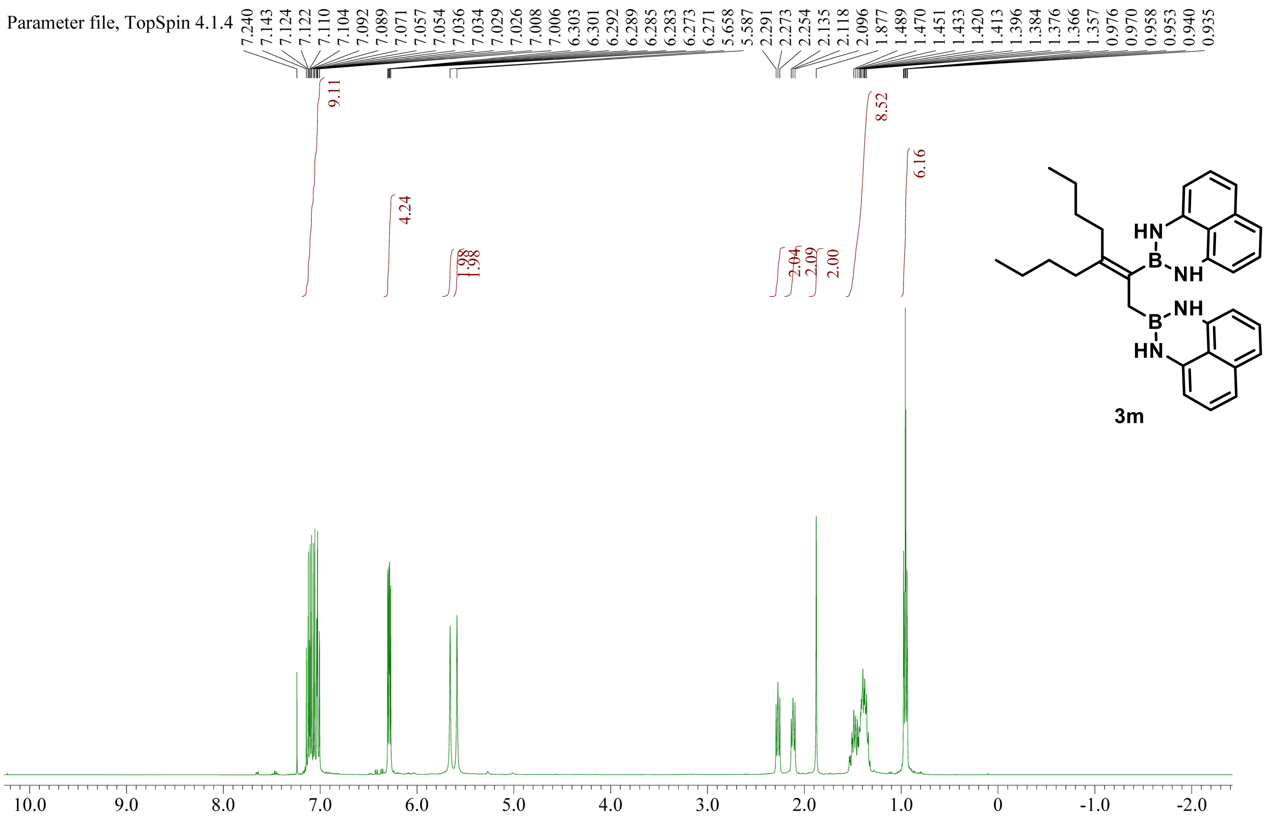

Figure S43. ^{1}H NMR ($CDCl_3$, 400 MHz) spectrum of **3m**

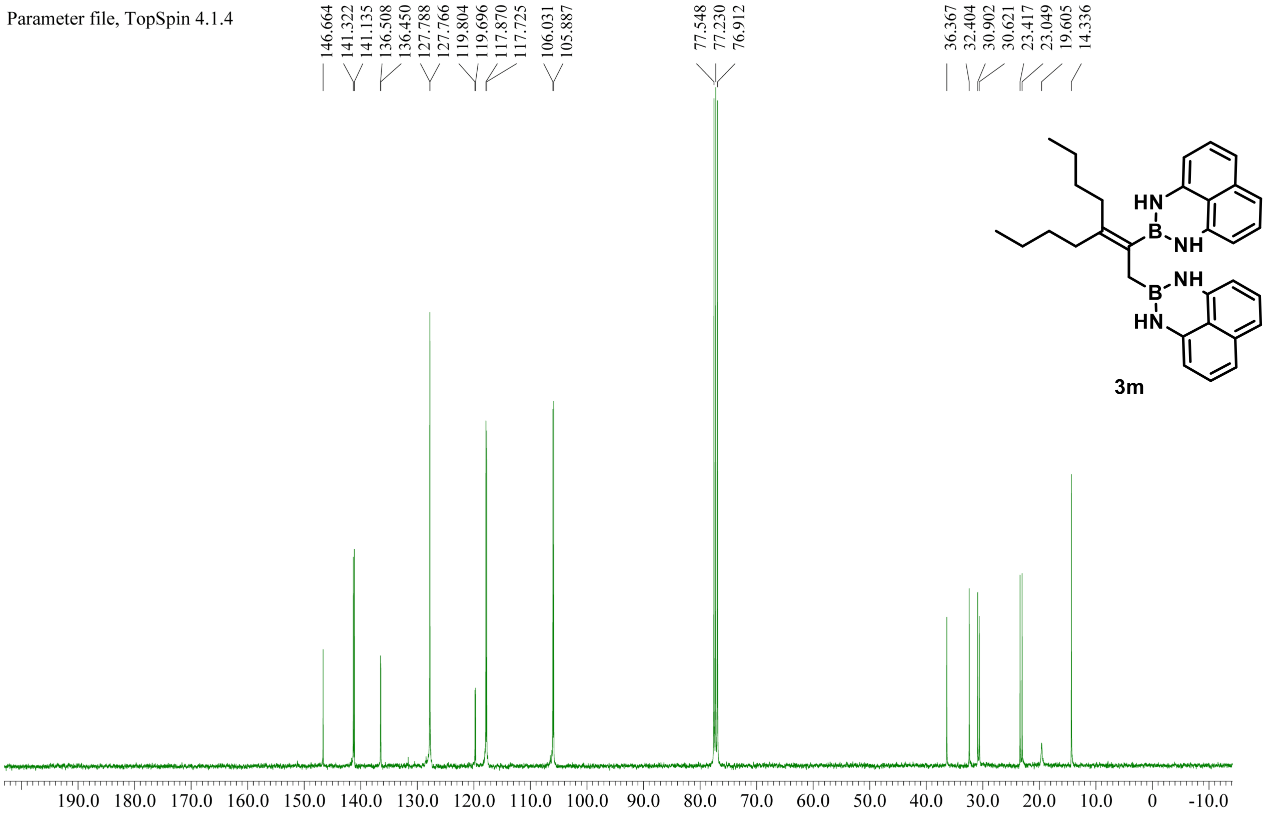

Figure S44. $^{13}C\{^{1}H\}$ NMR ($CDCl_3$, 100 MHz) spectrum of **3m**

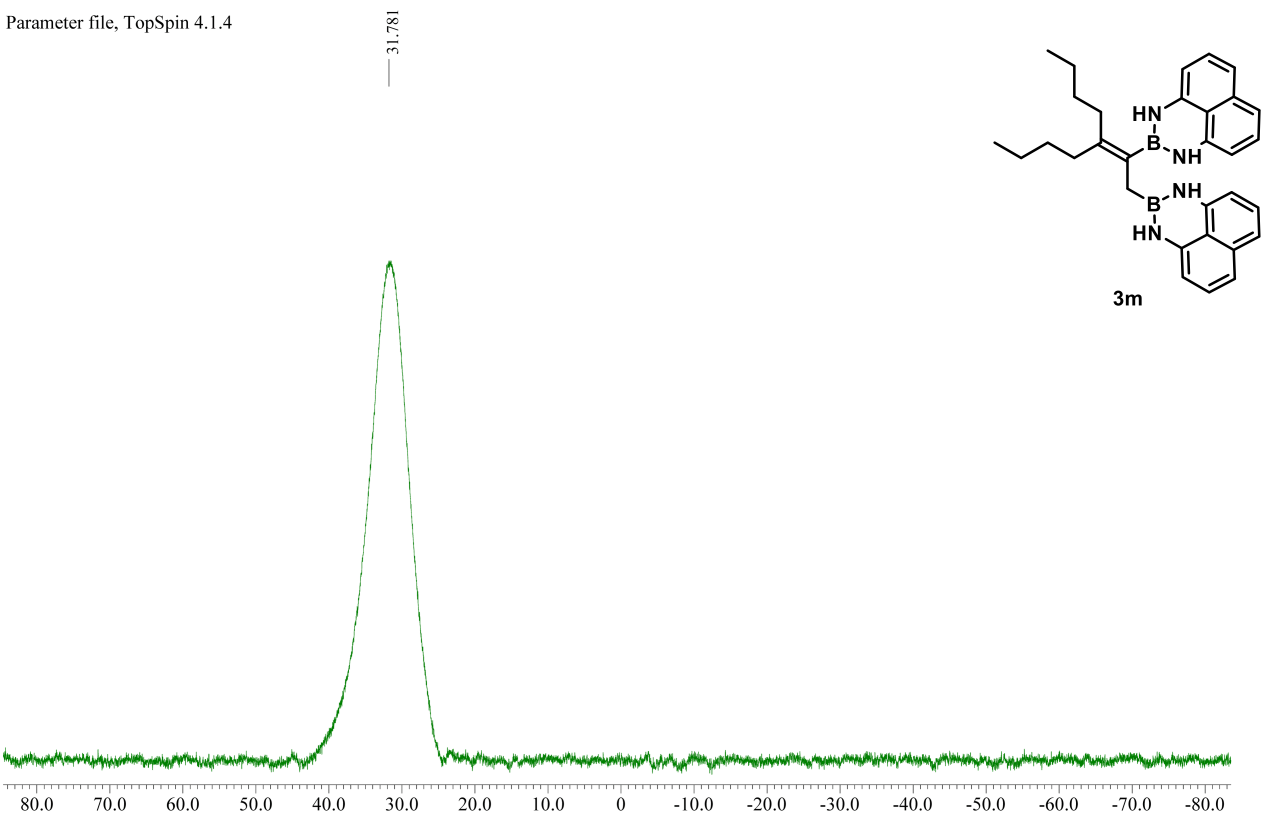

Figure S45. $^{11}B\{^{1}H\}$ NMR ($CDCl_3$, 128 MHz) spectrum of **3m**

Parameter file, TopSpin 4.1.4

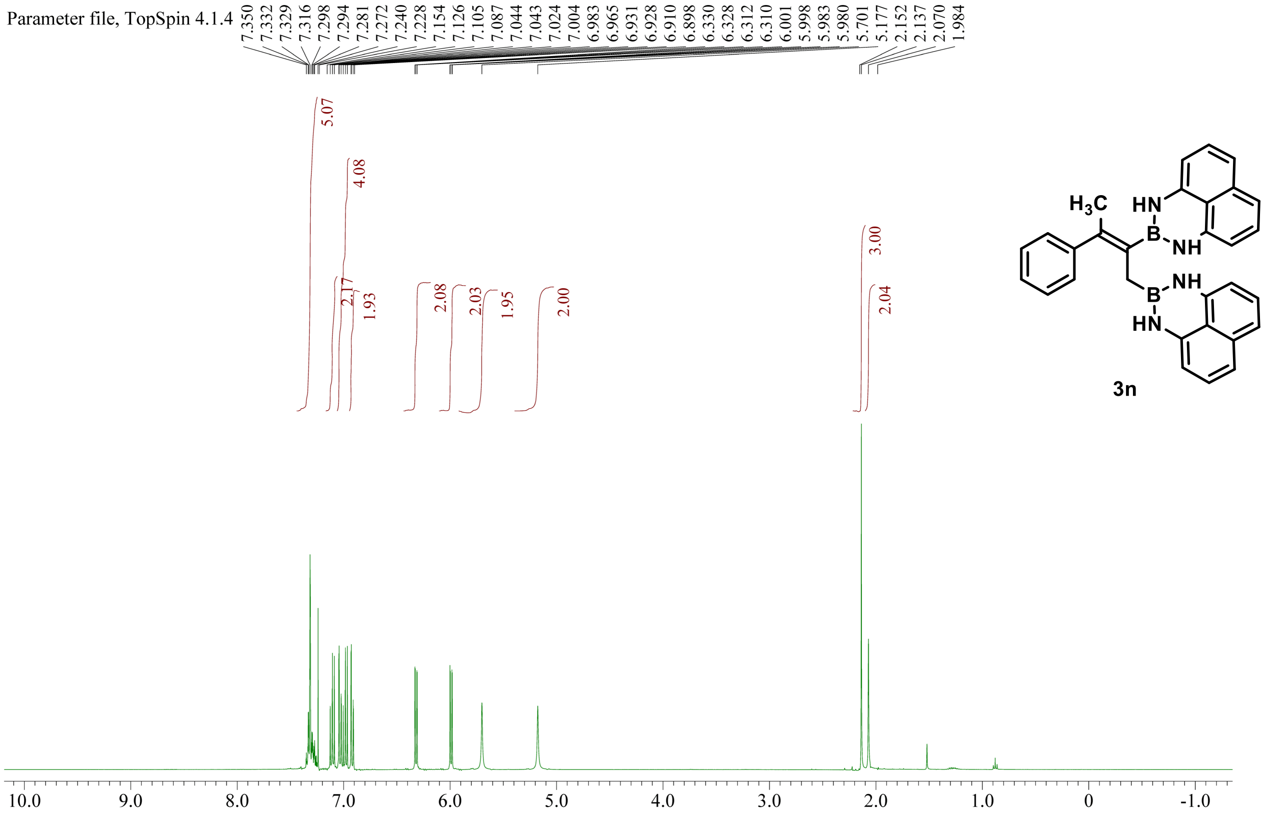

Figure S46. ^{1}H NMR ($CDCl_3$, 400 MHz) spectrum of **3n**

Parameter file, TopSpin 4.1.4

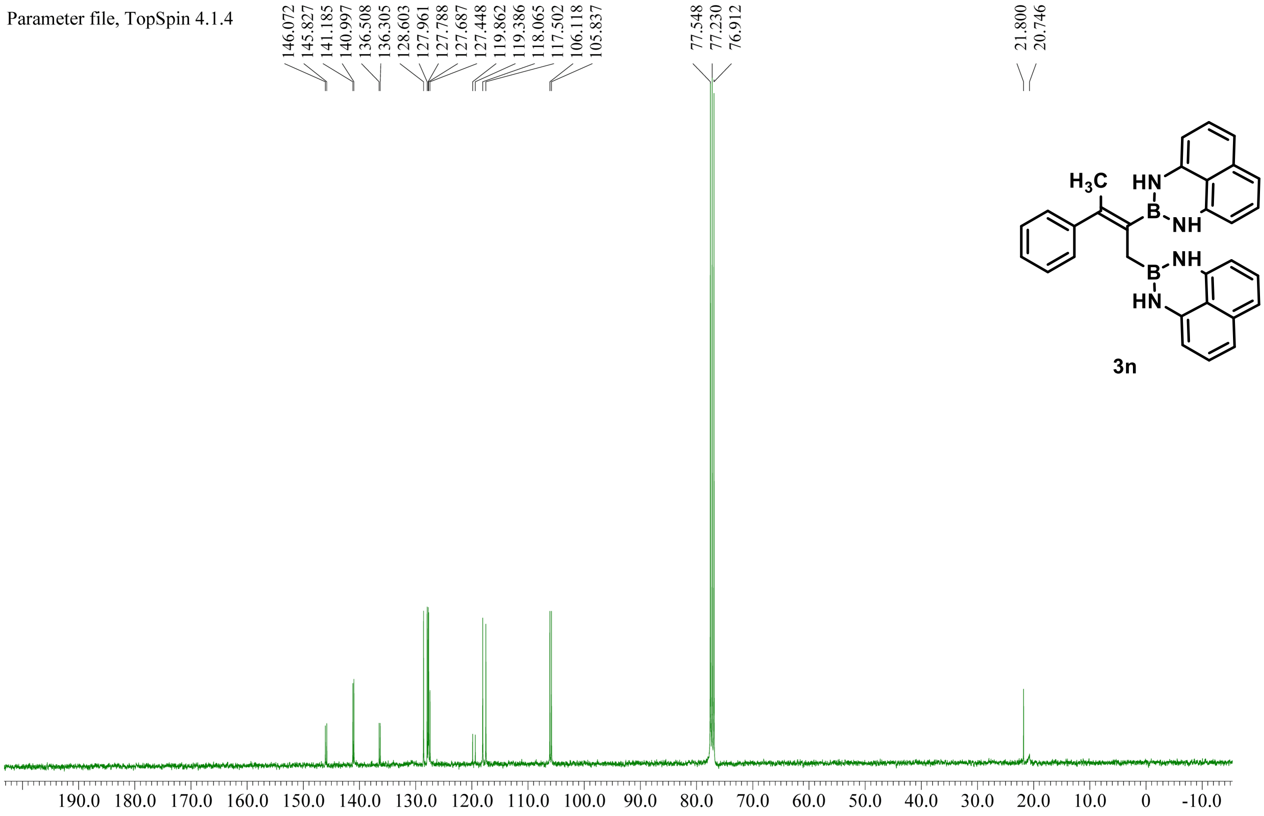

Figure S47. $^{13}C\{^{1}H\}$ NMR ($CDCl_3$, 100 MHz) spectrum of **3n**

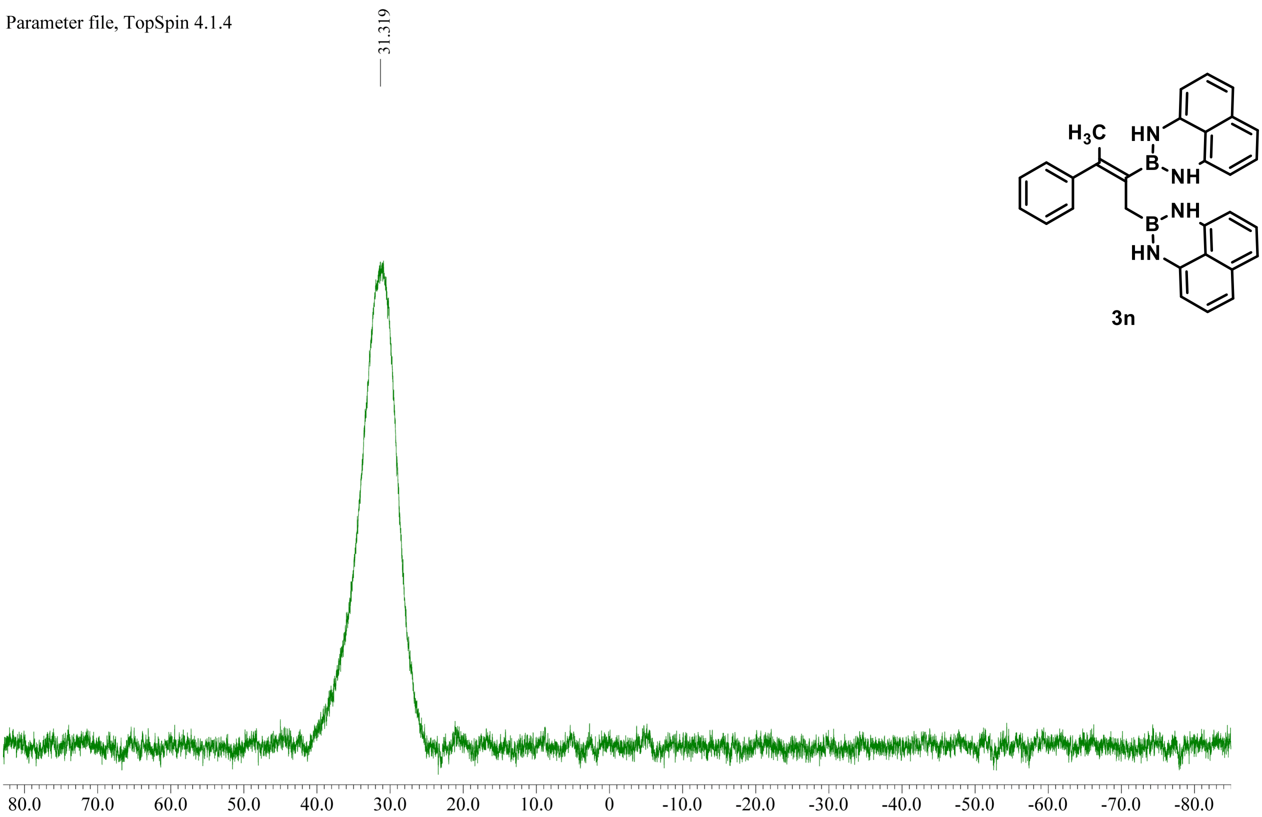

3n

Figure S48. $^{11}B\{^{1}H\}$ NMR ($CDCl_3$, 128 MHz) spectrum of **3n**

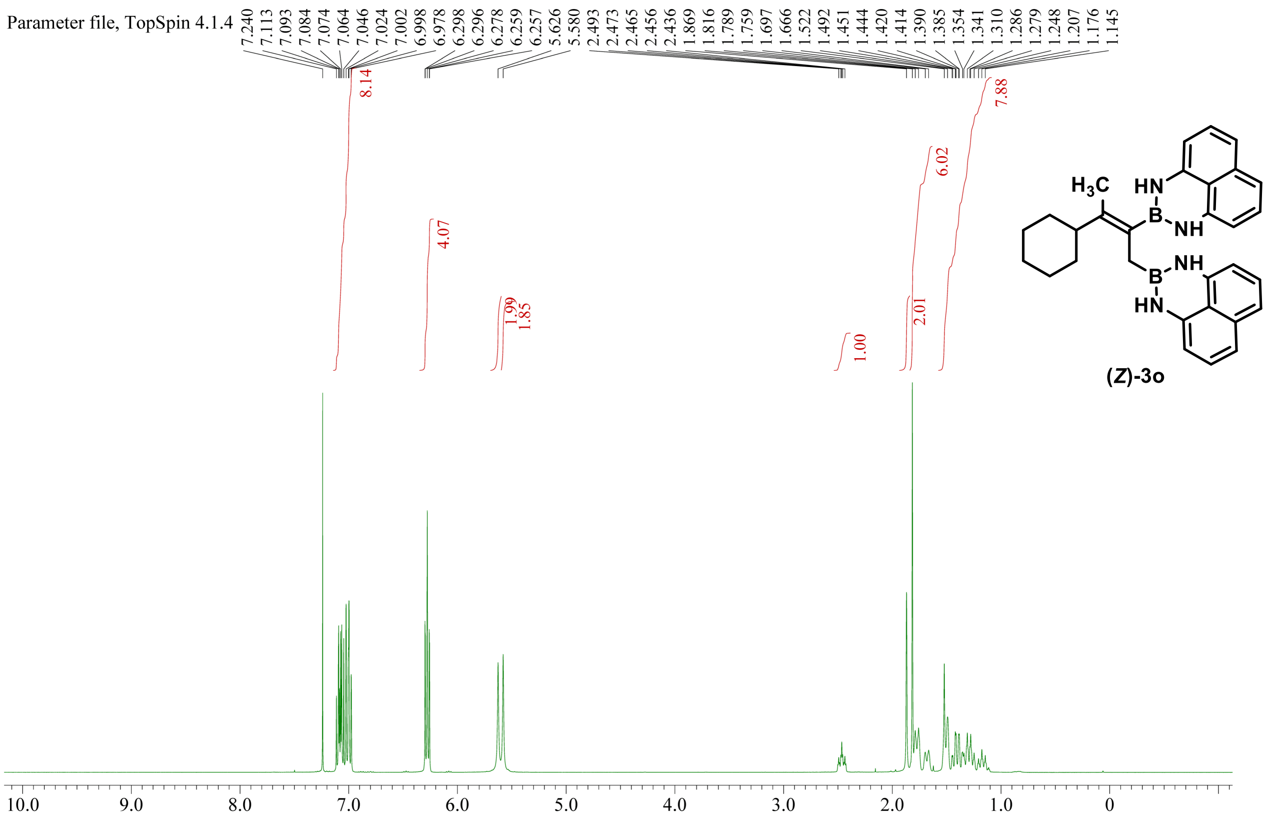

Figure S49. ^{1}H NMR ($CDCl_3$, 400 MHz) spectrum of ***(Z)*-3o**

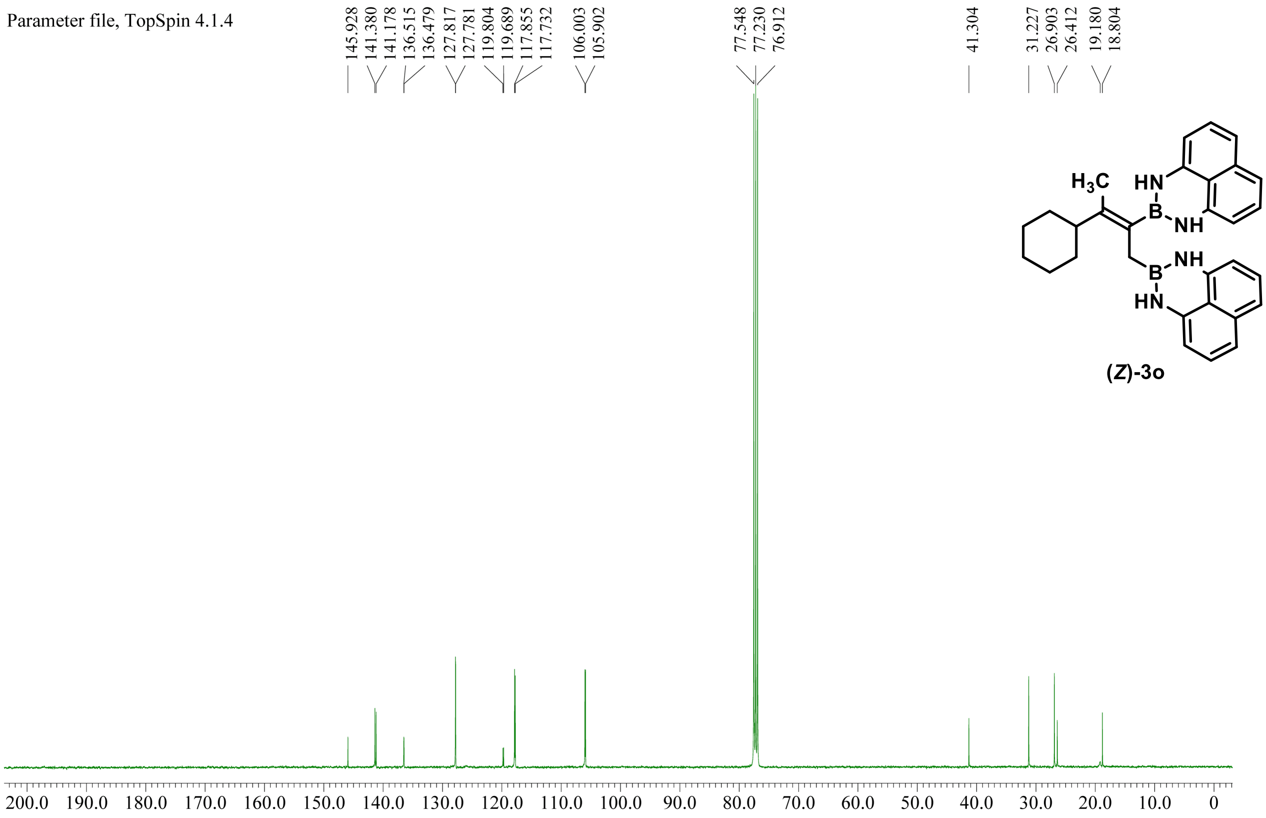

Figure S50. $^{13}C\{^{1}H\}$ NMR ($CDCl_3$, 100 MHz) spectrum of ***(Z)*-3o**

Parameter file, TopSpin 4.1.4

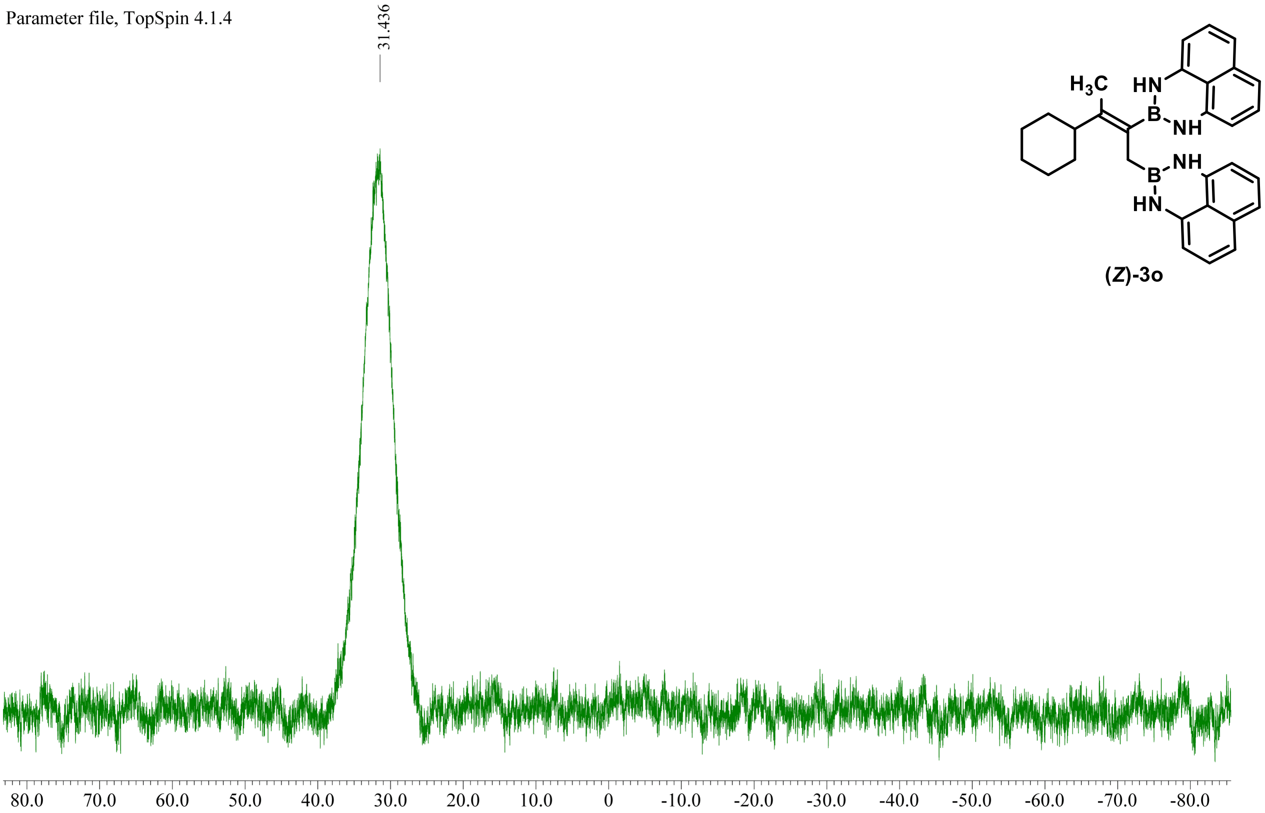

(*Z*)-3o

Figure S51. $^{11}B\{^{1}H\}$ NMR ($CDCl_3$, 128 MHz) spectrum of **(*Z*)-3o**

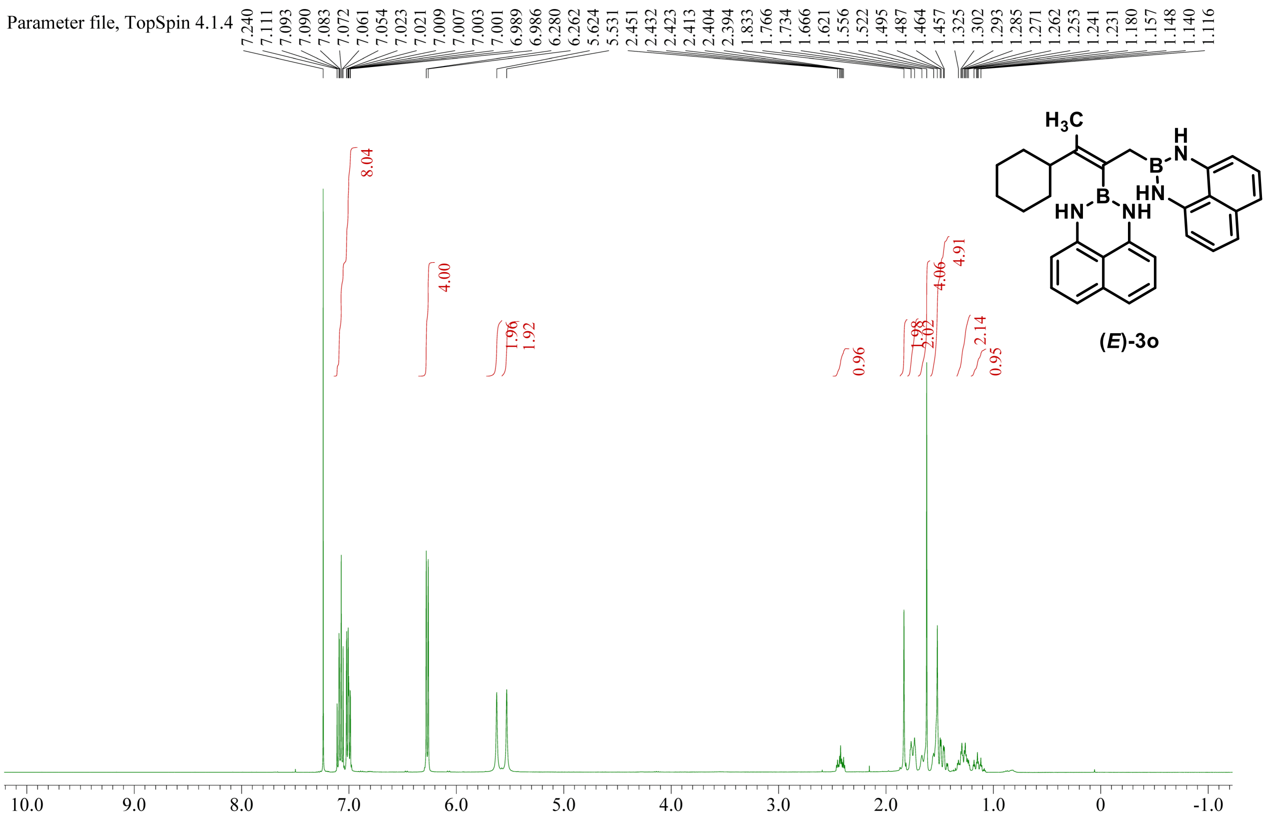

Figure S52. ^{1}H NMR ($CDCl_3$, 400 MHz) spectrum of ***(E)*-3o**

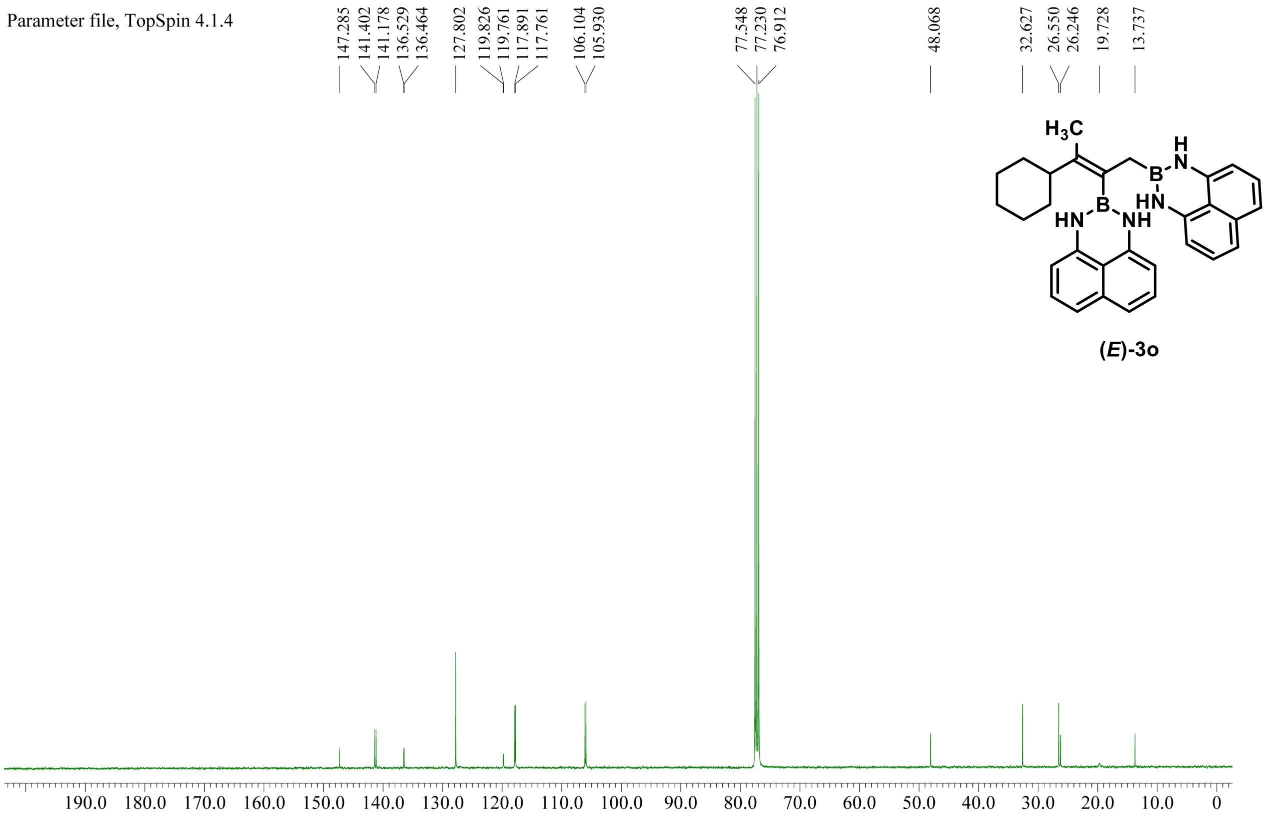

Figure S53. ^{13}C{^{1}H} NMR ($CDCl_3$, 100 MHz) spectrum of ***(E)*-3o**

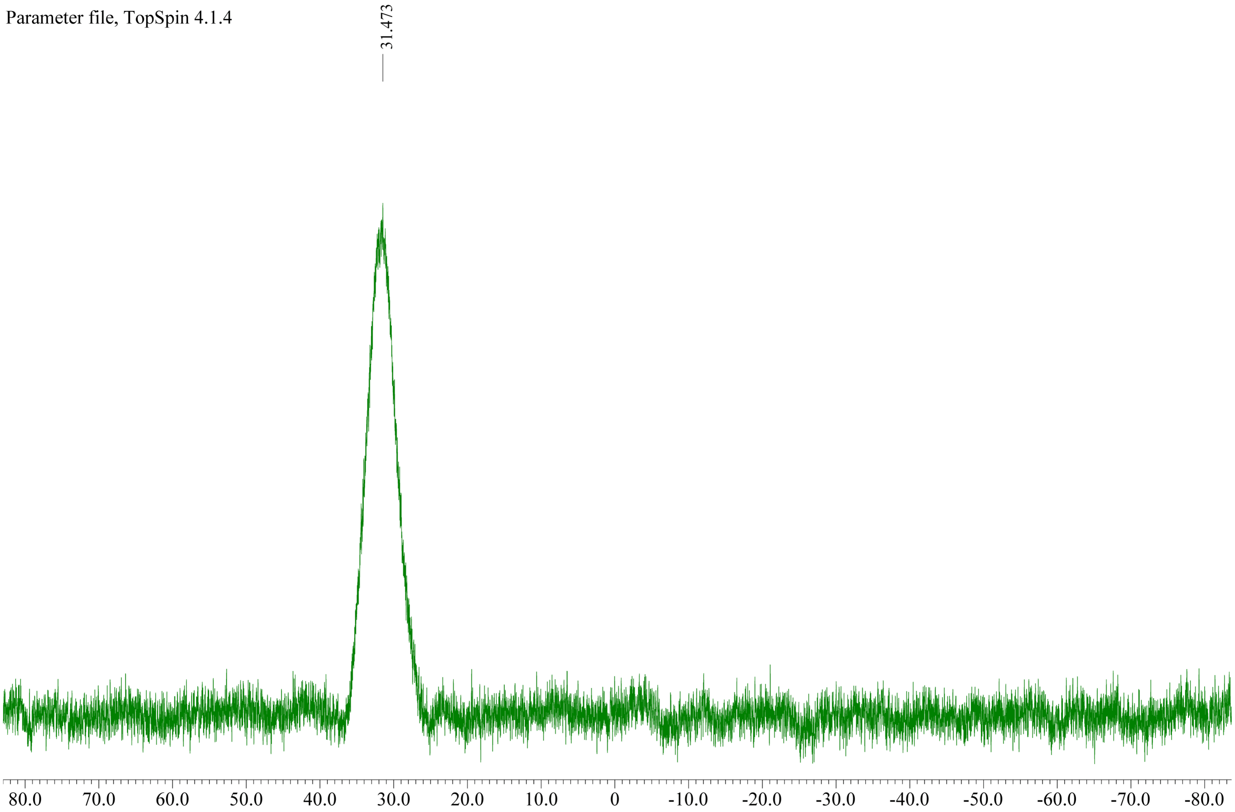

Figure S54. $^{11}B\{^{1}H\}$ NMR ($CDCl_3$, 128 MHz) spectrum of **(*E*)-3o**

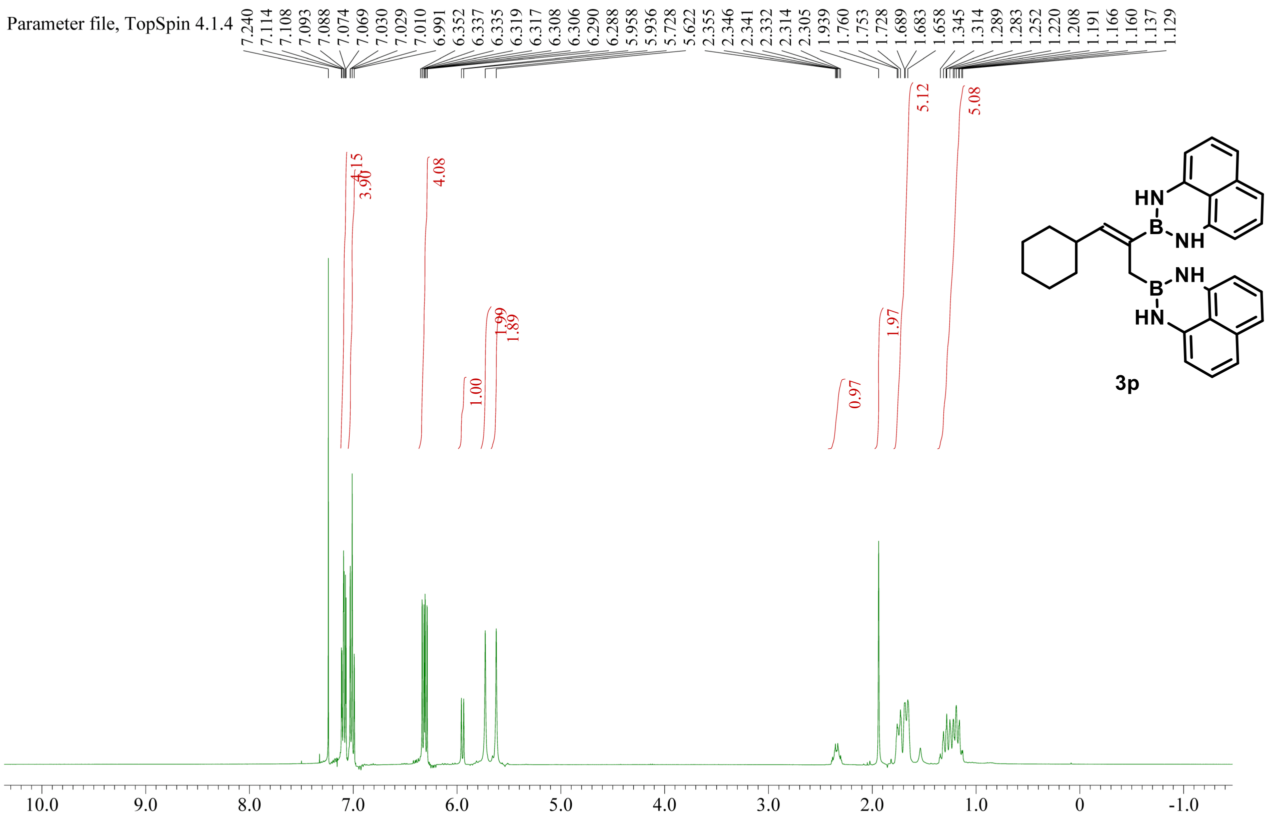

Figure S55. ^{1}H NMR ($CDCl_3$, 400 MHz) spectrum of **3p**

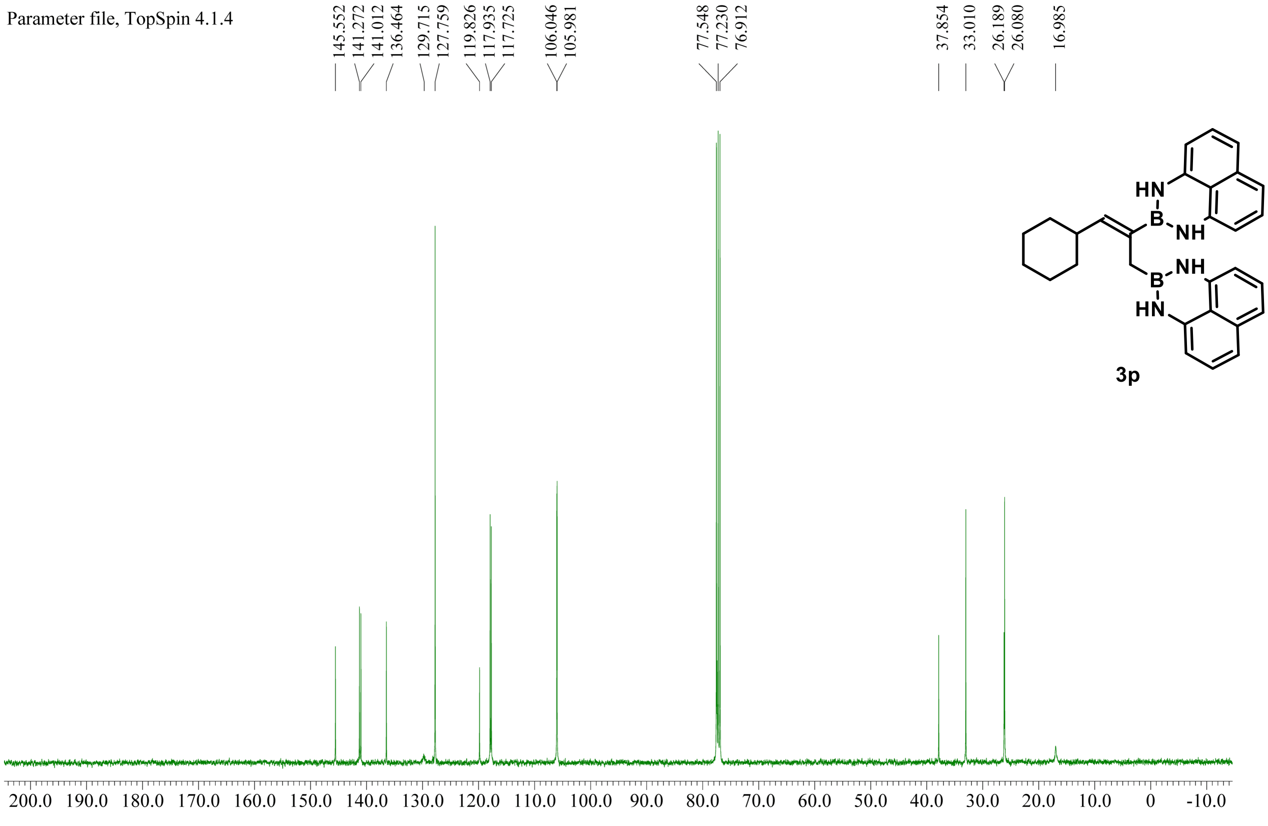

Figure S56. $^{13}C\{^{1}H\}$ NMR ($CDCl_3$, 100 MHz) spectrum of **3p**

Parameter file, TopSpin 4.1.4

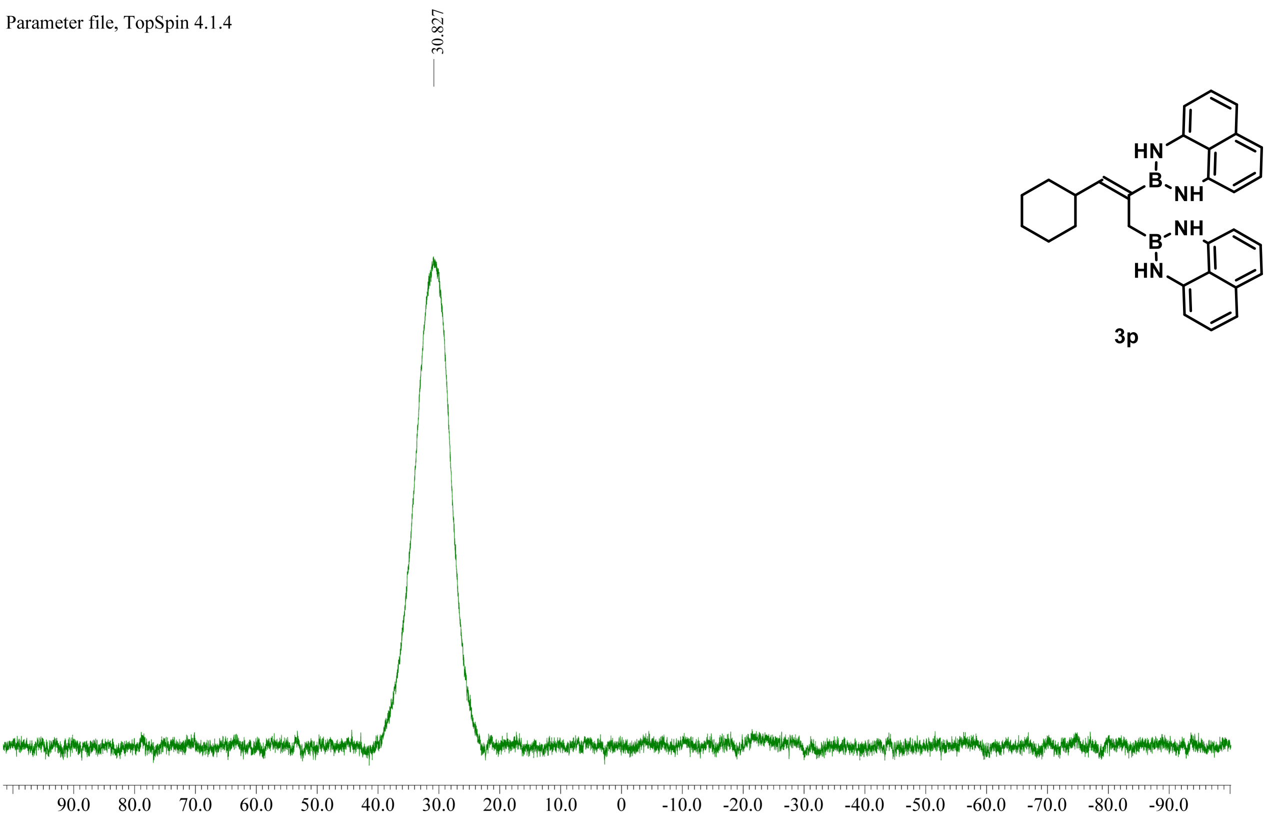

Figure S57. $^{11}B\{^{1}H\}$ NMR ($CDCl_3$, 128 MHz) spectrum of **3p**

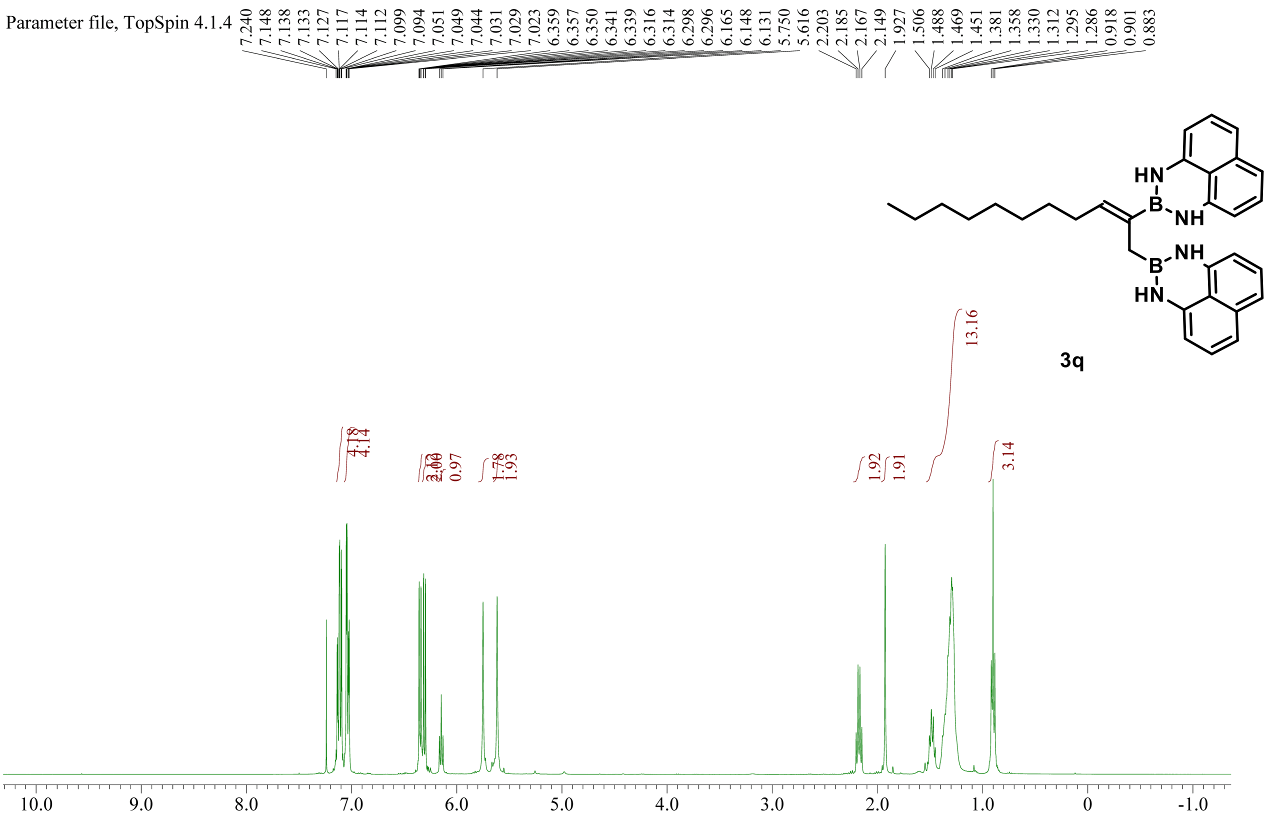

Figure S58. ^{1}H NMR ($CDCl_3$, 400 MHz) spectrum of **3q**

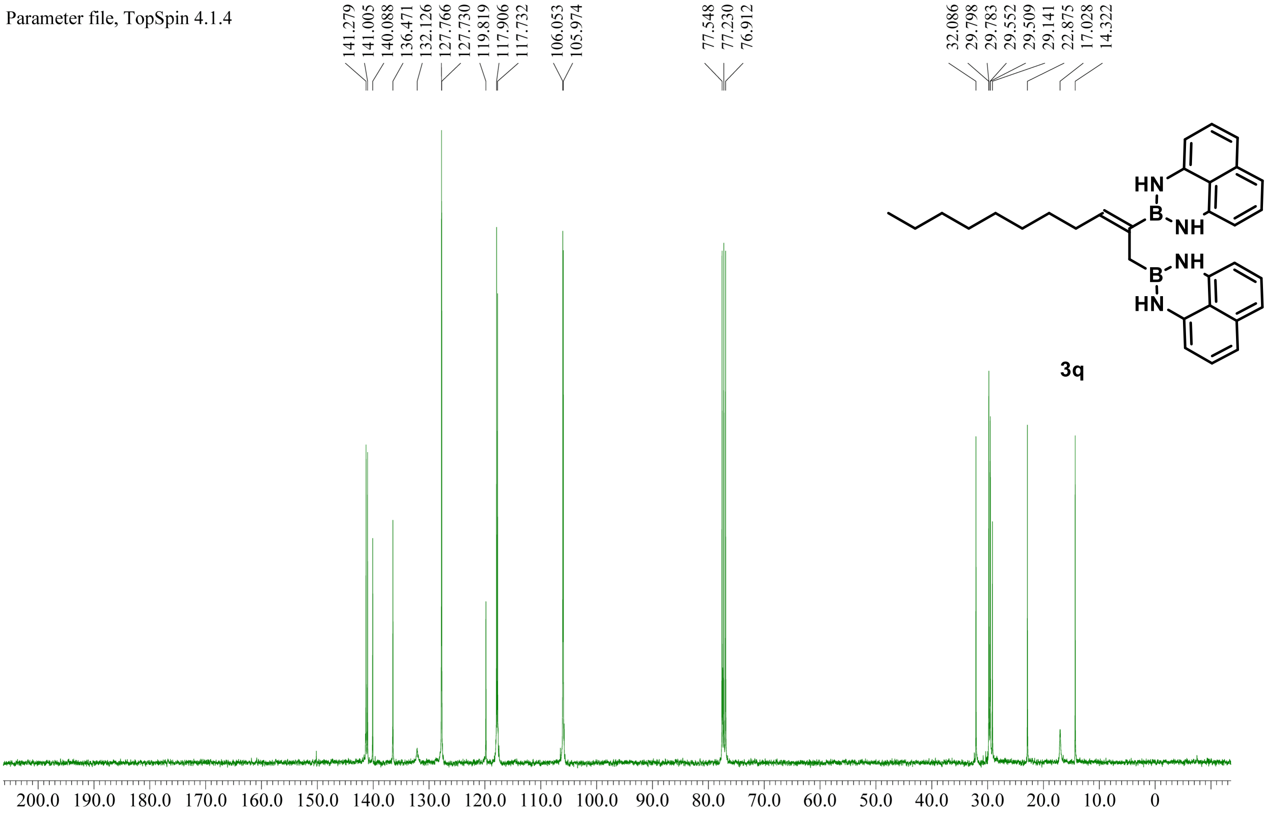

Figure S59. $^{13}C\{^{1}H\}$ NMR ($CDCl_3$, 100 MHz) spectrum of **3q**

Parameter file, TopSpin 4.1.4

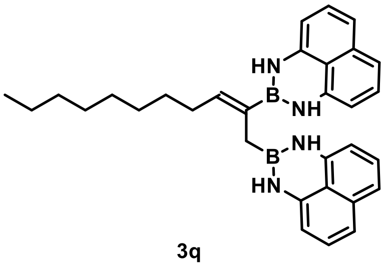

3q

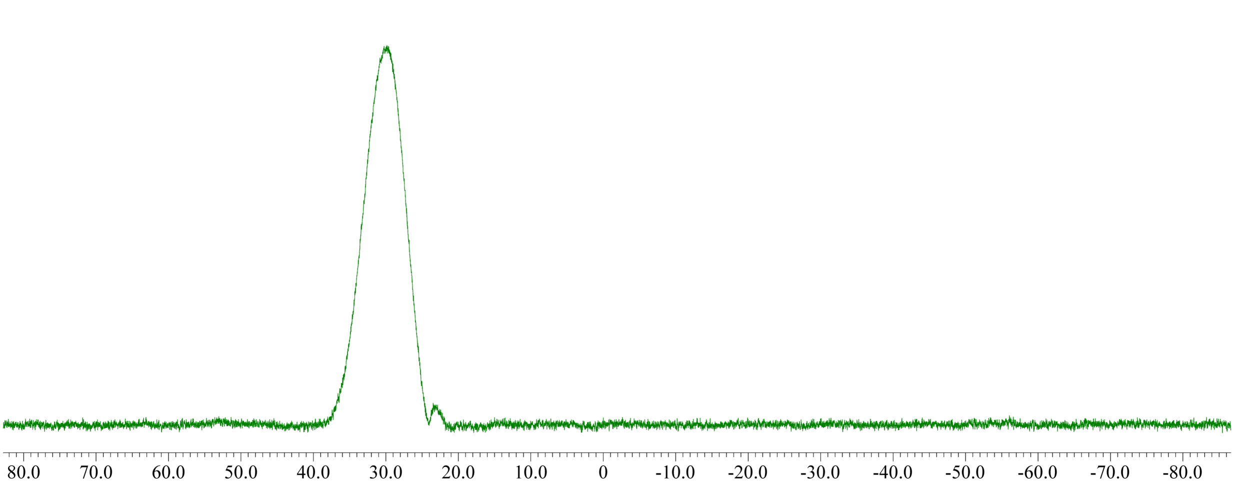

Figure S60. $^{11}B\{^{1}H\}$ NMR ($CDCl_3$, 128 MHz) spectrum of **3q**

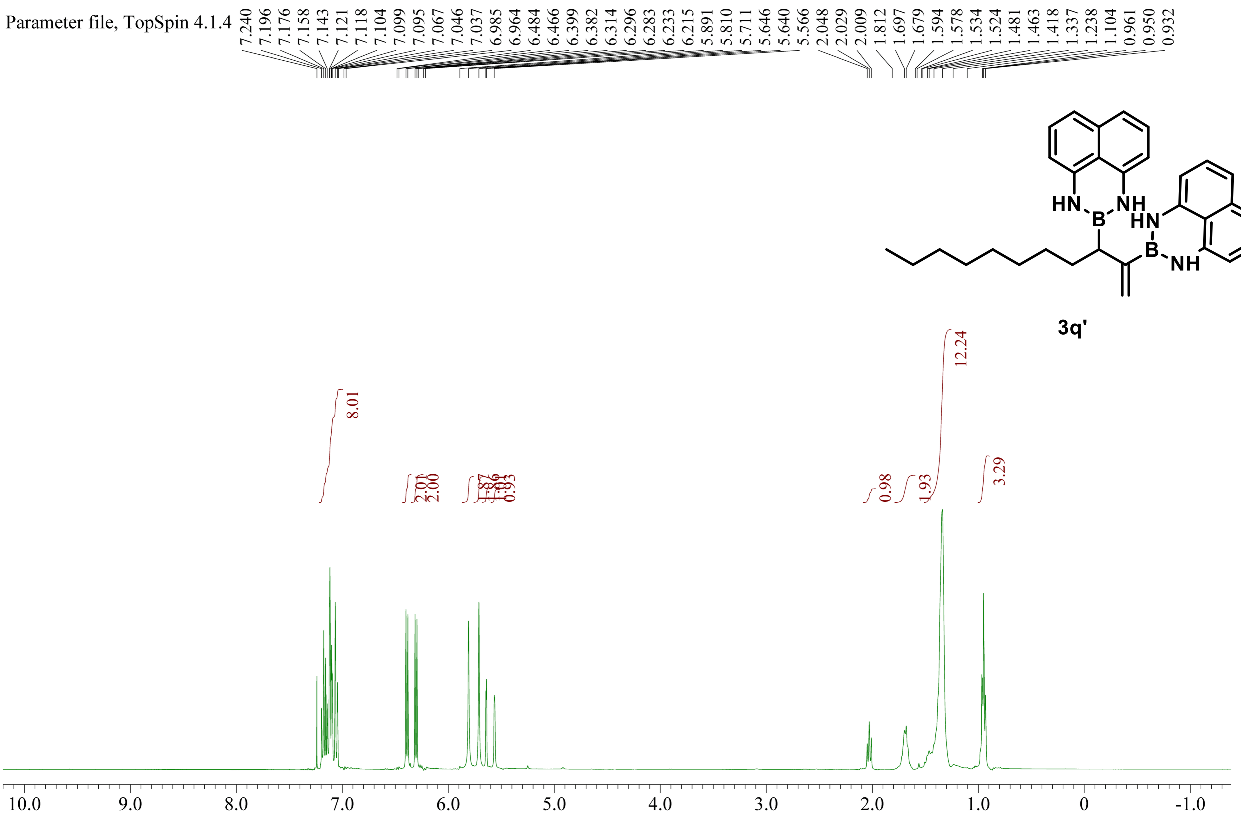

Figure S61. ^{1}H NMR ($CDCl_3$, 400 MHz) spectrum of **3q'**

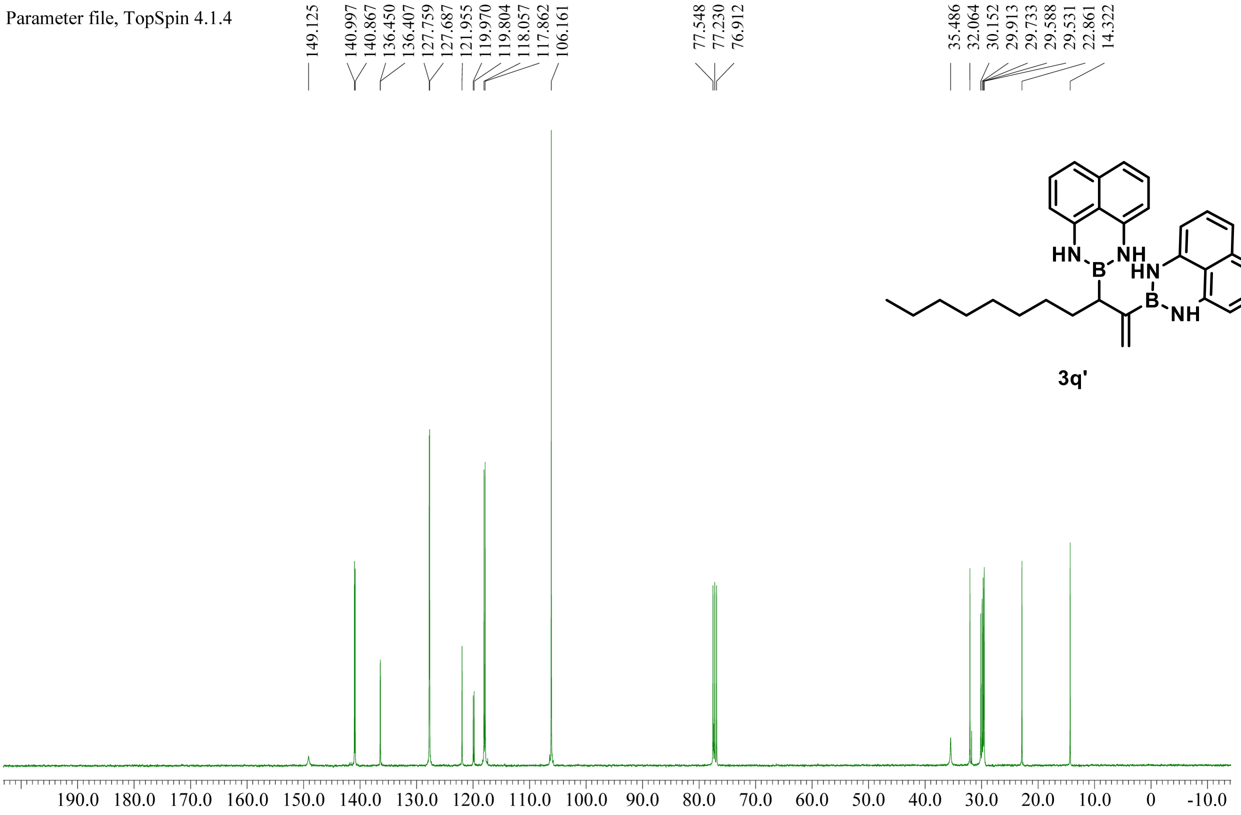

Figure S62. $^{13}C\{^{1}H\}$ NMR ($CDCl_3$, 100 MHz) spectrum of **3q'**

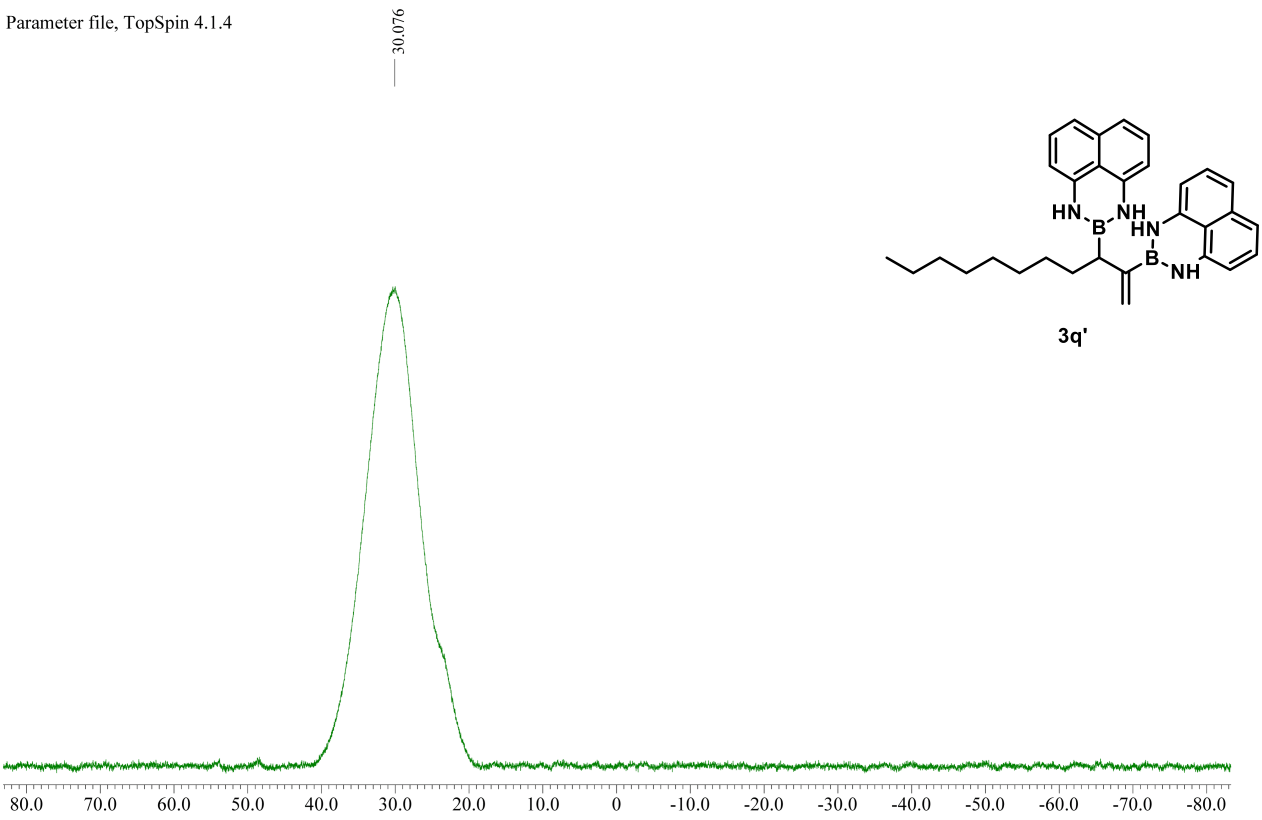

Figure S63. $^{11}B\{^{1}H\}$ NMR ($CDCl_3$, 128 MHz) spectrum of **3q'**

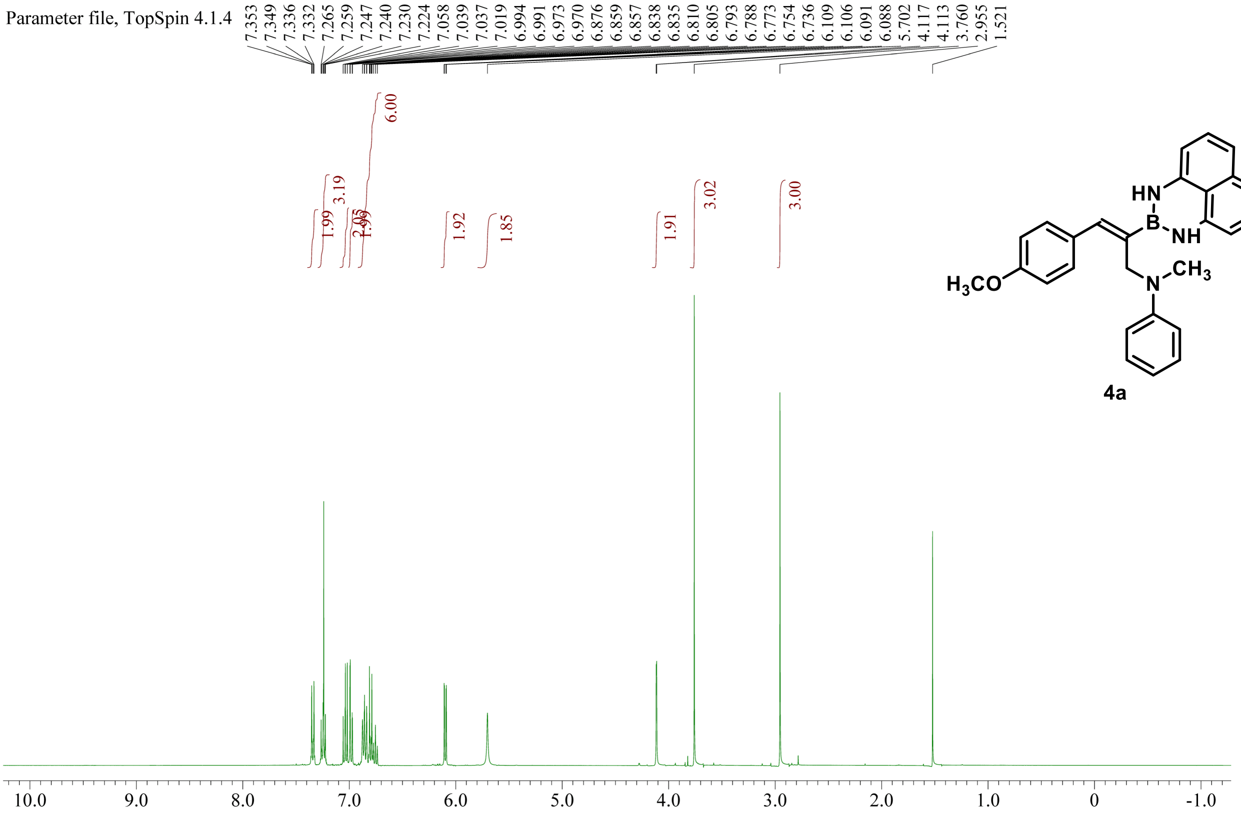

Figure S64. ^{1}H NMR ($CDCl_3$, 400 MHz) spectrum of **4a**

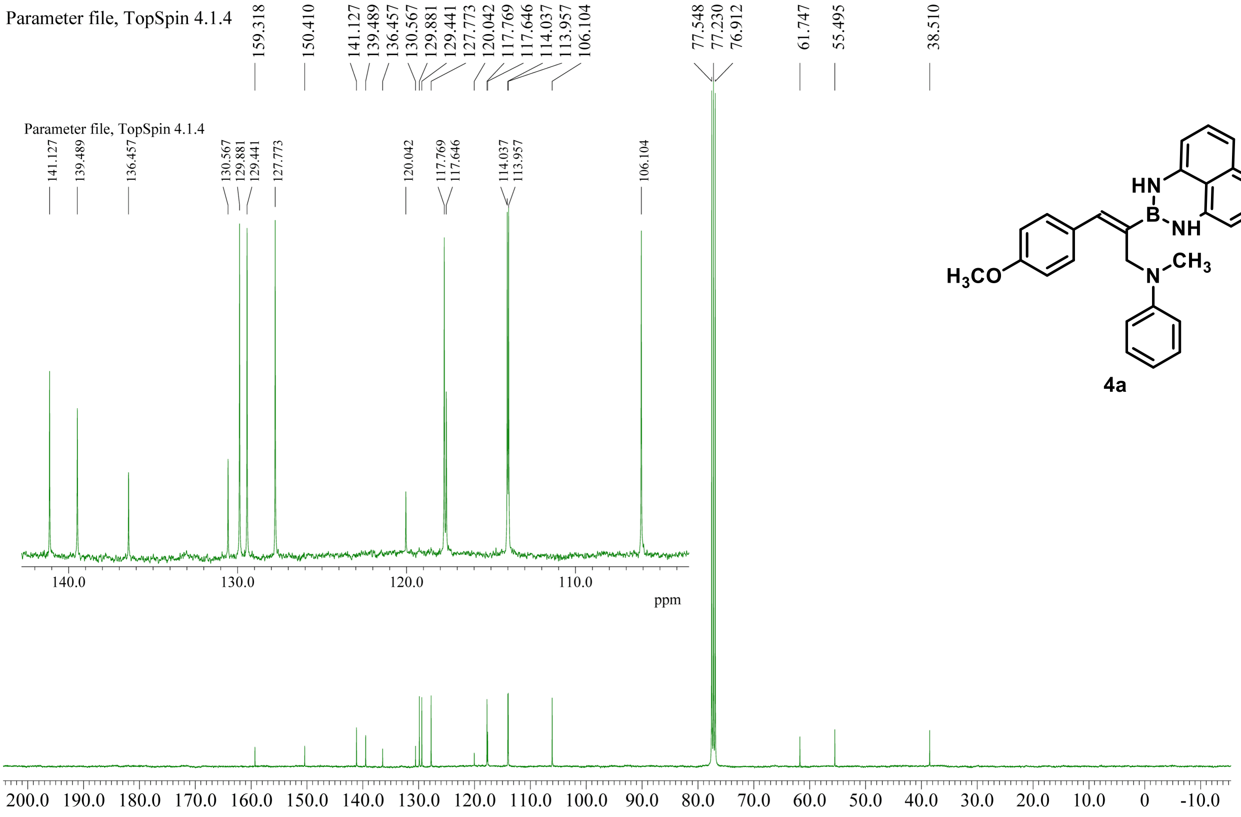

Figure S65. $^{13}C\{^{1}H\}$ NMR ($CDCl_3$, 100 MHz) spectrum of **4a**

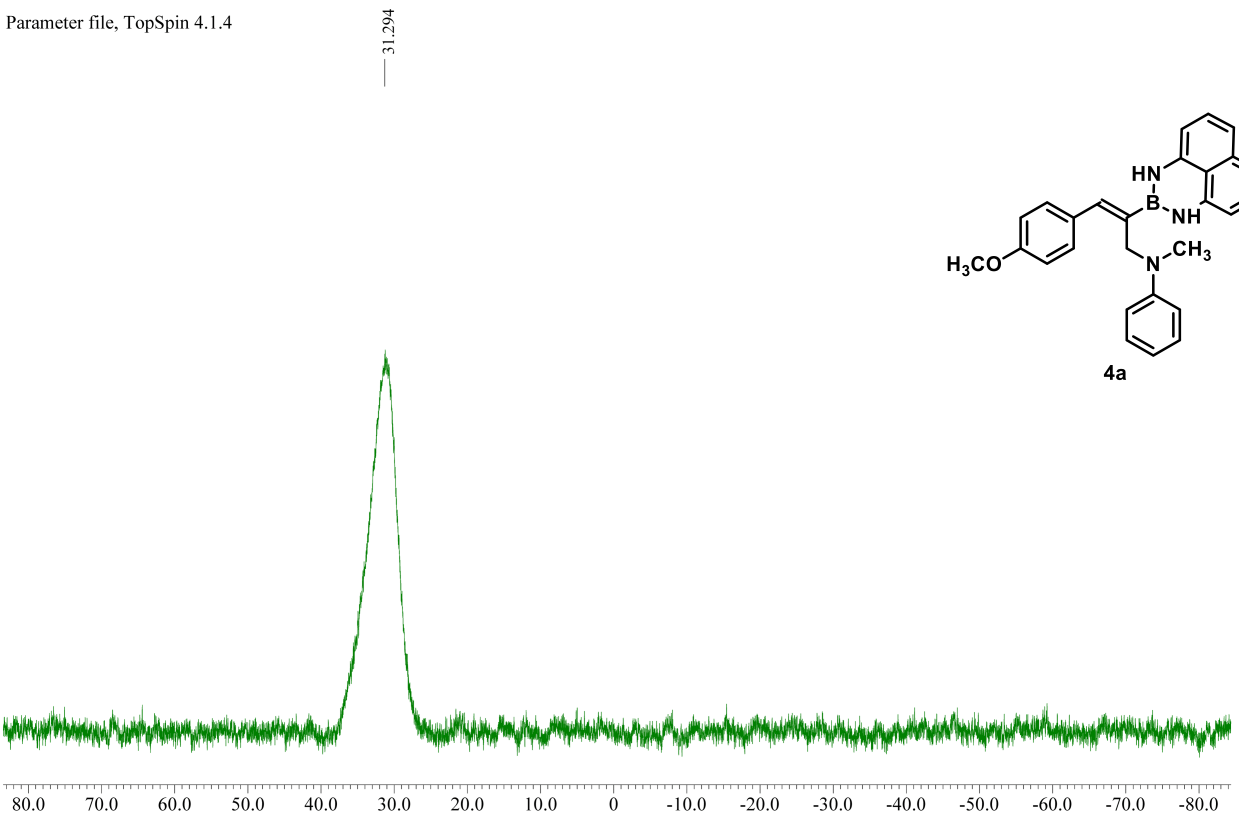

Figure S66. $^{11}B\{^1H\}$ NMR ($CDCl_3$, 128 MHz) spectrum of **4a**

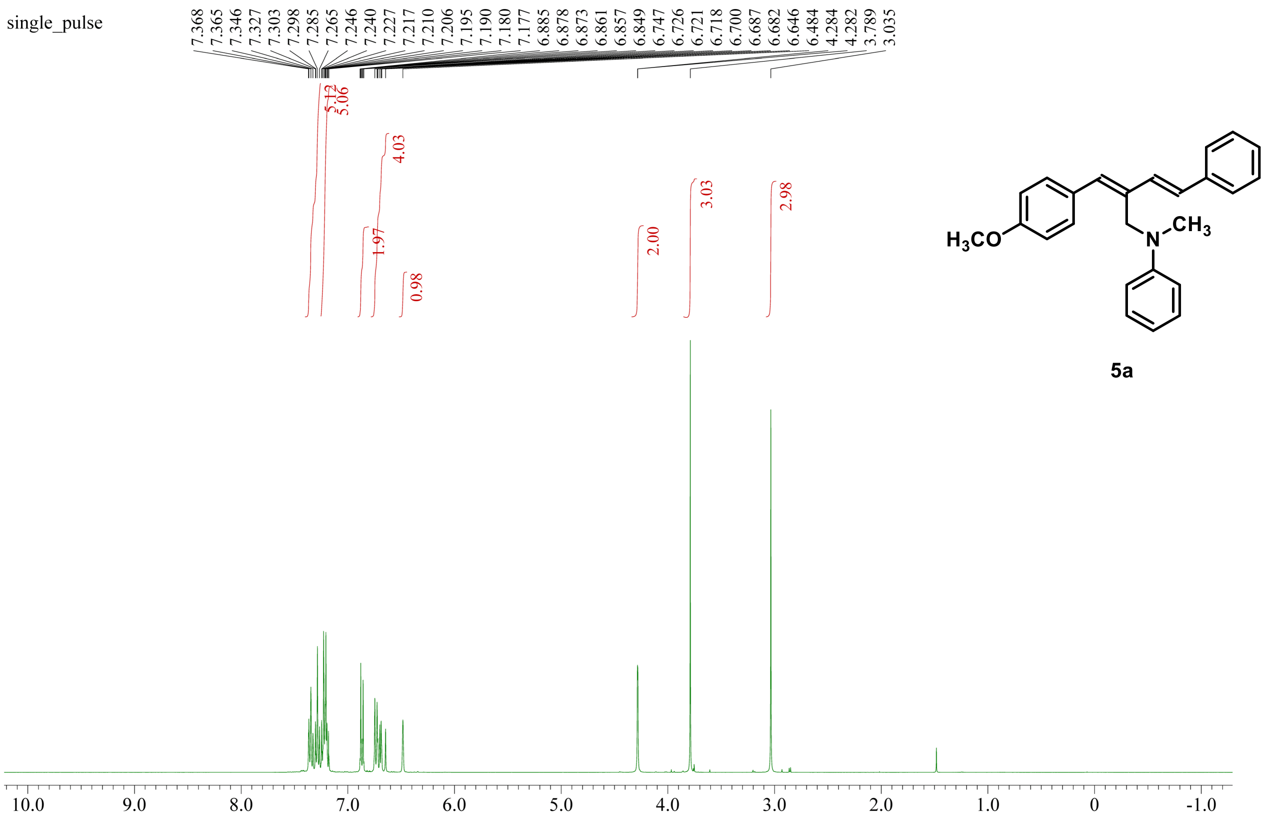

Figure S67. ^{1}H NMR ($CDCl_3$, 400 MHz) spectrum of **5a**

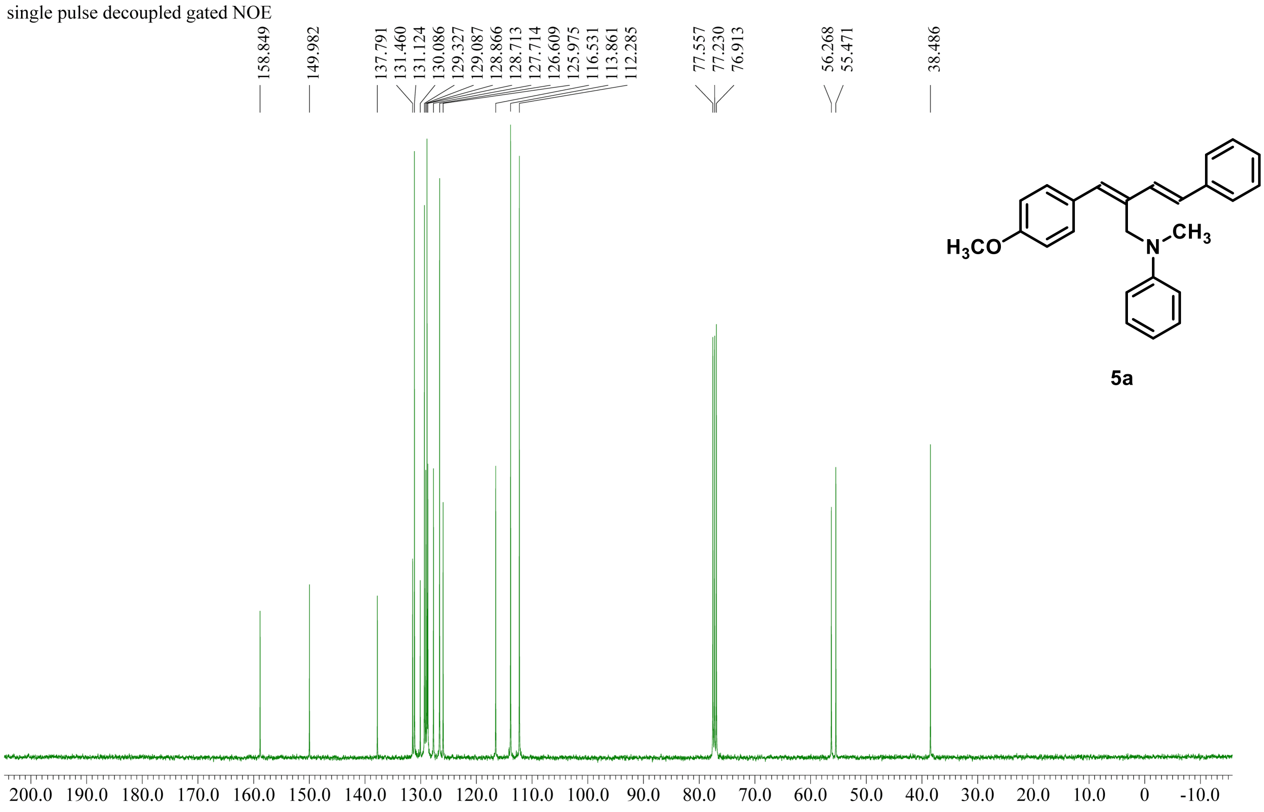

Figure S68. $^{13}C\{^{1}H\}$ NMR ($CDCl_3$, 100 MHz) spectrum of **5a**

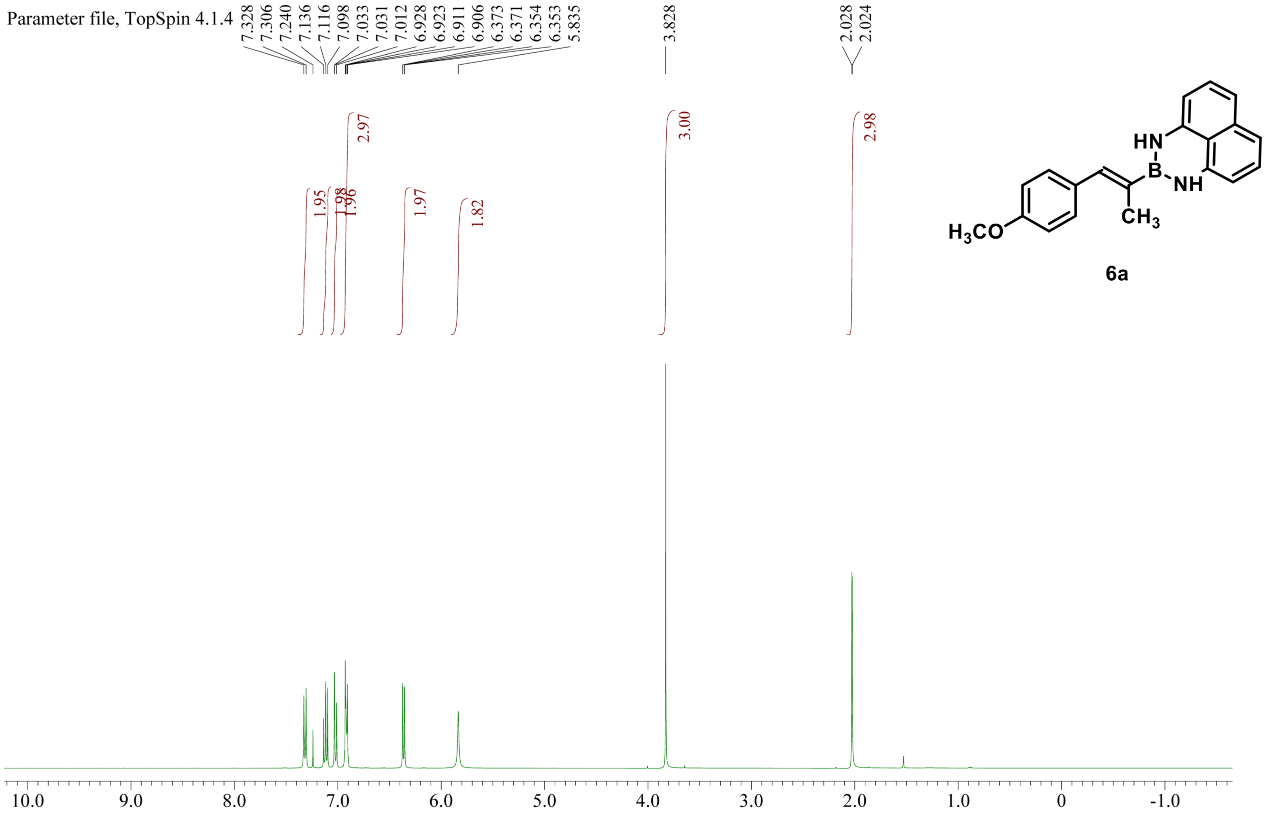

Figure S69. ^{1}H NMR ($CDCl_3$, 400 MHz) spectrum of **6a**

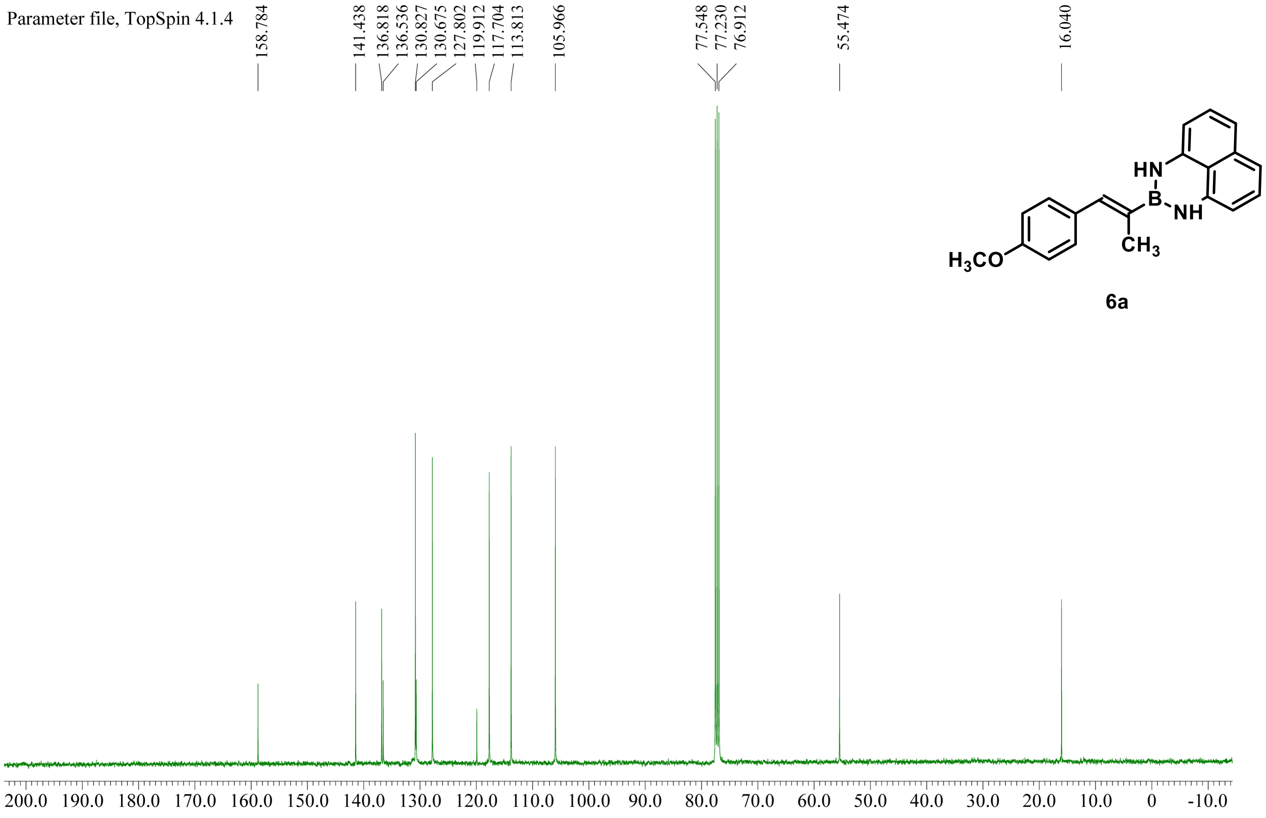

Figure S70. $^{13}C\{^{1}H\}$ NMR ($CDCl_3$, 100 MHz) spectrum of **6a**

Parameter file, TopSpin 4.1.4

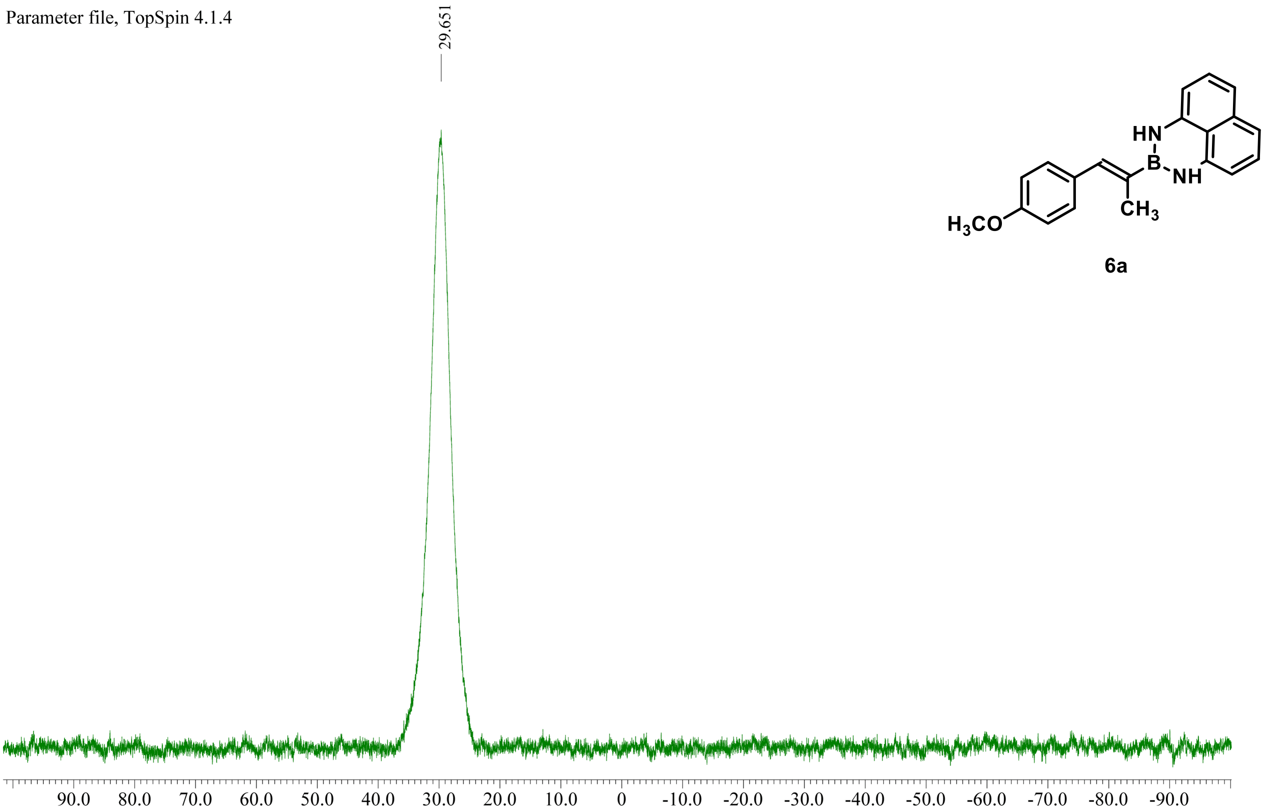

6a

Figure S71. $^{11}B\{^{1}H\}$ NMR ($CDCl_3$, 128 MHz) spectrum of **6a**

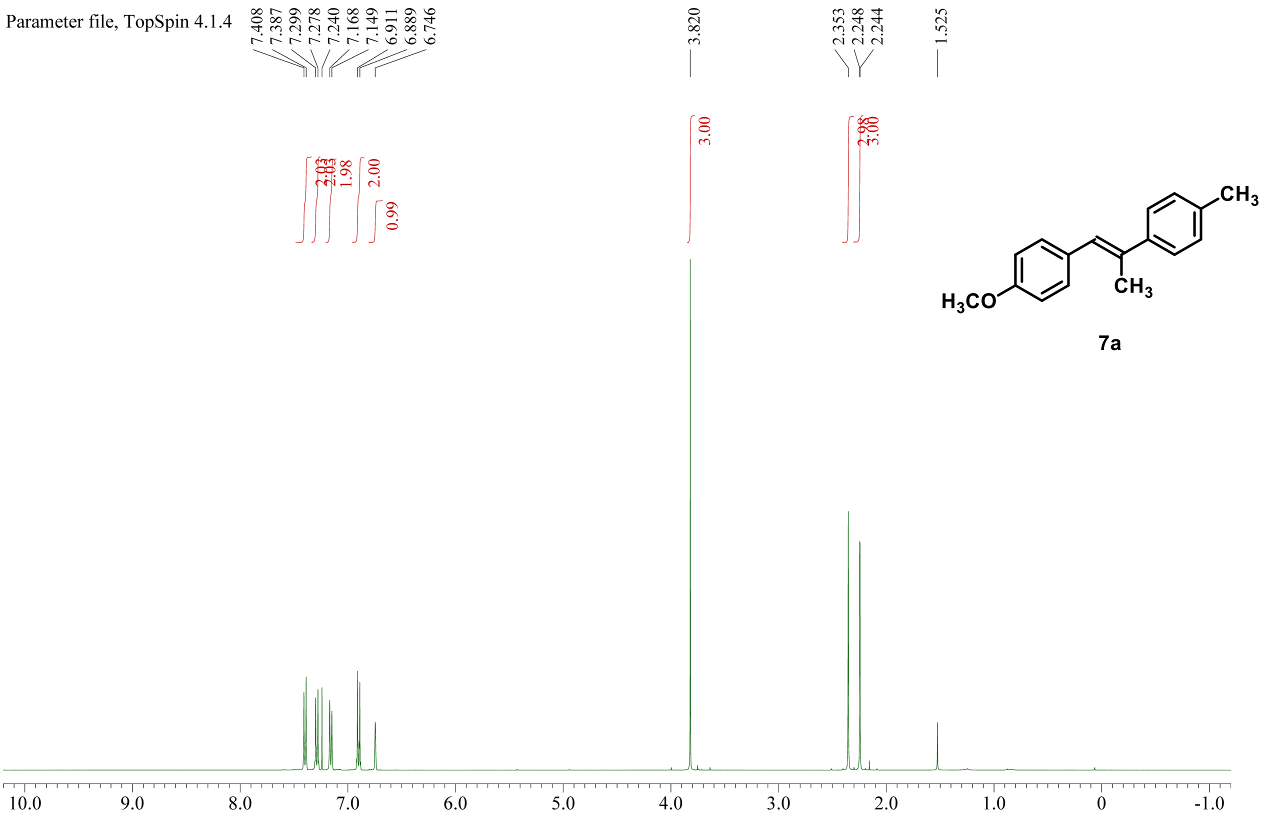

Figure S72. ^{1}H NMR ($CDCl_3$, 400 MHz) spectrum of **7a**
